# MDK Activates the PI3K/AKT Axis to Induce AP2A1 Expression and Epithelial–Mesenchymal Transition in Colorectal Cancer

**DOI:** 10.3390/cancers18081311

**Published:** 2026-04-21

**Authors:** Tengfei Li, Chengyuan Xu, Yang Guo, Yanyan Xu, Kaiji Chen, Yunsheng Cheng, Kesavamoorthy Gandhervin, Jianming Zhang, Moubin Lin

**Affiliations:** 1 Department of General Surgery, Yangpu Hospital Affiliated to Tongji University, Tongji University School of Medicine, Shanghai 200082, China; 2 General Surgery, Cancer Center, Department of Colorectal Surgery, Zhejiang Provincial People’s Hospital (Affiliated People’s Hospital, Hangzhou Medical College), Hangzhou 310014, China; 3 Department of General Surgery, Second Affiliated Hospital of Anhui Medical University, Anhui Medical University, Hefei 230601, China; 4 Department of Medicine, The University of Hong Kong, Hong Kong, China; 5 Department of Oncology, Pudong Hospital Affiliated to Fudan University, Fudan University School of Medicine, Shanghai 201399, China

**Keywords:** colorectal cancer, MDK, AP2A1, EMT, PI3K/AKT

## Abstract

**Background:** Midkine (MDK), a secreted heparin-binding growth factor, is involved in tumor progression and metastasis. While serum MDK is widely recognized as a potential prognostic biomarker for colorectal cancer (CRC), its specific functional role and underlying mechanisms in CRC development are not fully understood. **Methods:** The four publicly available CRC microarray datasets—GSE41258, GSE44076, GSE81558, and GSE117606—along with TCGA-COAD and TCGA-READ datasets and their associated clinical data were obtained. MDK expression was measured at both the mRNA and protein levels using quantitative real-time PCR (qRT-PCR) and Western blotting. To investigate its oncogenic functions, a comprehensive set of assays was performed: transwell and wound healing assays for invasion and migration; CCK-8 and colony formation assays for proliferation; and tail vein/spleen injection models combined with xenograft models to study metastasis and tumor growth in vivo. To uncover underlying mechanisms, Western blotting was used to examine the involvement of epithelial–mesenchymal transition (EMT) and the PI3K/AKT signaling pathway. **Results:** MDK is significantly overexpressed in CRC tissues and cells compared to normal tissues and cells. Notably, patients with high MDK levels show poorer overall survival (OS). Overexpression of MDK increases CRC invasion, migration, proliferation, and metastasis both in vivo and in vitro, while its knockdown reverses these effects. Mechanistically, MDK activates the PI3K/AKT pathway, leading to increased AP2A1 expression and promotion of EMT in CRC. **Conclusions:** MDK promotes invasion, migration, proliferation, metastasis, and EMT in CRC cells through the PI3K/AKT pathway by inducing AP2A1 expression, which could serve as a diagnostic marker. The PI3K inhibitor LY294002 significantly reduces AP2A1 levels and inhibits MDK-induced malignant behaviors. Targeting MDK-related signaling pathways may offer new strategies for CRC treatment.

## Simple Summary

Colorectal cancer is one of the most common cancers worldwide, and better treatments are urgently needed. In this study, we identified a protein called midkine as a key driver of colorectal cancer. We found that midkine promotes cancer cell invasion, migration, growth, and spread. It also triggers a process calle d epithelial–mesenchymal transition, which makes cancer cells more aggressive. Midkine works by activating a cellular communication pathway known as PI3K/AKT, which in turn increases the production of another protein called AP2A1. When we blocked midkine or used a drug that inhibits the PI3K/AKT pathway, these harmful effects were reduced. Our findings suggest that targeting midkine could lead to new therapies for colorectal cancer, offering hope for better treatment outcomes.

## 1. Introduction

Colorectal cancer (CRC) ranks as the third leading diagnosed cancer and the second most common cause of cancer death worldwide [1]. A major reason for the poor prognosis of CRC is the frequent failure to detect, diagnose, and treat the disease early on [2]. By the time clinical symptoms appear, most patients are already in advanced stages or have metastases, leading to a low survival rate. Besides surgical resection, radiotherapy, and other treatments, research into new biomarkers could provide additional therapeutic options for CRC in the future [3]. Understanding the mechanisms behind CRC development and progression is crucial.

Increased focus has been placed on the regulation of secretory proteins in tumors, especially in CRC. The human MDK gene, located at chromosome 11p11.2 and composed of five exons, encodes a small, secreted, heparin-binding growth factor protein [4]. Midkine (MDK) is a multifunctional protein that binds to cell-surface receptors, mimicking growth factors and activating key intracellular signaling pathways. Additionally, MDK may contribute to cancer development, as its expression is significantly upregulated in most tumors [5,6,7,8,9]. Mechanistically, MDK can promote a series of actions such as epithelial–mesenchymal transformation of cells, tumor angiogenesis, pro-fibrinolysis, and cell chemotaxis [4]. Interestingly, several studies have reported that MDK exhibits context-dependent functions across various cancer types. In gastric cancer, cancer-associated fibroblasts (CAFs) exert their effects through the secretion of MDK, which induces cisplatin resistance and facilitates epithelial–mesenchymal transition (EMT) [9], while also regulating AT1R expression and influencing growth and motility in HNSCC [8]. Although MDK is known to be upregulated in CRC tissues and correlates with poor prognosis, its specific role and the underlying molecular mechanisms—particularly whether MDK regulates EMT through the PI3K/AKT-AP2A1 axis—remain largely unexplored.

The EMT is characterized by the reprogramming of epithelial cells into a mesenchymal state, gaining enhanced abilities to invade and metastasize. This process enables tumors to spread beyond their primary location throughout the body [10]. Specifically, EMT results in malignant features of CRC, such as tumor budding, resistance, and immune escape [11,12,13,14]. However, whether and how MDK contributes to CRC development remains largely unknown. Therefore, we examined the effects of MDK on cell migration, invasion, proliferation, and EMT in CRC cells.

Here, we demonstrate through transcriptomic analysis that MDK positively regulates the expression of AP2A1, accompanied by increased levels of several EMT markers. Experimental results indicate that MDK enhances the proliferation, invasion, migration, and metastasis of CRC cells, primarily by activating the PI3K/AKT pathway, which then induces AP2A1 expression and promotes the EMT. In line with the observation that MDK is upregulated in multiple tumors, our findings suggest that MDK may serve as a valuable biomarker for both prognosis and therapeutic targeting in CRC.

## 2. Materials and Methods

### 2.1. Public Dataset

Four public microarray datasets for CRC were obtained from the Gene Expression Omnibus (GEO) (https://www.ncbi.nlm.nih.gov/geo/): GSE41258, GSE44076, GSE81558, and GSE117606. The transcriptomic data and corresponding clinical information for TCGA-COAD and TCGA-READ were sourced from The Cancer Genome Atlas (TCGA) (https://portal.gdc.cancer.gov/) portal.

### 2.2. Human CRC Patients and Specimens

This study collected CRC tissue and adjacent para-cancerous tissue samples from 55 matched fresh specimens. The cohort was recruited from Shanghai General Hospital (SGH) and included patients who underwent radical surgical resection for CRC from 2016 to 2018. For RNA extraction, samples were stored at −80 °C. Tissue microarrays (TMA) were developed using 106 pairs of CRC and adjacent non-neoplastic tissues. These samples were obtained from patients who underwent curative resection at SGH between 2013 and 2014, with all patients providing preoperative informed consent in accordance with the Ethics Review Board’s policies.

### 2.3. CRC Cell Lines and Cell Culture

HEK293T (CRL-11268), NCM460 (NCM460D™), and seven human CRC cell lines (RKO:CRL-2577, HT29:HTB-38, HCT116:CCL-247, DLD1:CCL-221, SW620:CCL-227, SW480:CCL-228, and LoVo:CCL-229) were obtained from the Chinese Academy of Sciences’ Culture Collection in Shanghai, China. We cultured HEK293T and CRC cell lines RKO, SW620, HCT116, and SW480 in DMEM (ShareBio, Shanghai, China) with 10% fetal bovine serum (FBS, Invigentech, São Paulo, Brazil) and 1% penicillin-streptomycin (P/S, PB180120). Meanwhile, HT29, LoVo, and DLD1 cells were maintained in RPMI-1640 (PM150110) supplemented with 10% FBS and 1% P/S under standard conditions (37 °C, 5% CO_2_ in a humidified incubator).

### 2.4. Real-Time Quantitative PCR (RT-qPCR)

Total RNA was purified from CRC samples and cell lines using TRIzol Reagent (Absin, Shanghai, China). Subsequently, reverse transcription was performed using the PrimeScript RT Master Mix to synthesize cDNA (Takara, Kusatsu, Shiga, Japan). For quantification, relative mRNA expression levels were measured using the 2^−ΔΔCt^ method, with GAPDH serving as the endogenous control for normalization. Each sample was tested in triplicate. All experiments were conducted with at least three independent biological replicates. PCR primers are listed in Supplementary Table S1.

### 2.5. Western Blotting

Proteins were extracted from both cell lines and tissue samples using RIPA buffer lysis (P0038, Beyotime Biotechnology, Shanghai, China). Protein concentration was measured with the BCA Protein Colorimetric Assay Kit (Elabscience, Wuhan, China). Samples were separated by SDS-PAGE and transferred to PVDF membranes (Millipore, Burlington, MA, USA). After blocking with 5% non-fat milk, the membranes were incubated with primary antibodies at 4 °C overnight. Protein bands were visualized with an enhanced chemiluminescence (ECL) kit and captured using a chemiluminescence detection system. All Western blot experiments were performed with three independent biological replicates. Band intensities were quantified with ImageJ (version 1.50b, National Institutes of Health, Bethesda, MD, USA), and exposure times were adjusted to stay within the linear range to prevent saturation bias. A list of the antibodies and reagents is provided in Supplementary Table S2.

### 2.6. Immunohistochemistry (IHC)

Tissue specimens subjected to immunohistochemistry (IHC) were fixed in 4% paraformaldehyde, then processed for paraffin embedding and sectioned. Immunostaining involved incubating the sections with primary antibodies at 4 °C overnight. A list of the antibodies and compounds is provided in Supplementary Table S2. After primary antibody incubation, the sections were treated with an HRP-labeled secondary antibody. Tissue sections were incubated with DAB to develop positive signals and then counterstained with hematoxylin. A composite IHC score (range 0–12) was calculated for each sample using the formula: Score = Staining Intensity × Percentage of Positive Cells. Staining intensity was graded 0–3 (0, negative; 1, weak; 2, moderate; 3, strong), and the percentage was scored 0–4 (0, 0%; 1, 1–25%; 2, 26–50%; 3, 51–75%; 4, 76–100%). Scores exceeding 4 indicated high MDK expression, while scores of 4 or less indicated low MDK expression.

### 2.7. Cell Transfection for RNA Interference

Commercially synthesized human MDK and AP2A1 siRNAs were purchased from Shanghai Generay Biotech Co., Ltd. (Shanghai, China), with detailed information listed in Supplementary Table S3. Transfection was performed using GyRransTM -RNA&DNA (GT1003, Shanghai Generay Biotech Co., Ltd., Shanghai, China) following the manufacturer’s instructions.

### 2.8. Establishment and Transfection of Stable Cell Lines

The plasmids pENTER, pEN-MDK, pCMV, and pCMV-AP2A1 were purchased from Shanghai Generay Biotech Co., Ltd. (Shanghai, China). MDK shRNAs (pLV[shRNA]-EGFP:T2A:Puro-U6 > hMDK) were obtained from VectorBuilder (Guangzhou, Guangdong, China). The plasmid pSLenti-EF1-EGFP-P2A-Puro-CMV-MDK-3xFLAG-WPRE was acquired from OBiO Technology (Shanghai) Corp., Ltd., Shanghai, China. The target plasmid was co-transfected with the lentiviral packaging plasmids psPAX2 and pMD2.G. The DNA construct was introduced into 293T cells using Lipo293™ reagent (C0521, Beyotime Biotechnology, Shanghai, China). Virus particles were collected from the culture medium after 48 h and used to infect RKO and SW480 cells, creating control and MDK knockout stable cell lines (SW480-MDK-NC/KD, RKO-MDK-NC/KD). Puromycin (ST551, Beyotime Biotechnology) was used to select cells with successful MDK knockdown. In addition, empty vectors and MDK-overexpressing stable cells (SW620-Vector/MDK, DLD1-Vector/MDK) were established through the same method.

### 2.9. Cell Viability and Transwell Assays

Cell Counting Kit-8 was used to assess cell viability (CellorLab CX001L, Shanghai, China). For transwell assays, each group was seeded with 4 × 10^4^ cells into the upper chamber (3422, 8.0 μm pore, Corning, NY, USA) along with 200 μL of serum-free medium. A transwell assay was performed to evaluate cell migration and invasion. The lower chamber contained 500 μL of DMEM with 10% FBS as a chemoattractant. After a 36 h incubation, cells on the upper membrane were fixed with 4% paraformaldehyde and stained with 0.1% crystal violet. To assess clonogenic potential, cells were seeded into six-well plates at a density of 1000 cells per well for colony formation. After a 2-week incubation, colonies were fixed and stained with crystal violet for analysis. To evaluate cell migration, a wound healing assay was conducted. Briefly, cells were cultured in six-well plates until a complete monolayer formed. Uniform wounds were created with a sterile 200-μL pipette tip. Following PBS washes to remove detached cells, serum-free medium was added to inhibit proliferation, and wound closure was imaged at regular intervals. Representative images were captured using a microscope and quantified at 0, 24, and 48 h. EdU assays were performed using the CX003L EdU-555 (catalog no. CX003L, EpiZyme, Shanghai, China) according to the manufacturer’s instructions. All functional assays were conducted with at least three independent biological replicates, each with technical triplicates.

### 2.10. Mice Model

BALB/c nude mice (male, 4–5 weeks old) were obtained from Cyagen Biosciences Inc. (Suzhou, China) and randomly assigned to two groups. Investigators were blinded to group allocation during tumor volume measurement and data analysis. Subcutaneous xenografts were created by inoculating mice with 1 × 10^7^ SW480 cells (either MDK-knockdown or control variants) in 100 μL PBS. Tumor volume was regularly measured using a caliper and calculated with the formula: 0.5 × length × width^2^. Twenty-five days after injection, mice were euthanized, and tumor tissues were collected for qRT-PCR, Western blotting, and IHC analyses. A lung metastasis model was established by injecting 1 × 10^7^ stable transfected SW480 cells intravenously via the tail vein. Four weeks later, mice were euthanized. Lung tissues were excised, examined for metastatic nodules, fixed in formalin, embedded in paraffin, and sectioned. Hematoxylin and eosin (H&E) staining was performed for histopathological analysis. For the liver metastasis study, 5 × 10^6^ cells suspended in 50 μL PBS were injected into the splenic capsule. At the endpoint (4 weeks later), mice were sacrificed, and livers were examined for metastatic nodules. The entire animal study protocol was reviewed and approved by the Institutional Animal Care and Use Committee at Yangpu Hospital, Tongji University, in accordance with their guidelines (code: TJBF00925107, date: 25 February 2025).

### 2.11. Statistical Analysis

All statistical analyses were conducted using SPSS 24.0. Associations between MDK expression and clinicopathological features were assessed with Pearson’s chi-square test. Overall survival was evaluated with Kaplan–Meier curves and compared using the log-rank test. Continuous variables were compared between two groups with Student’s *t*-test (paired or unpaired) and among multiple groups using one-way ANOVA. A two-sided *p*-value of less than 0.05 was deemed statistically significant.

## 3. Results

### 3.1. Aberrant MDK Expression in Various Tumors and Methylation Analysis

**Figure 1:**
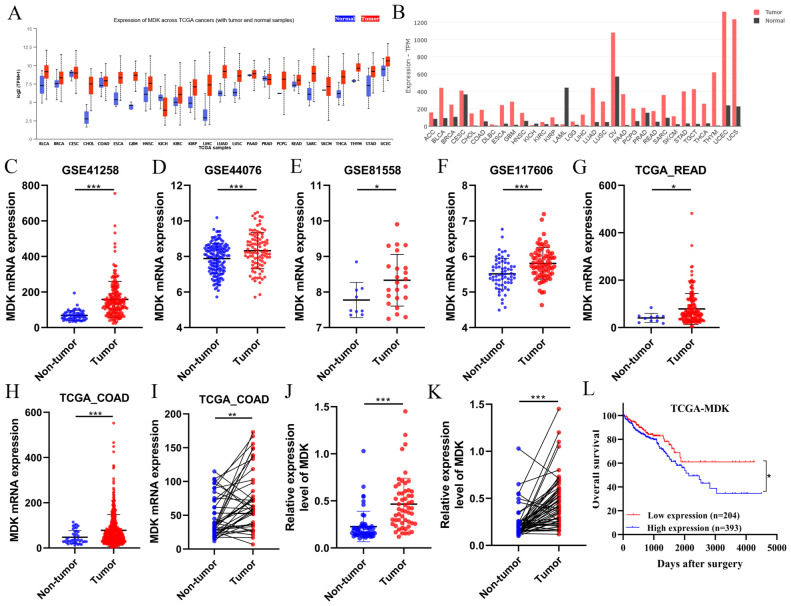
MDK mRNA expression profiles across cancers and in CRC. (**A**,**B**) We examined MDK expression patterns across various cancers using the UALCAN and GEPIA2 platforms. (**C**–**I**) Comparative analysis of MDK transcripts in CRC versus normal tissues from GEO, TCGA, and in-house (SGH) databases. (**J**,**K**) qRT-PCR validation of MDK upregulation in 55 paired CRC/normal tissues. (**L**) Kaplan–Meier survival curve for the TCGA cohort based on MDK expression levels. Significance levels: * *p* < 0.05, ** *p* < 0.01, *** *p* < 0.001.

To investigate MDK expression in CRC and its clinical significance, we analyzed MDK mRNA levels across different cancer types in paired tumor-normal samples, using the UALCAN and GEPIA2 public databases. The UALCAN analysis showed elevated MDK levels in 22 of 24 tumor types compared to normal tissues (Figure 1A), a result confirmed by independent analysis with GEPIA2 (Figure 1B). We then examined MDK expression in paired normal and tumor tissues in GEPIA2 and found high expression in 29 tumors (Figure S1A–F) and low expression in 2 tumors (Figure S1D). Similarly, MDK was highly expressed in TCGA-COAD (Figure S1E). Using the TIMER database, which compiles RNA-seq data from various cancers, we observed a distinct MDK expression profile in COAD, with significantly higher mRNA levels in tumors versus normal tissues. Elevated MDK expression was found across multiple tumor types, indicating widespread upregulation in cancers (Figure S1G). At the protein level, MDK also showed high expression in COAD compared to adjacent tissues (Figure S1H). It is important to note that not all gene variants affect gene expression similarly, although variants may influence expression in different diseases. Therefore, we investigated MDK epigenetic regulation across all tumors. We found hypermethylation in BRCA, COAD, KIRP, SARC, TCGT, and LUSC in 6 of 29 tumors with high MDK expression (Figure S2A–F), while PAAD, UCEC, LIHC, PRAD, and PCPG showed hypomethylation in 29 high-MDK tumors (Figure S2G–K). Twelve probes targeting the DNA methylation of MDK are located in its promoter region (Figure S2L). These results suggest that MDK may function as an oncogene in CRC.

### 3.2. High MDK Expression Is Correlated with Poor COAD Prognosis

To compare MDK expression between tumor specimens and adjacent normal tissues, we analyzed RNA-seq profiles from public repositories, including four GEO datasets and TCGA-COAD. MDK mRNA levels were significantly higher in tumor samples across all six public cohorts (Figure 1C–H), a finding validated in paired TCGA-COAD samples (Figure 1I), and further confirmed by qRT-PCR in our 55-patient cohort (Figure 1J,K). Kaplan–Meier analysis of the TCGA database showed that high MDK expression was linked to significantly reduced overall survival (OS) compared to the low-expression group (*p* < 0.05; Figure 1L). These findings, along with consistent multi-database evidence, support the hypothesis that MDK has an oncogenic role in tumor development and progression.

Given that elevated MDK expression is associated with unfavorable outcomes in CRC patients, we aimed to investigate its specific effects on the COAD cohort. Clinicopathological features and MDK expression levels were analyzed in the TCGA cohort. To evaluate the prognostic value of MDK, we used an optimal expression cut-off (derived from survival analysis of FPKM data) to classify patients into MDK-high and MDK-low subgroups within the TCGA COAD cohort. Interestingly, we observed MDK signal in 95 of 106 specimens (53 strongly positive, 42 weakly positive) (Figure S3A). MDK expression showed a significant positive correlation with N stage (*p* = 0.001), differentiation (*p* = 0.023), and TNM stage (*p* < 0.001) (Figure S3B–D, Supplementary Table S4). However, MDK levels did not significantly correlate with age, gender, tumor stage, or tumor size (Supplementary Table S4). IHC analysis of TMA indicates that increased MDK expression plays a role in CRC pathogenesis and progression. In the chi-square test, MDK mRNA levels correlated with patient survival status (Table 1, bottom, *p* = 0.027). After excluding 159 TCGA patients lacking TNM data, 438 patients remained. Within this group, a significant association was found between N stage and MDK expression levels (Supplementary Table S6, *p* = 0.023). The prognostic impact of MDK was validated in a cohort of 106 CRC tissues. Consistently, higher MDK expression was linked to poorer overall survival (OS) and disease-free survival (DFS) (Figure S3E,F), a finding supported by the TCGA-COAD cohort analysis (Figure 1L). Univariate analysis identified MDK as an independent adverse prognostic factor for OS (HR = 1.519, *p* = 0.042; Table 2). Multivariate survival analysis using the Cox proportional hazards model showed that N and M stages were significantly associated with OS, with hazard ratios of 1.758 (*p* = 0.035) and 2.589 (*p* < 0.001), respectively. Prognostic analysis revealed that high MDK expression independently predicted worse OS and DFS in our 106-patient cohort (all *p* < 0.05; Supplementary Table S5). This relationship was also observed in the TCGA-COAD cohort (Table 2). Therefore, MDK is a significant and reproducible prognostic factor in CRC.

### 3.3. MDK Promotes Cell Invasion and Metastasis In Vivo

**Figure 2:**
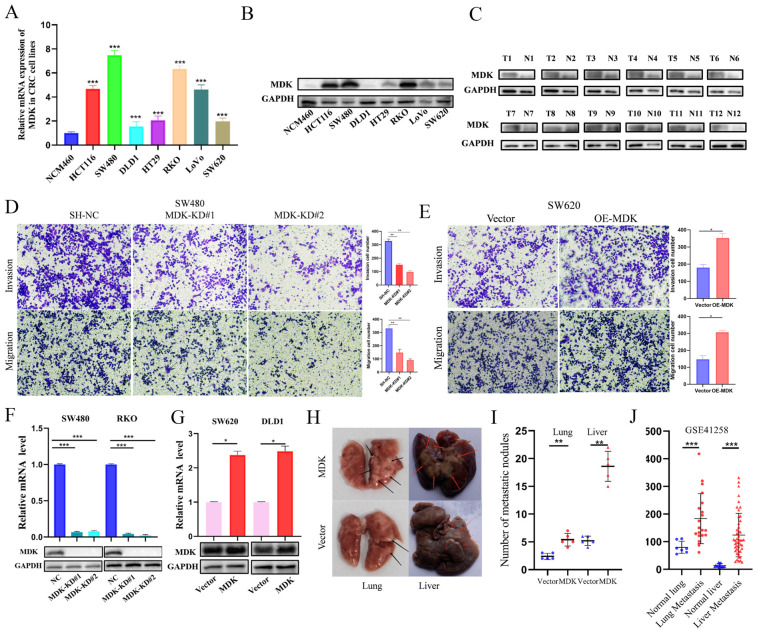
MDK promotes CRC cell growth and metastasis both in vitro and in vivo. (**A**,**B**) MDK expression was measured in seven CRC cell lines and the normal colonic epithelial cell line NCM460 using Western blot and qRT-PCR. (**C**) MDK protein levels in paired CRC and adjacent normal tissues were analyzed by Western blot. (**D**,**E**) The invasion and migration abilities of SW480 (with knockdown) and SW620 (with overexpression) cells were assessed. ×100 magnification; scale bar not shown. (**F**,**G**) The effectiveness of MDK knockdown (in SW480 and RKO) and overexpression (in DLD1 and SW620) was confirmed by Western blot and qRT-PCR. (**H**,**I**) A lung metastasis model was created via tail vein injection. Representative images (**H**) and quantification (**I**) of lung metastatic nodules are displayed. Black arrows indicate lung metastatic nodules; red arrows indicate liver metastatic nodules. (**J**) MDK mRNA expression in lung metastasis samples from the GSE41258 dataset was analyzed. * *p* < 0.05, ** *p* < 0.01, *** *p* < 0.001. The uncropped blots are shown in File S1.

To clarify the impact of MDK expression on CRC cell proliferation, we analyzed MDK mRNA and protein levels in human normal colonic epithelial NCM460 cells and seven CRC cell lines using qRT-PCR and Western blotting. MDK was significantly higher in SW480 and RKO cell lines compared to the other CRC cell lines, while MDK expression was lowest in DLD1 and SW620 compared to the remaining CRC cell lines (Figure 2A,B). To define the functional roles of MDK, we generated stable knockout lines in RKO and SW480 cells and overexpression lines in DLD1 and SW620 cells. Consistently, Western blot analysis of 12 paired clinical samples showed a significant upregulation of MDK protein in tumor tissues compared to matched adjacent normal tissues (Figure 2C). Transwell assays demonstrated that MDK knockdown significantly reduced invasion and migration in SW480 and RKO cells (Figure 2D), whereas MDK overexpression enhanced these behaviors in SW620 and DLD1 cells (Figure 2E). To explore MDK’s roles in CRC cell growth and metastasis, we modulated its expression using lentiviral transduction. Specifically, stable knockdown was achieved in SW480 and RKO cells (Figure 2F), while overexpression was established in SW620 and DLD1 cells (Figure 2G). For in vivo metastatic potential, xenograft mouse models for lung and liver metastasis were developed via tail vein and intrasplenic injection of SW620 cells with either vector or OE-MDK. Furthermore, these metastasis models showed that MDK overexpression led to significantly more metastatic foci than control groups (Figure 2H,I), similar to sequencing results from patients with colon tumors at Memorial Sloan-Kettering Cancer Center (1992–2004) (Figure 2J). Thus, MDK is confirmed as a key driver of CRC growth and metastasis.

### 3.4. MDK Promotes the Proliferation and Migration in CRC Cells

**Figure 3:**
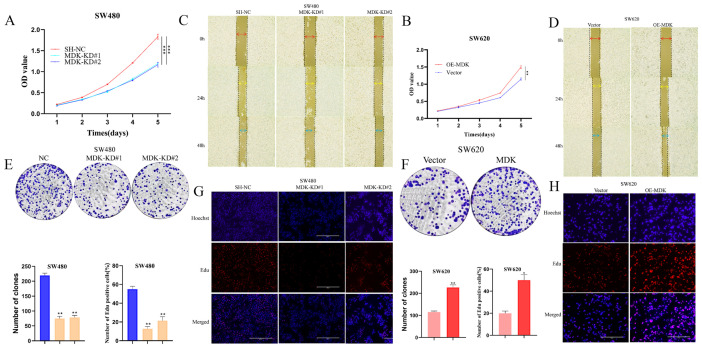
MDK promoted CRC cell proliferation and migration. (**A**) MDK downregulation inhibited the proliferation of SW480 cell. (**B**) MDK upregulation promoted the proliferation of SW620 cell. (**C**) MDK downregulation inhibited the migration of SW480 cell. ×100 magnification; scale bar not shown. (**D**) MDK upregulation promoted the migration of SW620 cell. ×100 magnification; scale bar not shown. (**E**) MDK downregulation weakened colony formation in SW480 cell and the number of colonies were calculated. ×20 magnification; scale bar not shown. (**F**) MDK upregulation enhanced colony formation in SW620 cell and the number of colonies were calculated. ×20 magnification; scale bar not shown. (**G**,**H**) Cell proliferation was assessed by EdU assay in SW480 and SW620 cells, with representative images shown. ×100 magnification. Arrow colors (**C**,**D**): red = 0 h, yellow = 24 h, blue = 48 h. Bar colors: blue = shNC, yellow = MDK-KD, pink = Vector, red = OE-MDK. * *p* < 0.05, ** *p* < 0.01, *** *p* < 0.001.

CCK-8, wound healing, colony formation, and EdU assays consistently showed that knocking down MDK suppressed proliferation, migration, and clonogenicity in SW480 and RKO cells (Figure 3A,C,E,G and Figure S4A,C,E,G), while overexpressing MDK enhanced these phenotypes in SW620 and DLD1 cells (Figure 3B,D,F,H and Figure S4B,D,F,H). Consistent with its role in promoting clonogenicity, MDK knockdown reduced colony formation in SW480 and RKO cells (Figure 3E and Figure S4E). This was supported by the opposite effect seen with MDK overexpression in SW620 and DLD1 cells (Figure 3F and Figure S4F). Additionally, EdU assay results indicated that knocking down MDK inhibited proliferation in SW480 and RKO cells (Figure 3G and Figure S4G), while overexpressing MDK increased proliferation in SW620 and DLD1 cells (Figure 3H and Figure S4H). Overall, our results establish MDK as a key promoter of CRC proliferation and migration.

### 3.5. MDK Induced EMT via PI3K/AKT Pathway Activation in CRC Cells

**Figure 4:**
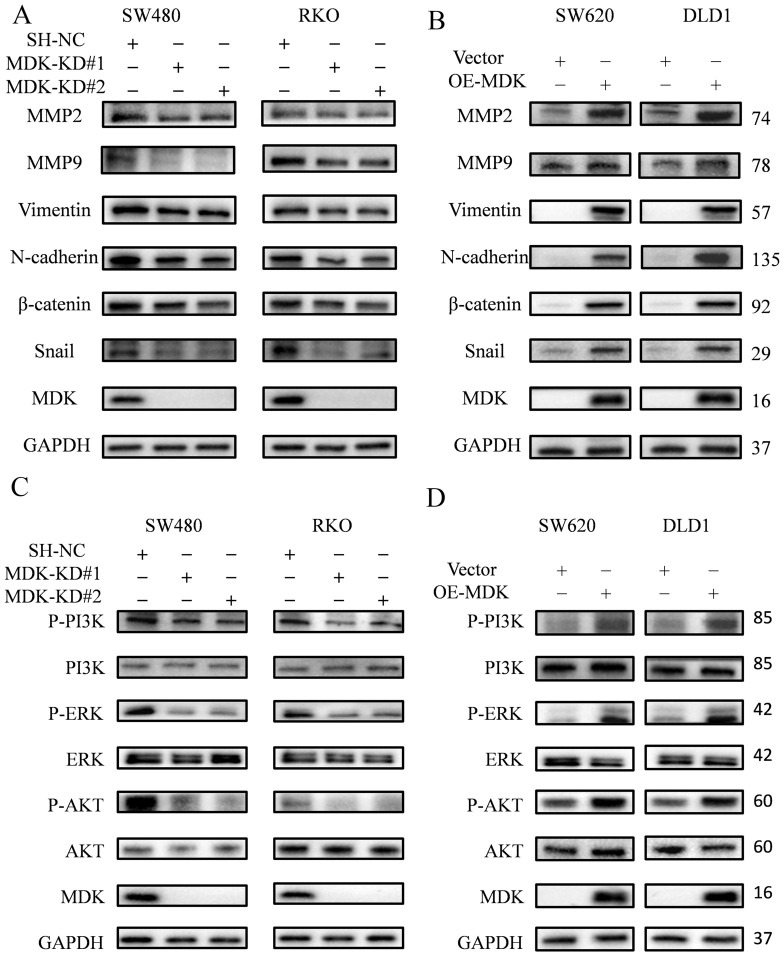
MDK promotes EMT in CRC cells through activation of the PI3K/AKT pathway. (**A**,**B**) The expression of EMT-related proteins was measured by Western blot in MDK-knockdown (SW480, RKO) and MDK-overexpression (SW620, DLD1) cells, respectively. (**C**,**D**) Activation of the PI3K/AKT pathway was evaluated in the same sets of cells following MDK knockdown or overexpression. The uncropped blots are shown in File S1.

The invasion, migration, proliferation and metastasis of tumor cells in vivo are all closely associated with EMT [8,15]. We demonstrated that MDK could induce EMT by modulating the PI3K/AKT signaling pathway. Research indicates that CAF-derived MDK enhances EMT-mediated DDP resistance in gastric cancer by upregulating ST7-AS1 and activating the PI3K/AKT pathway [8]. MDK knockdown suppresses hypoxia-induced proliferation, migration and EMT in GBM cells [15]. Due to the strong correlation between increased invasion, migration, proliferation capabilities, and EMT, we postulate that MDK might regulate the expression of MMP2, MMP9, Vimentin, N-cadherin, and β-catenin through PI3K/AKT modulation. Therefore, we examined EMT protein markers in CRC cells. MDK knockdown in SW480 and RKO cells led to a decrease in multiple EMT markers, such as N-cadherin, Vimentin, β-catenin, Snail, and matrix metalloproteinases MMP2 and MMP9, with major bands detected at approximately 74 kDa (MMP2) and 78 kDa (MMP9) consistent with antibody specifications (Figure 4A). Conversely, MDK overexpression produced the opposite effects on EMT markers (Figure 4B). Similarly, Western blot analysis showed that MDK knockdown was accompanied by significantly reduced phosphorylation of PI3K, AKT, and ERK, correlating with the observed phenotypic changes and suggesting that MDK promotes CRC progression by activating these pathways (Figure 4C). As expected, overexpression of MDK yielded opposite results in SW620 and DLD1 cells (Figure 4D). Overall, our findings demonstrate that MDK contributes to CRC progression by activating PI3K/AKT signaling, which in turn induces EMT.

### 3.6. MDK Enhanced CRC Cell Invasion, Migration and Proliferation by Increasing AP2A1 Expression

**Figure 5:**
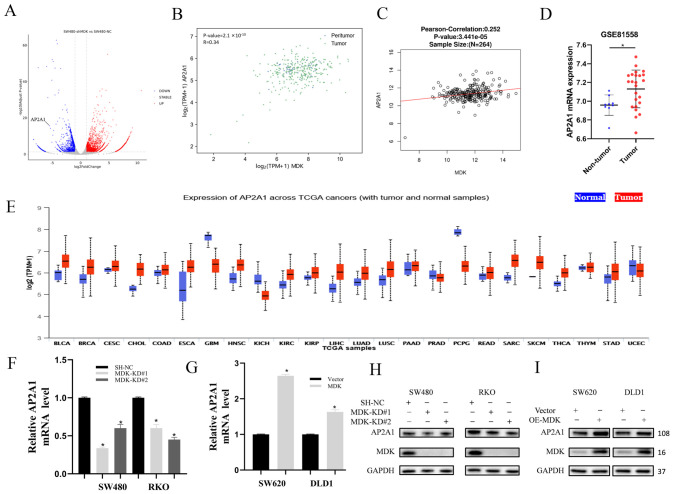
Correlation between MDK and AP2A1 and the expression of AP2A1 in CRC tissues and cells. (**A**) Volcano plot of RNA-seq data showing genes differentially expressed upon MDK knockdown (sh-MDK vs. sh-NC). Down- and up-regulated genes are highlighted in blue and red, respectively. (**B**,**C**) Correlation analyses between MDK and AP2A1 expression in the TCGA (**B**) and LinkedOmics (**C**) CRC cohorts. (**D**,**E**) Validation of MDK ((**D**), GSE81558) and AP2A1 ((**E**), TCGA) upregulation in CRC tumors compared to normal tissues. (**F**–**I**) MDK regulates AP2A1 expression. AP2A1 mRNA (**F**,**G**) and protein (**H**,**I**) levels were assessed by qRT-PCR and immunoblotting in cells with MDK overexpression or knockdown. Data are presented as mean ± SD; * *p* < 0.05, (Student’s *t*-test). The uncropped blots are shown in File S1.

To clarify the molecular mechanisms downstream of MDK, we conducted RNA-seq on SW480 cells after MDK knockdown. The analysis identified 3155 differentially expressed genes (DEGs) compared to sh-NC controls, including 1186 downregulated and 1969 upregulated transcripts (Figure 5A). Supplementary Table S7 provides the full list of DEGs. Research has shown that AP2A1 can directly bind to circEIF3I to promote PDAC cell migration, invasion, and metastasis [16]. Therefore, from the invasion- and metastasis-related genes affected by RNA-seq, AP2A1 was highlighted as a candidate due to its consistent presence across relevant gene sets (Supplementary Table S7). Bioinformatic analysis of the GEPIA 3 and LinkedOmics databases revealed a significant positive correlation between MDK and AP2A1 levels, supporting the idea that MDK regulates AP2A1 expression (Figure 5B,C). Consistent with its oncogenic role, AP2A1 mRNA was elevated in tumor samples from the GSE1558 cohort (Figure 5D) and across various cancer types, especially in COAD (Figure 5E). Functionally, knocking down MDK in SW480 and RKO cells decreased AP2A1 mRNA, while overexpressing MDK in SW620 and DLD1 cells increased it (Figure 5F,G). Western blot analysis confirmed that MDK consistently influences AP2A1 protein expression (Figure 5H,I), validating the RNA-seq data at the protein level.

**Figure 6:**
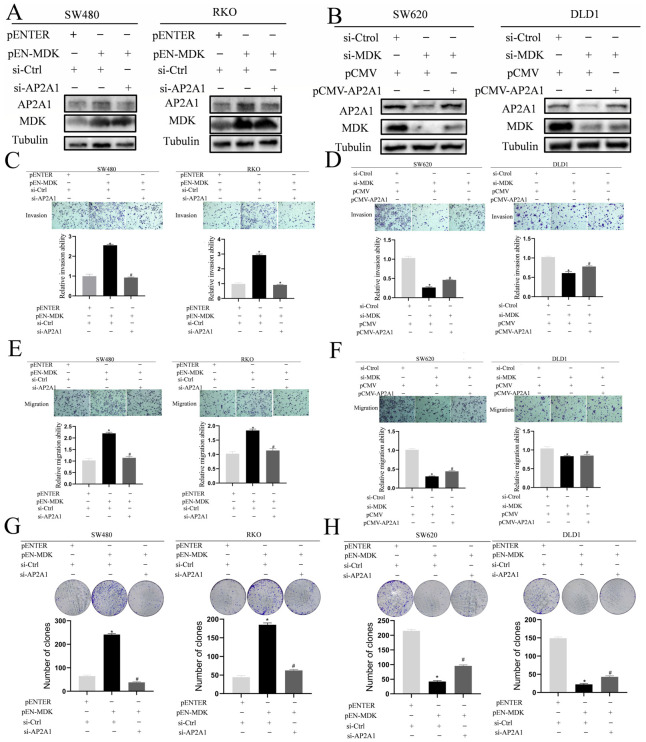
AP2A1 functions as a downstream regulator of MDK-driven CRC invasion. (**A**,**B**) Immunoblotting verified that altering MDK levels correspondingly changes AP2A1 expression. Band intensities were quantified and normalized to Tubulin. (**C**–**F**) Functional rescue experiments showed that the increased invasion/migration caused by MDK overexpression (or the decrease caused by its knockdown) was reversed by co-manipulating AP2A1, as demonstrated by Transwell assays (with or without Matrigel). ×100 magnification; scale bar not shown. (**G**,**H**) Likewise, MDK-induced cell growth phenotypes were affected by changing AP2A1 levels. ×20 magnification; scale bar not shown. * *p* < 0.05 vs. control; # *p* < 0.05 vs. MDK-manipulated group (Student’s *t*-test). The uncropped blots are shown in File S1.

To establish a direct functional connection, we conducted reciprocal rescue experiments by genetically manipulating both MDK and AP2A1. In MDK-overexpressing SW480 and RKO cells, simultaneous knockdown of AP2A1 significantly reduced the increased invasion, migration, and proliferation caused by MDK (Figure 6A,C,E,G). Conversely, in MDK-depleted SW620 and DLD1 cells, forced expression of AP2A1 effectively restored the impaired migratory and invasive capabilities (Figure 6B,D,F,H). These findings identify AP2A1 as a key downstream mediator of MDK, playing a central role in driving the aggressive traits of CRC cells.

### 3.7. MDK Knockdown Reduced Tumorigenesis in CRC Cells In Vivo

**Figure 7:**
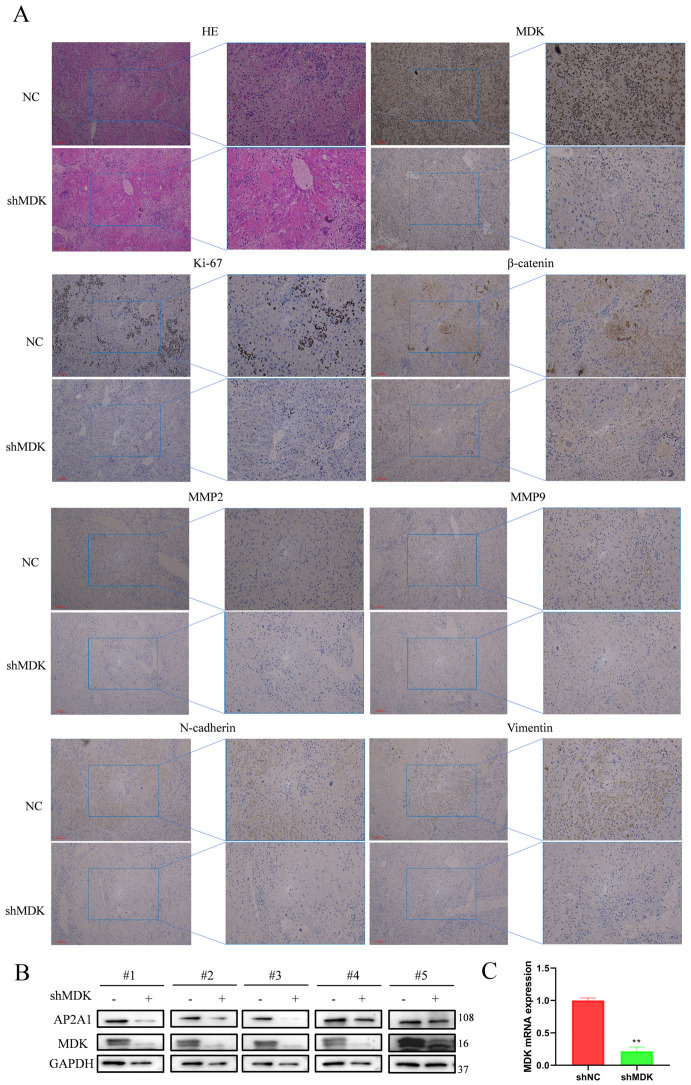
MDK knockdown inhibits tumor growth in vivo. (**A**) IHC analysis of xenograft tumors for the expression of Ki-67, β-catenin, MMP2, MMP9, N-cadherin, and Vimentin in the control (NC) and MDK-KD groups. Scale bars = 200 μm. All data are presented as mean ± SD. (**B**) Western blot analysis of MDK and AP2A1 expression in the tumors. (**C**) qRT-PCR analysis of MDK expression in the tumors. Data are shown as means ± SD. ** *p* < 0.01. The uncropped blots are shown in File S1.

To validate the tumorigenic role of MDK in vivo, we created subcutaneous xenografts in nude mice by injecting SW480 cells with stable MDK knockdown (shMDK) or control (shNC). Tumor volume and weight were significantly lower in the shMDK-derived xenografts compared to the shNC group (Supplementary Figure S5A–C). Consistent with our in vitro results, IHC analysis of the xenograft tumors showed that knocking down MDK decreased the expression of Ki-67 (proliferation), β-catenin, N-cadherin, Vimentin (EMT), and MMP2/MMP9 (invasion) (Figure 7A). Quantitative analysis confirmed these findings, with significantly lower H-scores (for MDK, MMP2, MMP9, N-cadherin, and Vimentin) and reduced percentages of Ki-67 and β-catenin positive cells in the shMDK group compared to controls (Supplementary Figure S5D–J). Western blot and qRT-PCR analyses verified the efficiency of MDK knockdown and showed a corresponding decrease in its downstream target, AP2A1, at both the protein and mRNA levels in the shMDK group (Figure 7B,C).

### 3.8. PI3K Contributes to MDK-Mediated Induction of AP2A1 Expression and Cell Invasion in CRC

**Figure 8:**
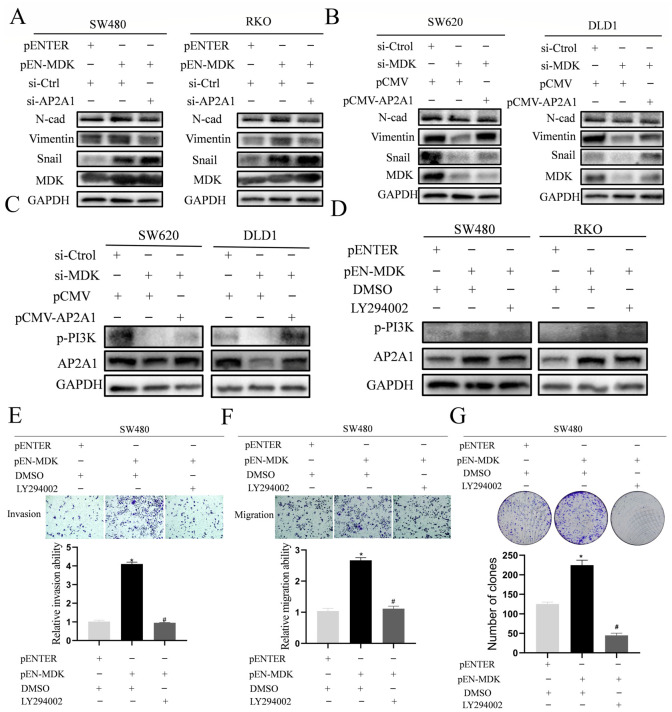
MDK-regulated AP2A1 expression and cell invasion depend on the activation of PI3K/AKT signaling. The expression of EMT markers (**A**,**B**) and key components of the PI3K/AKT pathway (**C**) was examined in CRC cells after co-transfection to manipulate MDK and AP2A1 levels. (**D**) The role of PI3K/AKT signaling was confirmed using the inhibitor LY294002. SW480 and RKO cells were pre-treated with LY294002 48 h post-transfection, then assessed for AP2A1 expression. Band intensities were quantified and normalized to GAPDH. (**E**–**G**) Functional effects of pathway inhibition: After LY294002 pre-treatment, the invasion, migration, and growth of SW480 cells were evaluated. (**E**,**F**): ×100 magnification; scale bar not shown. (**G**): ×20 magnification; scale bar not shown. * *p* < 0.05 vs. control; # *p* < 0.05 vs. MDK-overexpressing cells (Student’s *t*-test). The uncropped blots are shown in File S1.

We then hypothesized that AP2A1 mediates the pro-EMT role of MDK. AP2A1 knockdown blocked MDK-induced EMT (Figure 8A), and its overexpression reversed the EMT suppression caused by MDK depletion (Figure 8B). Considering the crucial role of the PI3K/AKT pathway, our findings suggest that re-expressing AP2A1 restored PI3K phosphorylation, although this effect was weaker in SW620 cells (Figure 8C). Importantly, the PI3K inhibitor LY294002 in MDK-overexpressing SW480 and RKO cells significantly reduced AP2A1 expression (Figure 8D) and the induction of cell invasion, migration, and growth (Figure 8E–G) by MDK, revealing a critical positive feedback loop. This describes a coherent MDK/PI3K/AP2A1 signaling pathway that promotes CRC malignancy.

## 4. Discussion

**Figure 9:**
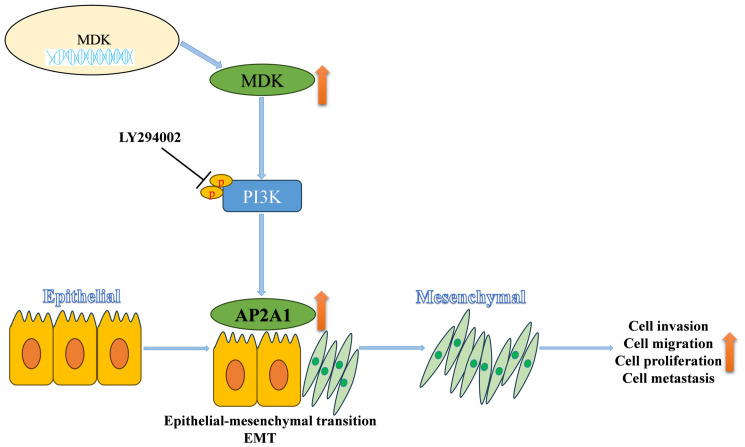
Schematic illustration of the mechanism by which MDK promotes EMT progression and induces AP2A1 levels in CRC cells.

This study shows that MDK is significantly increased in CRC tissues and cells compared to normal controls. Additionally, bioinformatics analysis reveals that high MDK levels are linked to poor prognosis in CRC, as confirmed by combined analysis of GEO and TCGA cohorts. Overexpression of MDK was found to enhance CRC cell invasion, migration, proliferation, and metastasis, supported by various assays including transwell, wound healing, CCK-8, EdU, colony formation, and a tail vein injection mouse model. These functional results reinforce the clinical link between elevated MDK expression and worse patient survival. Similarly, we found that MDK and AP2A1 are positively correlated in tissue samples. Mechanistically, MDK activates the PI3K/AKT pathway, which increases AP2A1 expression and promotes EMT in CRC (Figure 9). Further studies show that AP2A1 functions as a downstream regulator of the PI3K/AKT pathway driven by MDK in CRC invasion. Developing small-molecule inhibitors targeting MDK could provide a new therapeutic strategy for CRC.

The initiation of EMT is mainly controlled by key transcription factors, especially the SNAIL (SNAIL1, SNAIL2) [17], TWIST (TWIST1, TWIST2) [18] and ZEB (ZEB1, ZEB2) families [19]. These EMT-TFs can suppress the expression of epithelial marker genes like CDH1 [20]. EMT-TFs activate genes associated with mesenchymal traits, such as waveform proteins and N-cadherin, either directly or indirectly [21,22]. Analysis of CRC-related clinical data indicates that higher levels of MDK/SDC4 are linked to decreased OS in CRC patients [5,23], but the underlying mechanism is still unclear. Examining EMT’s role in CRC and targeted therapies could help manage cancer metastasis.

MDK is a soluble protein found in the blood, urine, and cerebrospinal fluid of healthy individuals [24]. It is present in various mature tissues and organs, including the gastrointestinal system, kidneys, lymphocytes, and macrophages [25]. MDK plays a role in regulating diverse physiological functions, such as development, reproduction, repair, innate immunity [26], blood pressure regulation [27] and angiogenesis [28]. It can serve as a valuable tumor marker in certain disease states, including hepatitis [29], autoimmune diseases [5] and is highly expressed in at least 20 types of cancer [8,9]. MDK interacts with SDC4 as a receptor to communicate with regulatory T cells (Tregs) within the tumor microenvironment, helping tumors establish an immunosuppressive environment and promoting early carcinogenesis [6]. It binds to Syndecan-1, activating the PI3K/AKT and p38 MAPK signaling pathways, which promote cancer cell proliferation, migration, invasion, and lymphangiogenesis, collectively driving lymph node metastasis [30]. As a classic receptor for MDK, Syndecan-1 mediates the activation of multiple downstream oncogenic signaling pathways, such as PI3K/AKT and MAPK, influencing cell survival, proliferation, and migration [31]. Intracellularly, MDK binds to the LKB1-STRAD-Mo25 complex, disrupting its structure and inhibiting AMPK kinase activity. This removes the growth restriction imposed by AMPK, thereby promoting cancer cell proliferation [32]. However, the relationship between MDK and EMT in CRC remains unclear. This study analyzed MDK expression in the TCGA and GEO databases, revealing elevated levels in CRC tissues compared to normal tissues. We confirmed that MDK is associated with poor prognosis in CRC. Our findings show that MDK overexpression enhances CRC cell migration, invasion, and proliferation. Additionally, we provide evidence that MDK can trigger EMT in CRC cells, although its underlying mechanism remains undefined.

Given that the PI3K/AKT pathway plays a crucial role in cell proliferation, survival, and apoptosis prevention, it is notable that its activation has been observed in various tumors following MDK treatment [33]. By activating the PI3K/AKT and MAPK signaling pathways, MDK stimulates proliferation across different cell types, including neuroblastoma (IGR-N91 and SH-SY5Y) [34], glioblastoma (U87MG and A172) [35] and endothelial cells [36], and more recently demonstrated in thyroid cancer [37] and breast cancer [38,39]. Emerging evidence also implicates MDK in regulating the PI3K/AKT axis to promote metastasis and EMT across multiple cancer types [40]. The glycolytic enzyme ENO1 directly activates PI3K/AKT in a concentration-dependent manner by producing ATP and lactate, forming a positive feedback loop [36,41]. PI3K is a family of kinases that serve as core components in cellular signaling pathways, and AKT, a serine/threonine kinase, is a key downstream effector regulating various biological processes by directly phosphorylating transcription factors like NF-κB and mTOR [42]. AKT promotes proliferation by phosphorylating the Thr258 site of the Prohibitin (PHB) protein, which drives its translocation to mitochondria [43]. As shown in our study, MDK activates PI3K and AKT, leading to increased phosphorylation of these proteins. We further investigated potential molecular mechanisms using bioinformatics and RNA-seq analysis, confirming MDK’s involvement in the PI3K/AKT pathway. Recent studies indicate that the AP2A1 protein maintains cellular structural integrity by regulating cell–matrix adhesion [44], suggesting that AP2A1 may act as a downstream target gene of MDK. Further analyses and experiments demonstrated that AP2A1 functions as an oncogene in CRC and rescues the effects of MDK on CRC cells. These findings imply that targeting MDK could be a promising therapeutic strategy to disrupt the oncogenic PI3K/AKT pathway and inhibit malignancy in CRC.

We acknowledge several limitations in this study. Our clinical sample size was modest and derived from a single center, warranting validation in larger independent cohorts. The in vitro experiments were confined to a limited number of CRC cell lines, and the in vivo xenograft models lack a fully functional immune system. Additionally, while we demonstrate that MDK regulates AP2A1 via the PI3K/AKT pathway, the detailed molecular mechanism underlying this regulation requires further investigation.

## 5. Conclusions

Our study demonstrated that MDK expression is higher in CRC tissues and cells compared to normal counterparts. MDK is associated with poor outcomes in CRC. Our data show that MDK promotes invasion, proliferation, migration, and EMT of CRC cells. Mechanistically, MDK activates the PI3K/AKT pathway to induce AP2A1 expression and EMT. Therefore, MDK appears to be a promising biomarker for the diagnosis and prognosis of CRC.

## Figures and Tables

**Table 1:** Clinicopathological characteristics in relation to MDK expression status in TCGA cohort.

Characteristics	TCGA Cohort (N = 597)	MDK Expression		χ^2^	*p* Value
		High N = 393 (%)	Low N = 204 (%)		
Age					
<65 y	244	157 (64.3)	87 (35.7)	0.404	0.525
≥65 y	353	236 (66.9)	117 (33.1)		
Gender					
male	321	210 (65.4)	111 (34.6)	0.052	0.820
female	276	183 (66.3)	93 (33.7)		
Race					
ASIAN	11	6 (54.5)	5 (45.5)		
BLACK or AFRICAN AMERICAN	58	34 (58.6)	24 (41.4)		
WHITE	210	122 (58.1)	88 (41.9)	14.441	**0.006**
AMERICAN INDIAN or ALASKA NATIVE	1	1 (100)	0 (0)		
NA	317	230 (72.6)	87 (27.4)		
T					
T1–T2	87	56 (64.4)	31 (35.6)		
T3–T4	351	228 (65)	123 (35)	0.726	0.696
NA	159	109 (68.6)	50 (31.4)		
N					
N0 + N1	370	235 (62.5)	135 (37.5)		
N2 + N3	78	59 (75.6)	19 (24.4)	5.637	0.060
NX	159	109 (68.6)	50 (31.4)		
M					
M0	330	214 (64.8)	116 (35.2)		
M1	61	44 (72.1)	17 (27.9)	1.226	0.542
MX	206	135 (65.5)	71 (34.5)		
Pathologic Stage					
stage I–II	322	203 (63)	119 (37)	6.607	**0.037**
stage III–IV	263	185 (70.3)	78 (29.7)		
NA	12	5 (41.7)	7 (58.3)		
Status					
Alive	473	301 (63.6)	172 (36.4)	4.868	**0.027**
Dead	124	92 (71.2)	32 (25.8)		

Statistical significance was determined by Chi-square test or Fisher’s exact test. Bold values indicate statistical significance (*p* < 0.05).

**Table 2:** Univariate and multivariate analysis for overall survival (OS) in patients with CRC.

Characteristics	No			OS	
		Univariate Analysis		Multivariate Analysis	
		χ^2^	*p*	χ^2^	*p*
Age					
<65 y	244	1.815	**0.003**	1.784	**0.021**
≥65 y	353				
Gender					
male	321	0.956	0.802		
female	276				
Race					
ASIAN	11	0.926	0.324		
BLACK or AFRICAN AMERICAN	58				
WHITE	210				
AMERICAN INDIAN or ALASKA NATIVE	1				
NA	317				
T					
T1–T2	87	3.321	**0.004**	3.090	0.062
T3–T4	351				
NA	159				
N					
N0 + N1	370	3.466	<**0.001**	1.758	**0.035**
N2 + N3	78				
NX	159				
M					
M0	330	4.508	<**0.001**	2.589	<**0.001**
M1	61				
MX	12				
Pathologic Stage					
stage I–II	322	3.282	<**0.001**	1.664	0.103
stage III–IV	263				
NA	12				
MDK expression					
High	393	1.519	**0.042**	0.193	0.474
Low	204				

Statistical significance was determined by Chi-square test or Fisher’s exact test. Bold values indicate statistical significance (*p* < 0.05).

## Data Availability

Public data from TCGA (https://portal.gdc.cancer.gov/) and GEO (https://www.ncbi.nlm.nih.gov/geo/) databases were analyzed in this research. The data supporting the findings of this study can be obtained from the corresponding author upon reasonable request.

## Supplementary Materials

The following supporting information can be downloaded at: https://www.mdpi.com/article/10.3390/cancers18081311/s1, Figure S1: mRNA expression patterns of MDK in different types of human cancer; Figure S2: DNA methylation aberration of MDK in tumors; Figure S3: Overexpression of MDK indicates poor clinical outcome of colorectal cancer (CRC); Figure S4: MDK promoted CRC cell proliferation and migration; Figure S5: MDK knockdown inhibits tumor growth in vivo and quantification of immunohistochemical staining; Table S1: Primers used in the study; Table S2: Details of antibodies used in this study; Table S3: The siRNA sequences used in this study; Table S4: Clinicopathological characteristics in relation to MDK expression status in SGH cohort; Table S5: Univariate and multivariate analysis for overall survival (OS) in patients with CRC; Table S6: Clinicopathological characteristics in relation to MDK expression status in TCGA cohort; Table S7: Full list of DEGs. File S1: Full uncropped Gels and Blots images.

## Abbreviations

The following abbreviations are used in this manuscript:

CRC	Colorectal cancer
MDK	Midkine
EMT	Epithelial–mesenchymal transition
CAFs	Cancer-associated fibroblasts
TMA	The tissue microarrays (TMA)
WB	Western blotting
IHC	Immunohistochemistry
CCK-8	Cell Counting Kit-8
qRT-PCR	Quantitative reverse transcription polymerase chain reaction
TCGA	The Cancer Genome Atlas
GEO	Gene Expression Omnibus
OS	Overall Survival
