# Supplementary material for: MDK Activates the PI3K/AKT Axis to Induce AP2A1 Expression and Epithelial–Mesenchymal Transition in Colorectal Cancer
Source: Cancers (Basel). 2026 Apr 21;18(8):1311. doi: 10.3390/cancers18081311 (PMC13114282; doi:10.3390/cancers18081311)

Figure 2B, 2F

Original cropped image of the single membrane

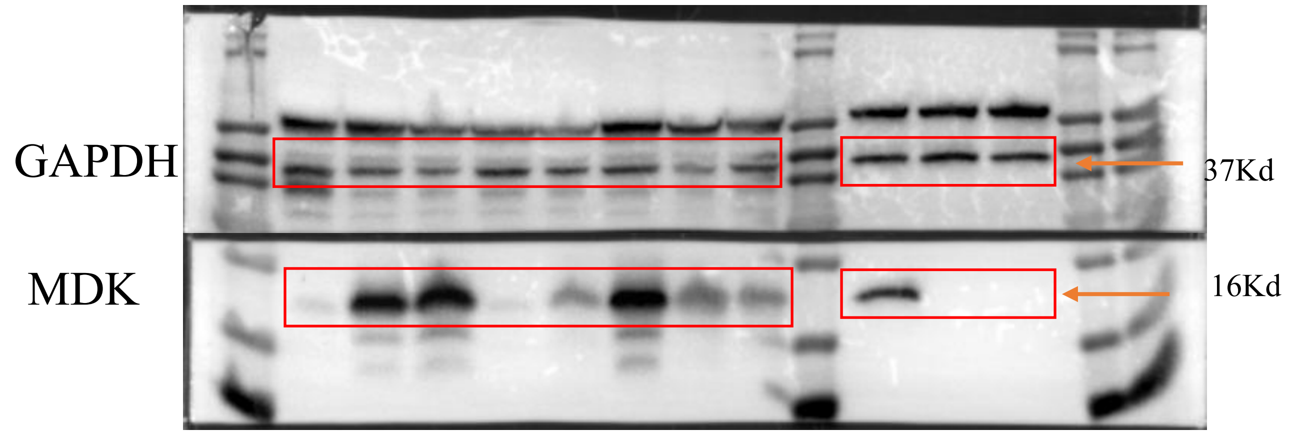

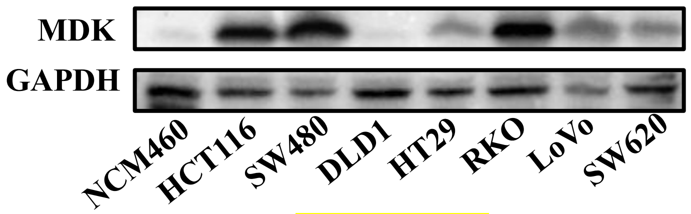

Figure 2B

SW480

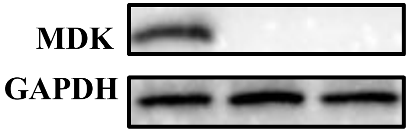

Figure 2F

Original cropped image of the single membrane

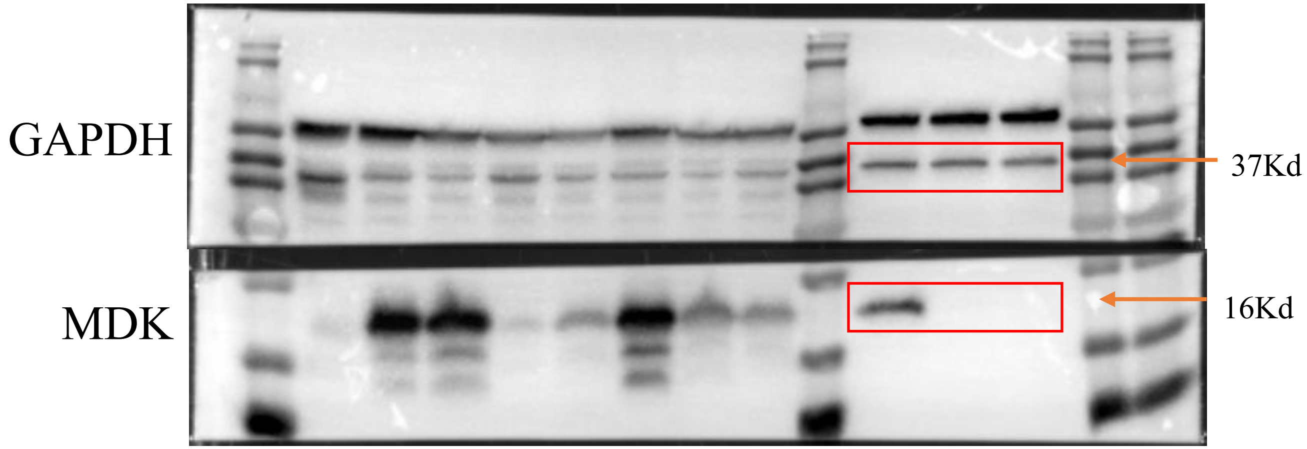

RKO

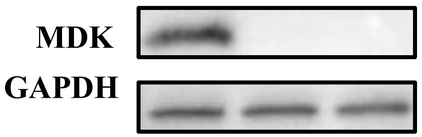

Figure 2F

Figure 2C

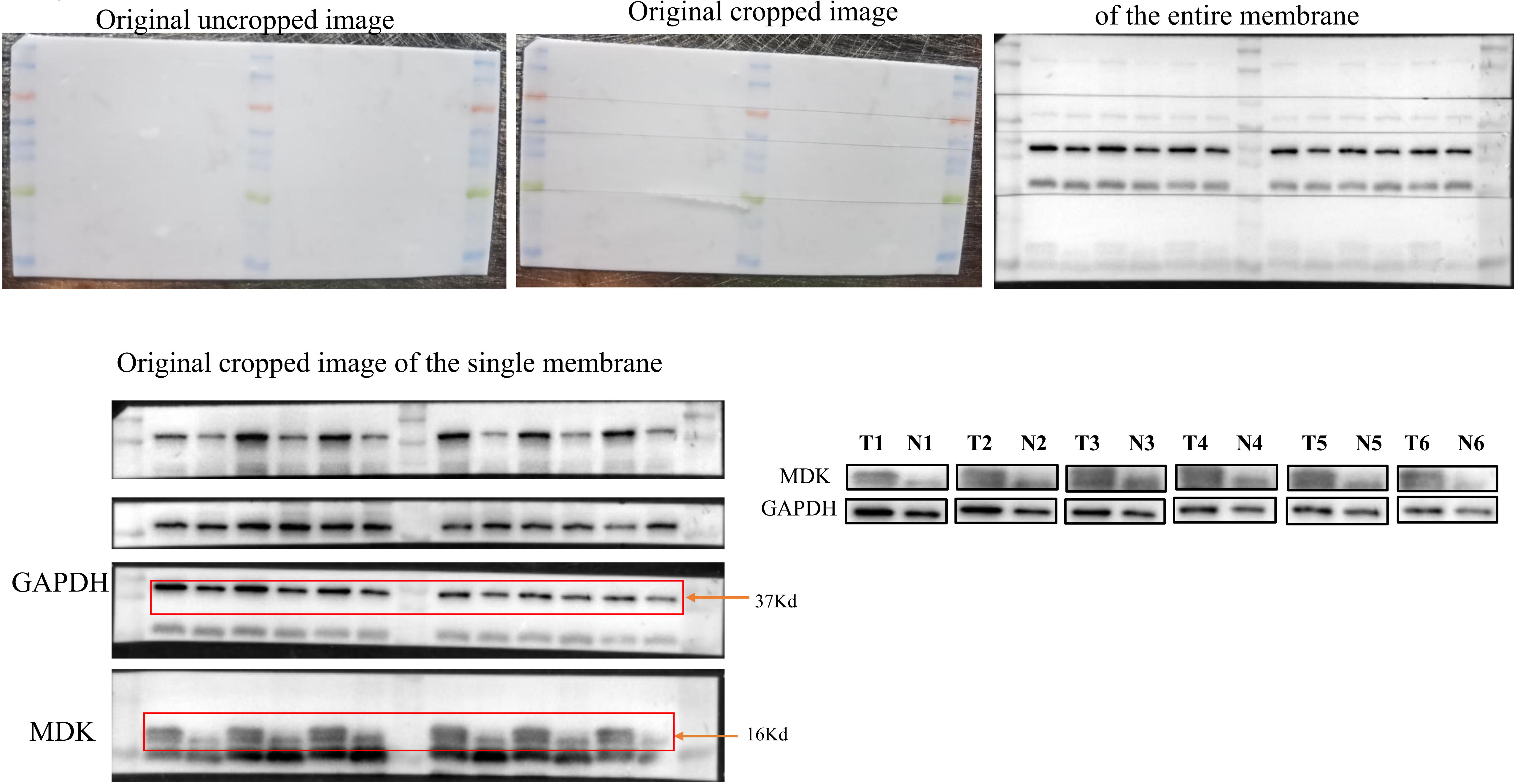

Original uncropped image
Original cropped image
Original cropped image of the entire membrane
Original cropped image of the single membrane
GAPDH
37Kd
MDK
16Kd
T1 N1 T2 N2 T3 N3 T4 N4 T5 N5 T6 N6
MDK
GAPDH

Figure 2C

Original uncropped image

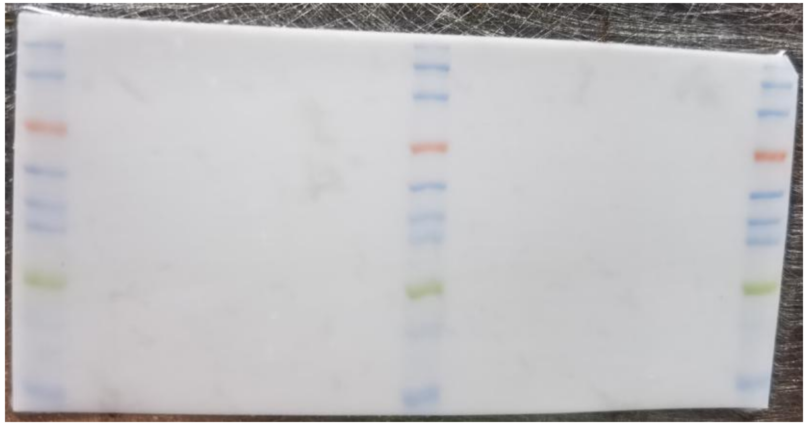

Original cropped image

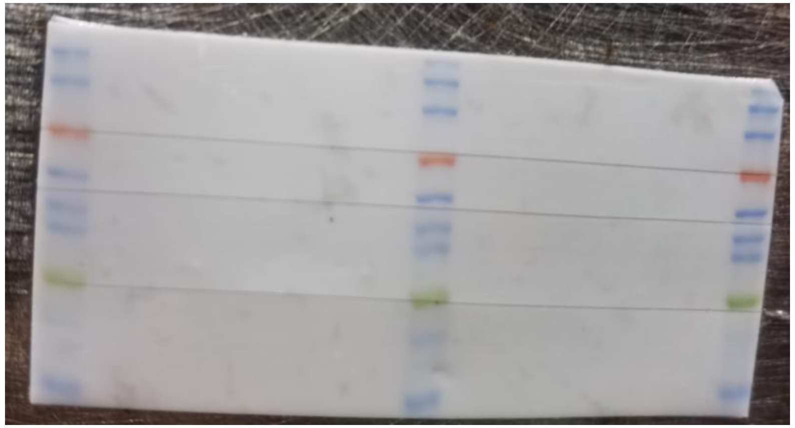

Original cropped image
of the entire membrane

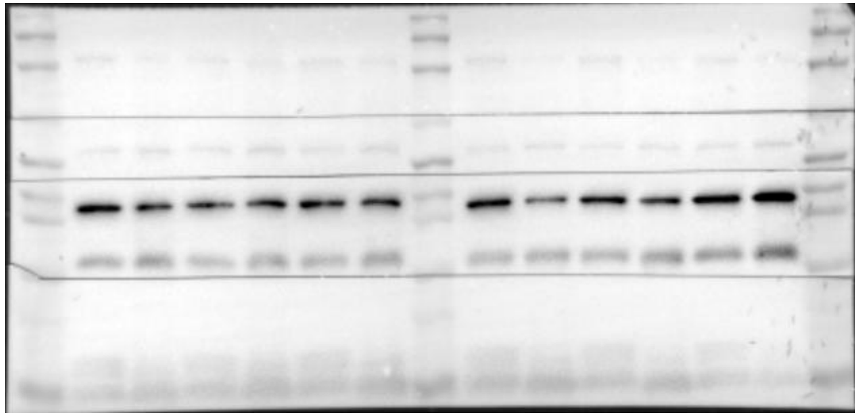

Original cropped image of the single membrane

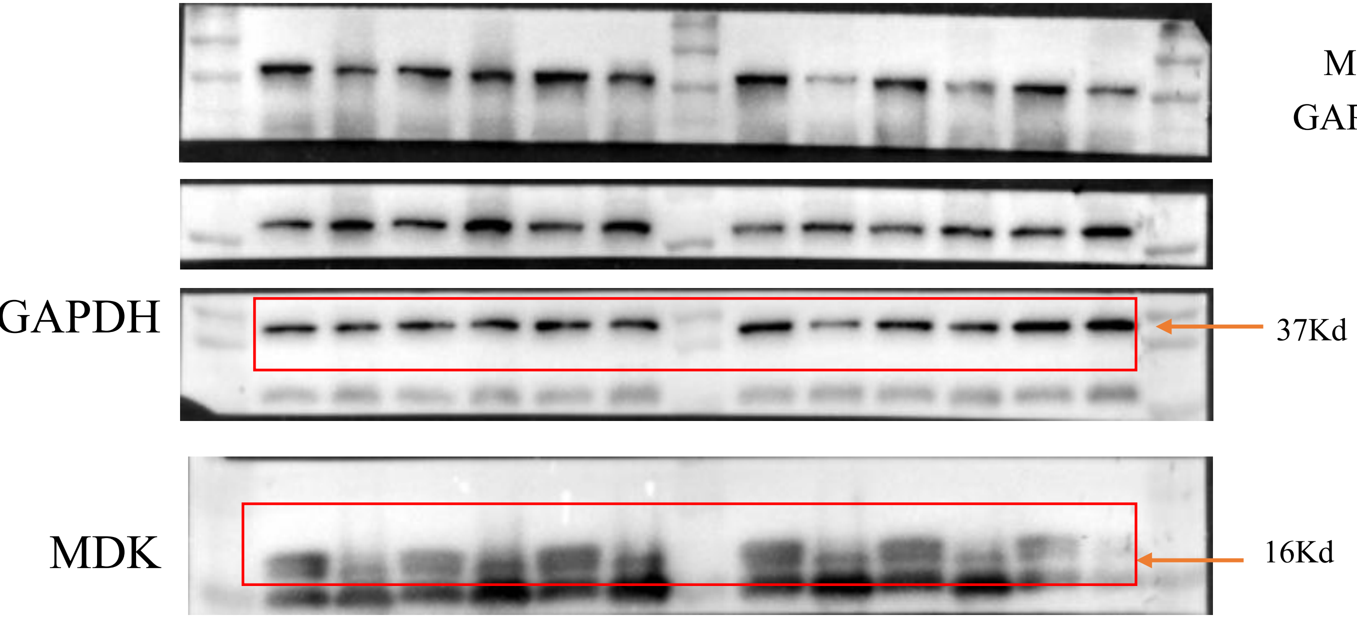

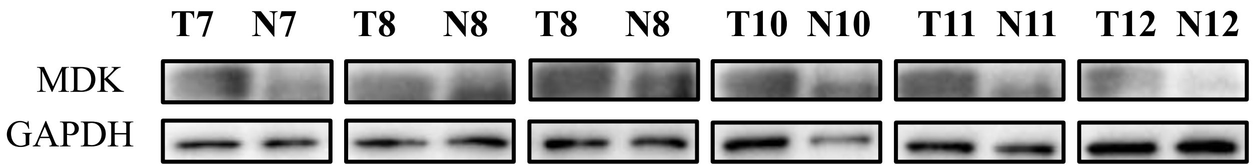

Figure 2G

Original cropped image of the entire membrane

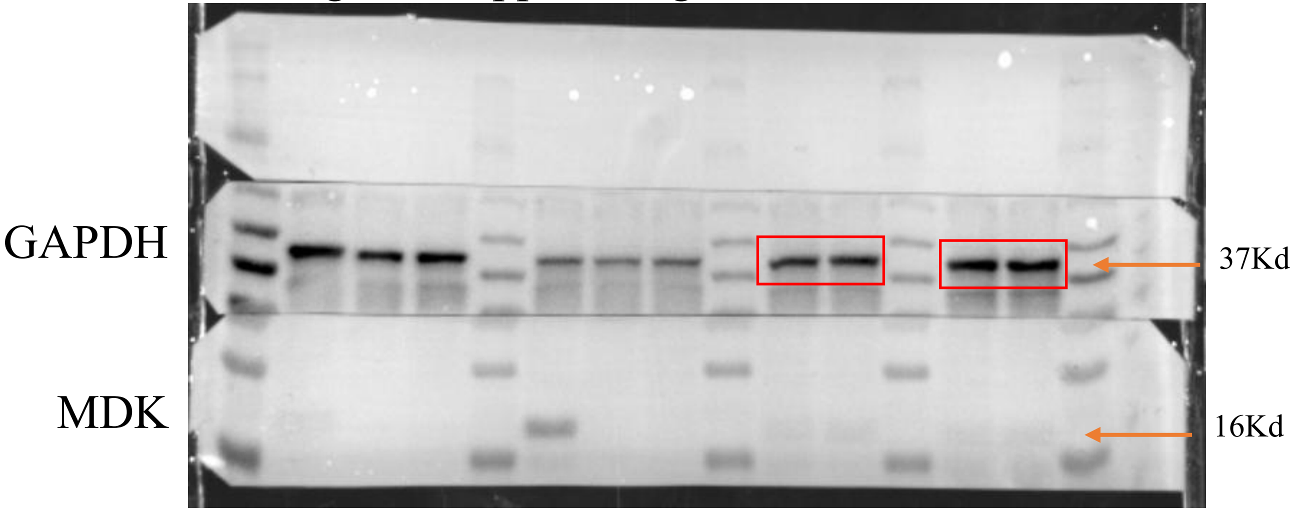

Original cropped image of the single membrane

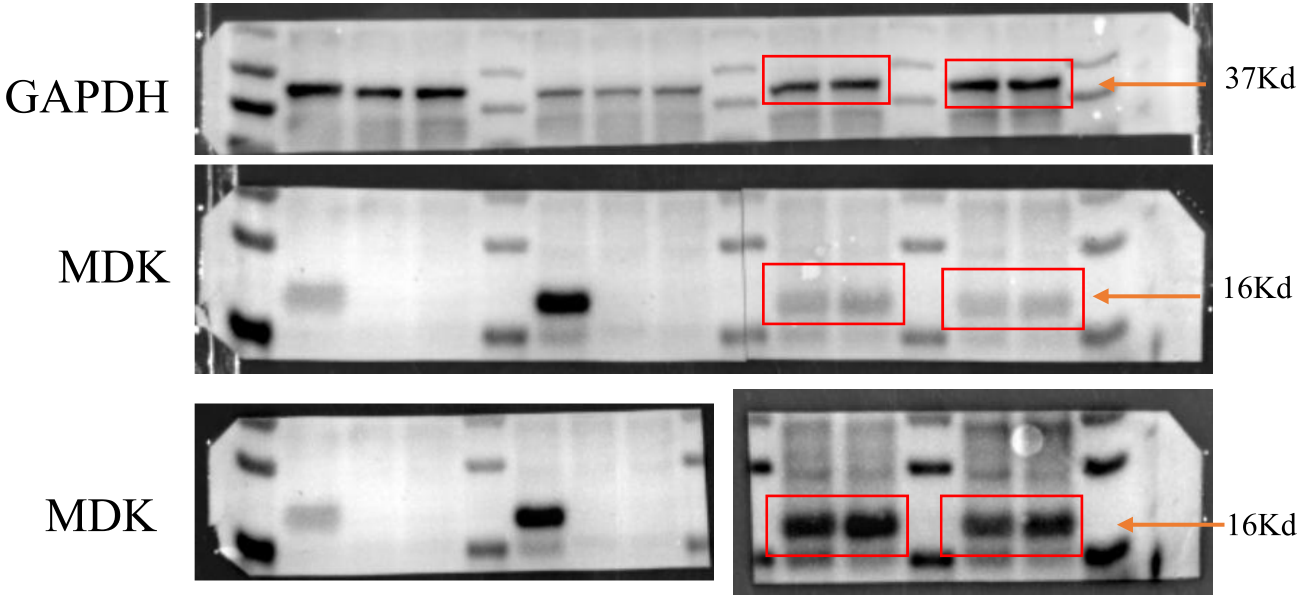

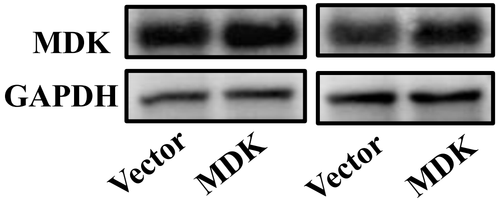

Figure 2G

Figure 4A-MMP2

Original uncropped image

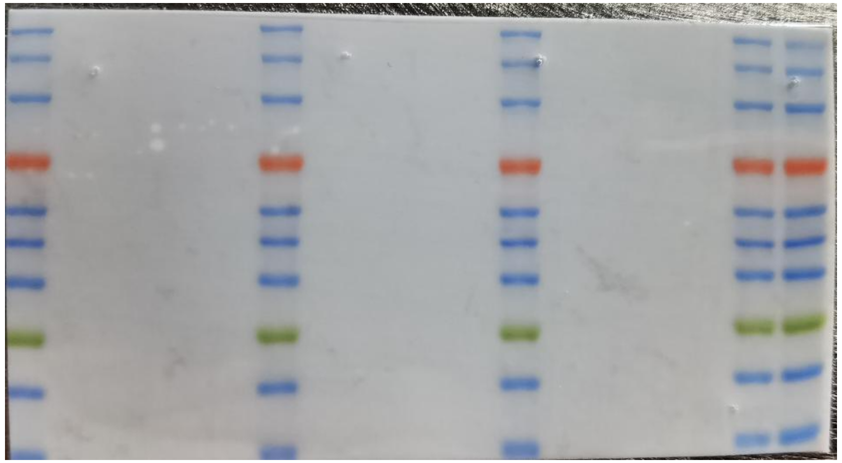

Original cropped image

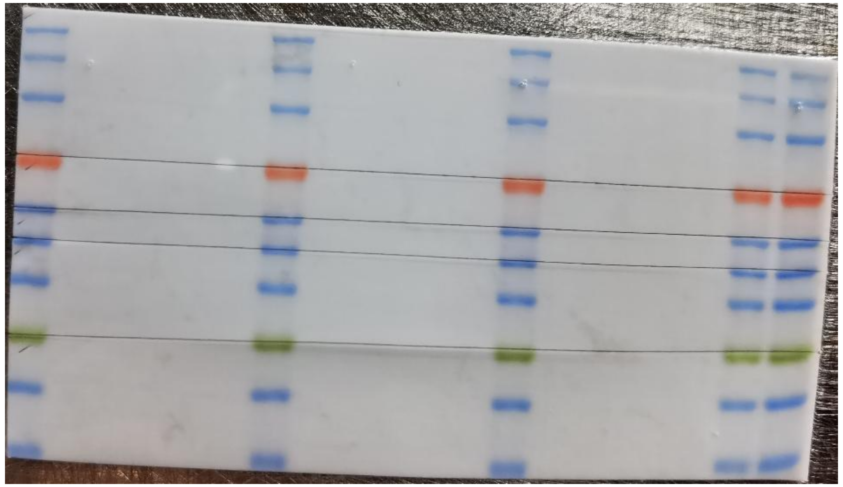

Original cropped image
of the entire membrane

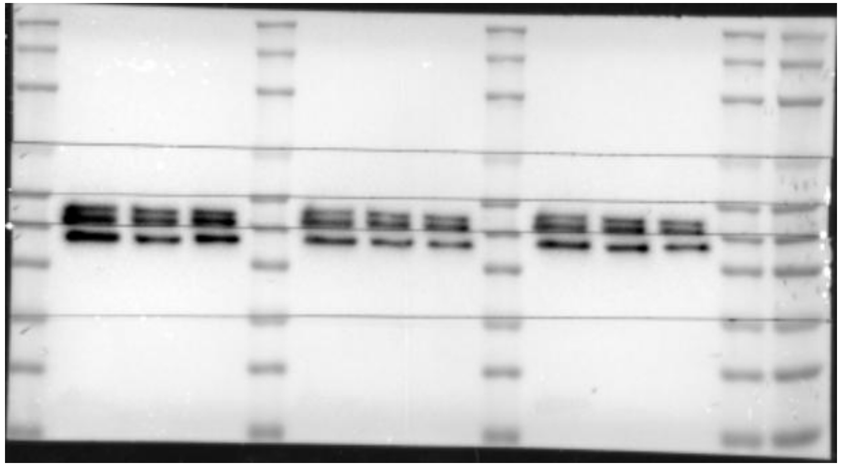

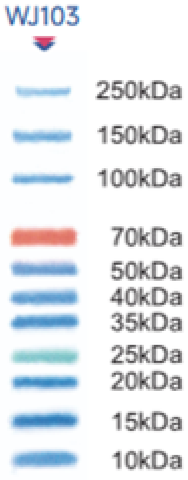

Original cropped image of the single membrane

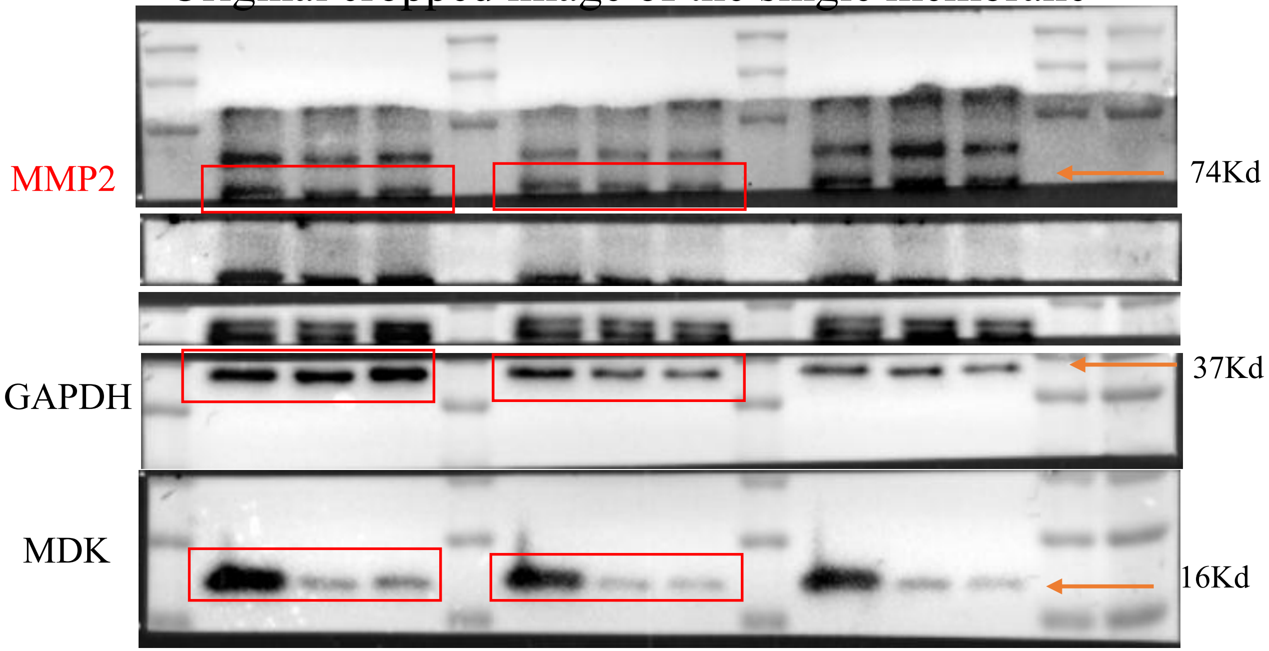

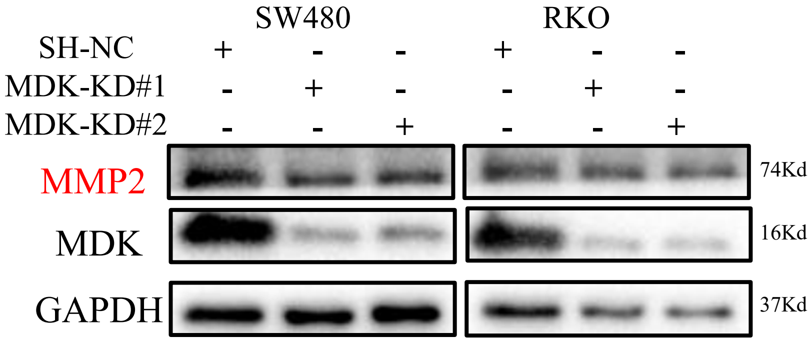

Figure 4A-MMP2

Figure 4A-MMP9

Original uncropped image

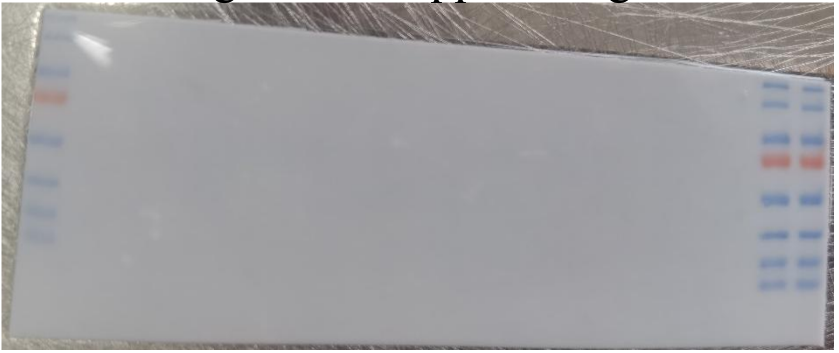

Original cropped image

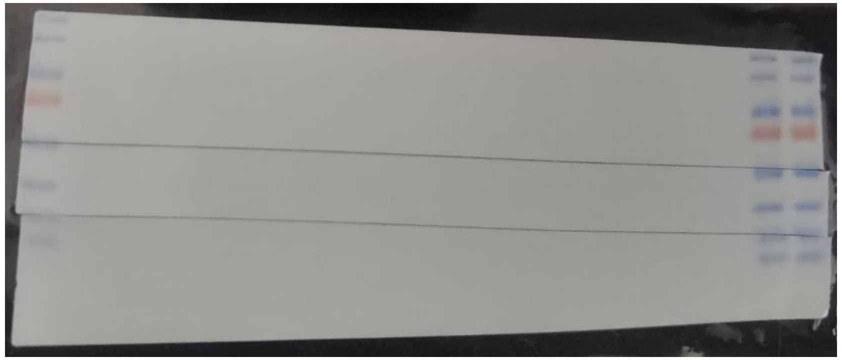

Original cropped image of the entire membrane

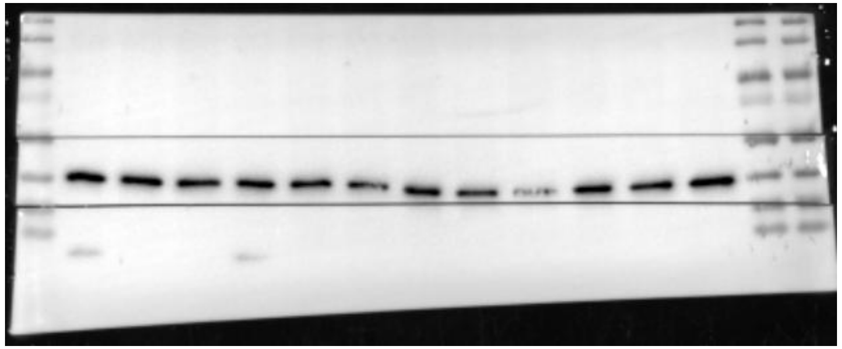

Original cropped image of the single membrane

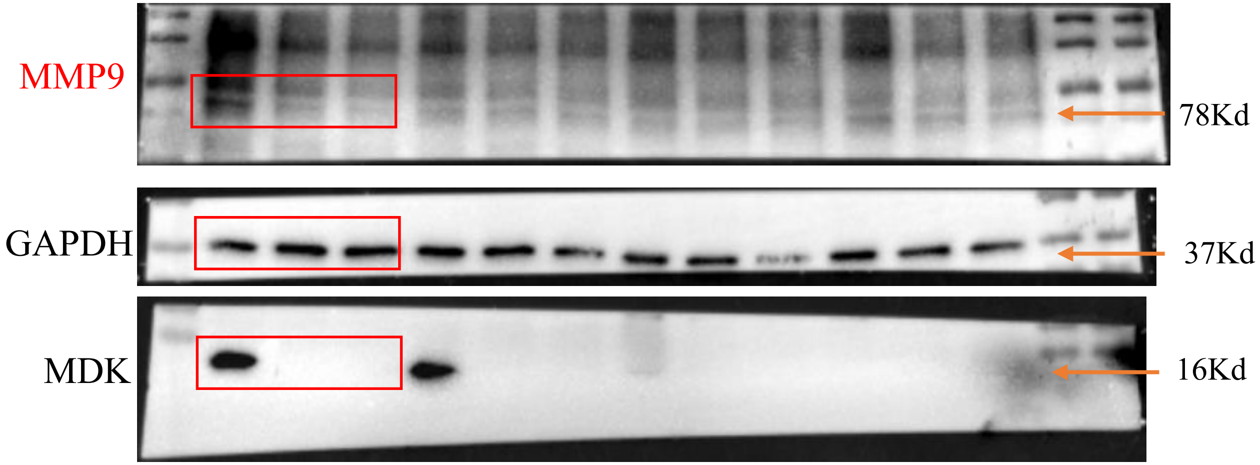

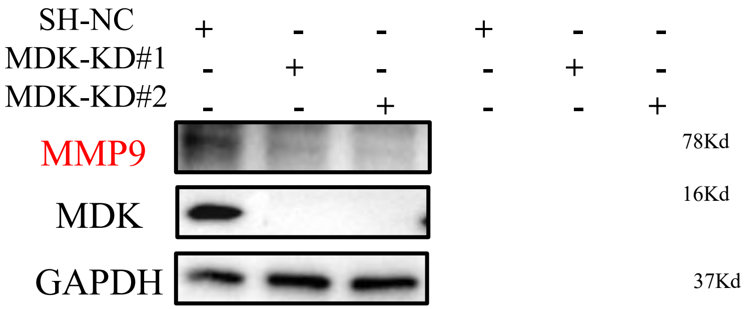

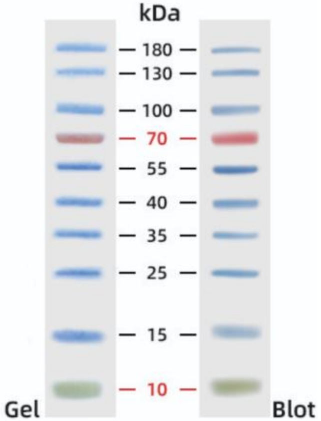

Figure 4A-MMP9

Figure 4A-MMP9

Original uncropped image

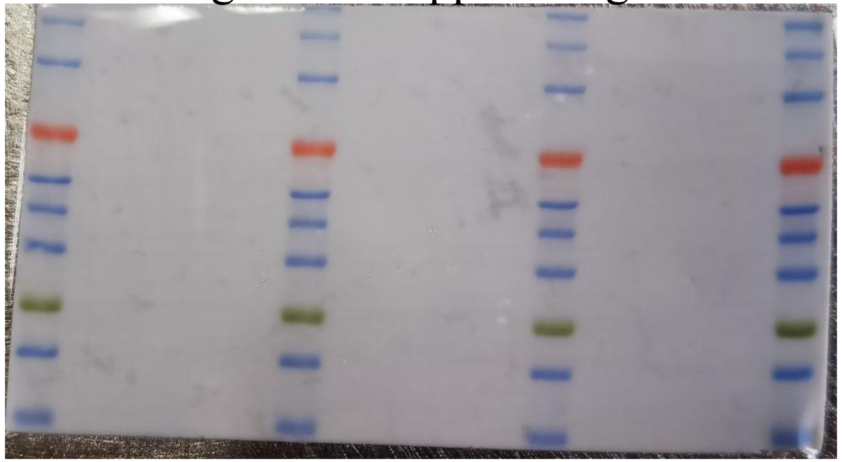

Original cropped image

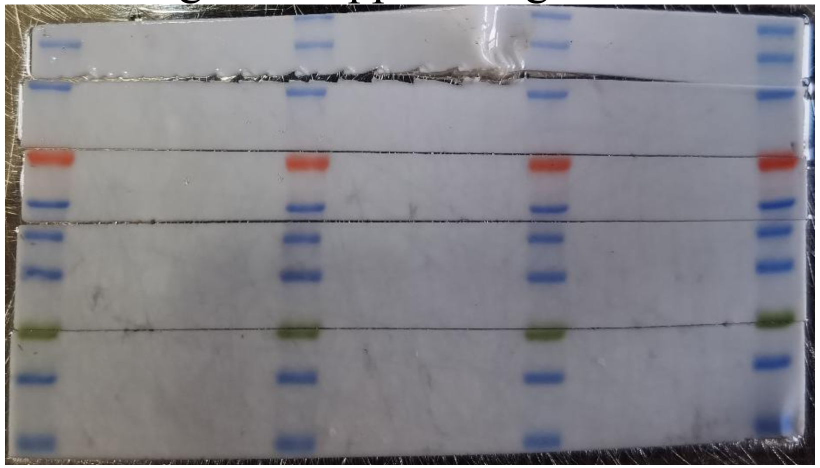

Original cropped image of the entire membrane

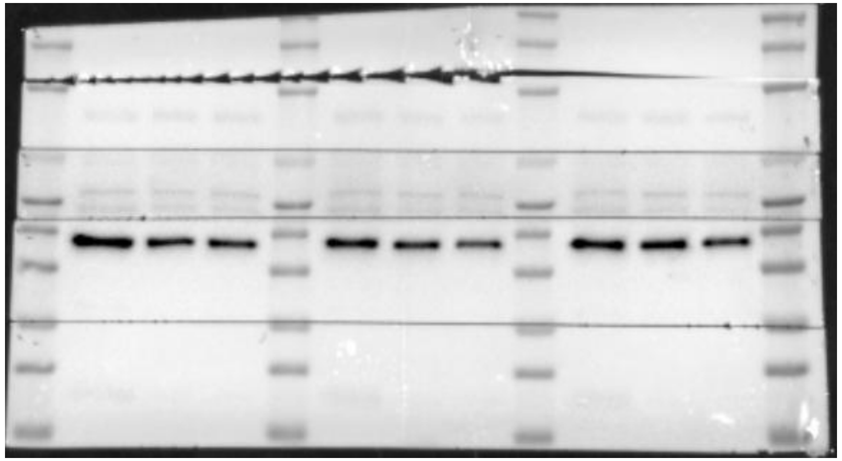

Original cropped image of the single membrane

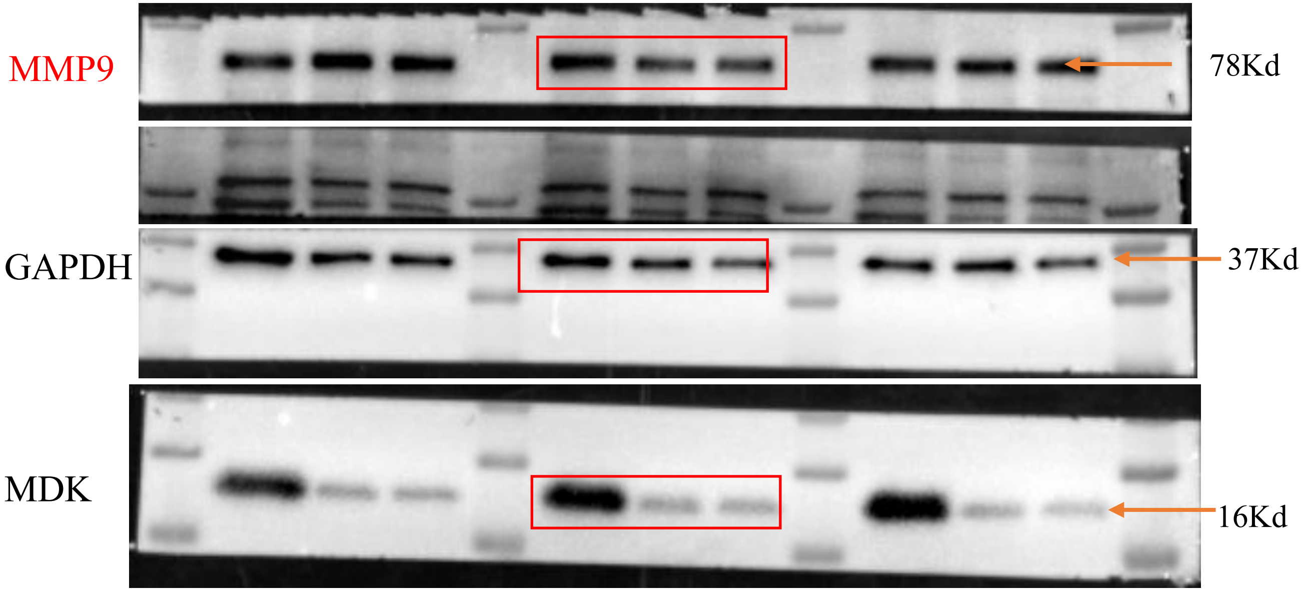

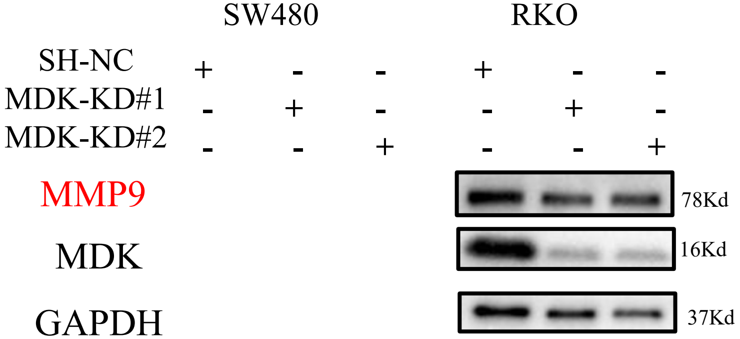

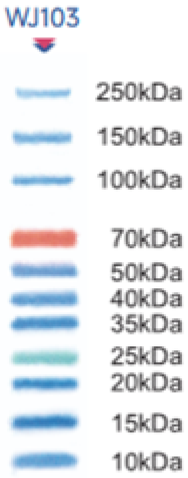

Figure 4A-MMP9

Figure 4A-Vimentin

Original uncropped image

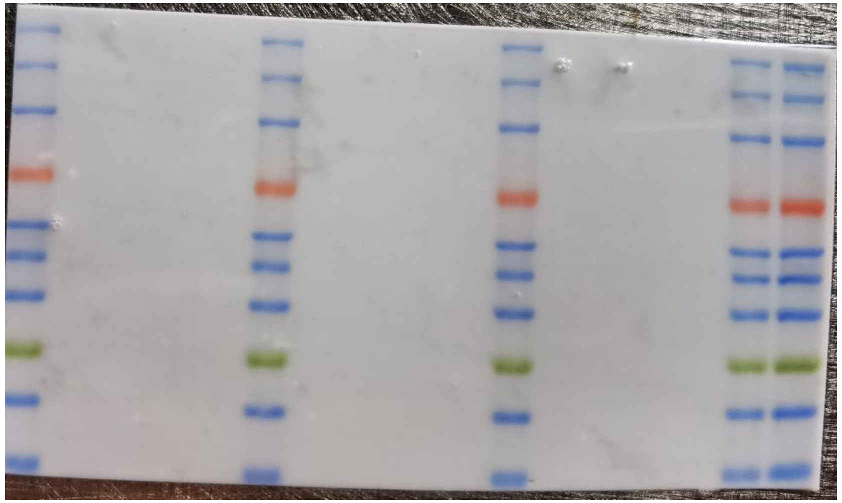

Original cropped image

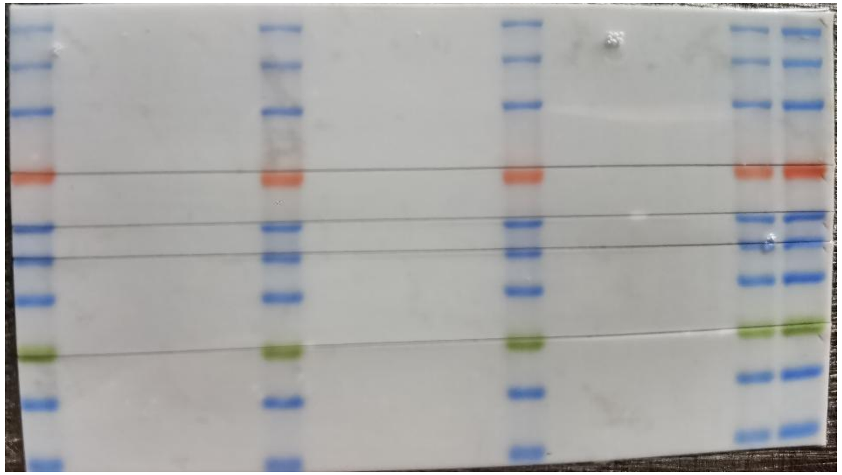

Original cropped image
of the entire membrane

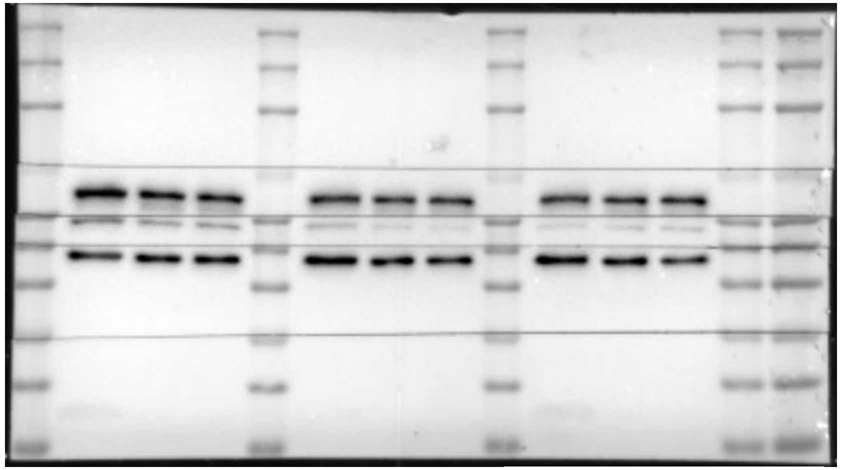

Original cropped image of the single membrane

Vimentin

GAPDH

MDK

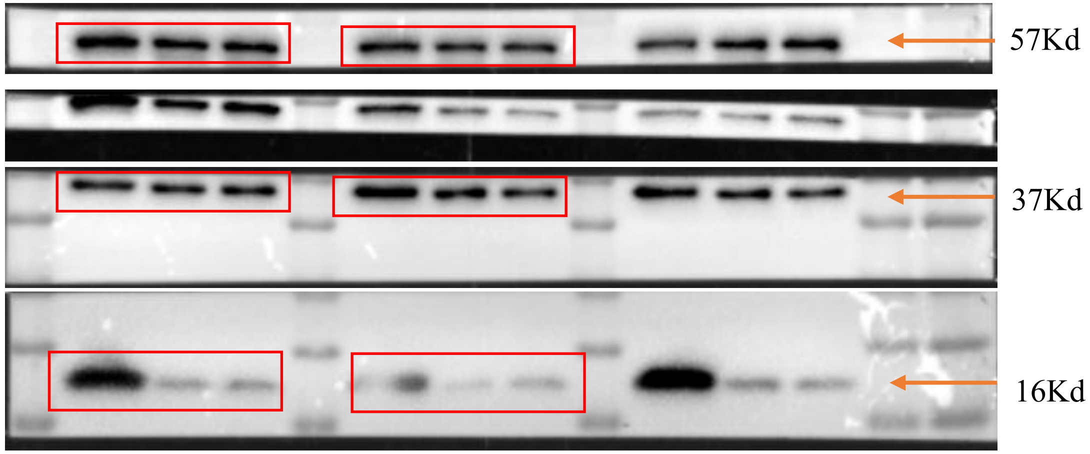

57Kd

37Kd

16Kd

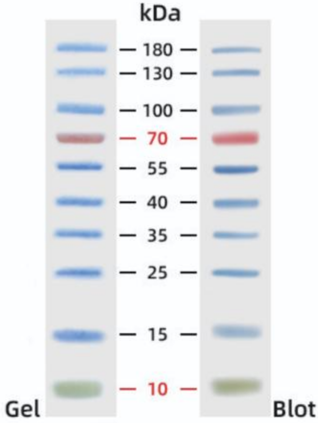

SW480 RKO

SH-NC + - - + - -
MDK-KD#1 - + - - + -
MDK-KD#2 - - + - - +

Vimentin

MDK

GAPDH

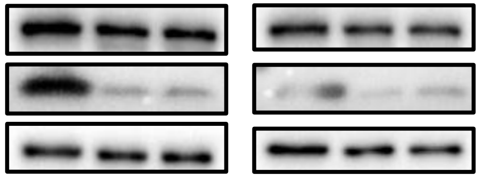

57Kd

16Kd

37Kd

Figure 4- Vimentin

Figure 4A- N-cad

Original uncropped image

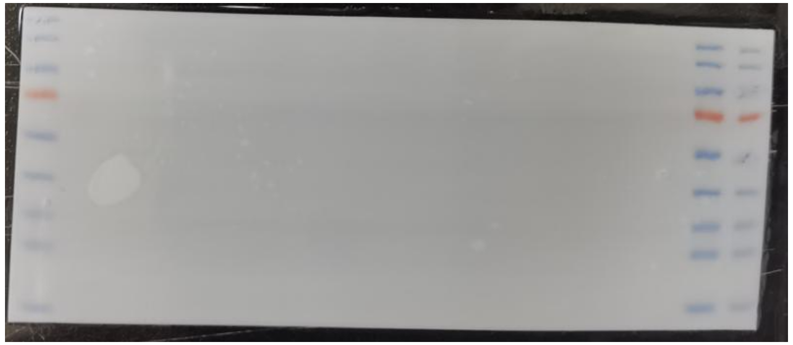

Original cropped image

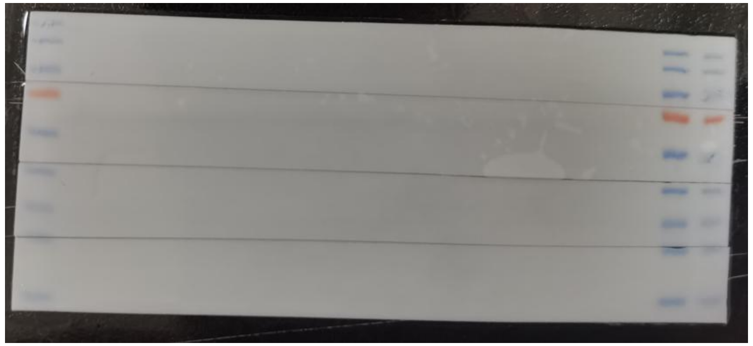

Original cropped image of the entire membrane

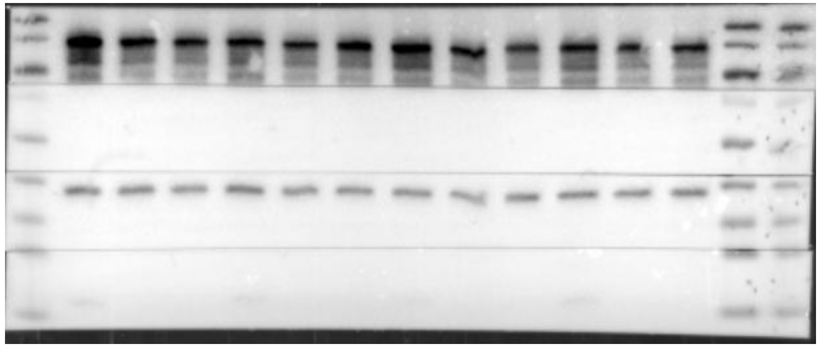

Original cropped image of the single membrane

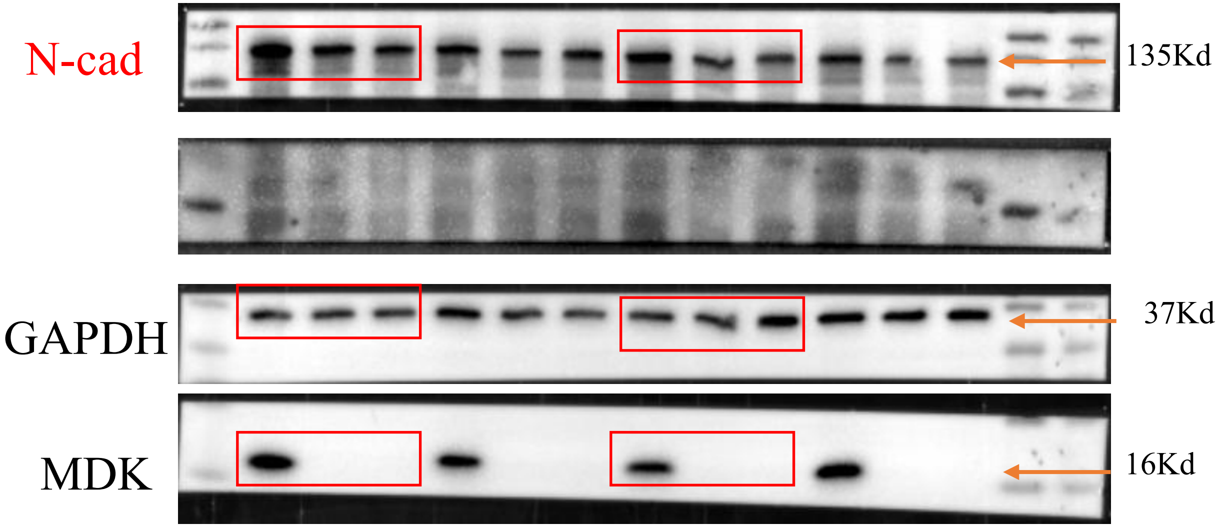

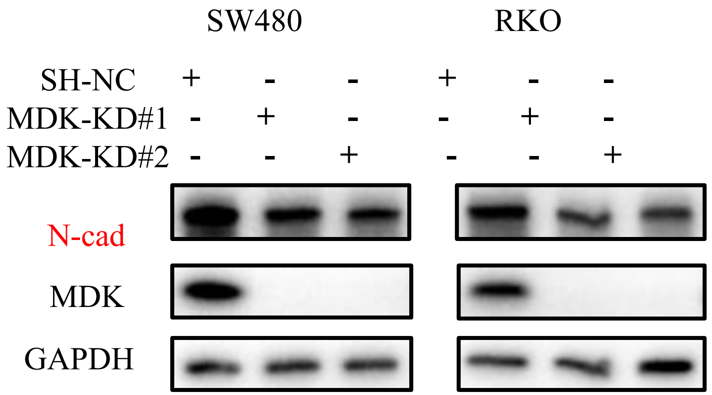

Figure 4- N-cad

Figure 4A- β-catenin, MDK, GAPDH

Original uncropped image

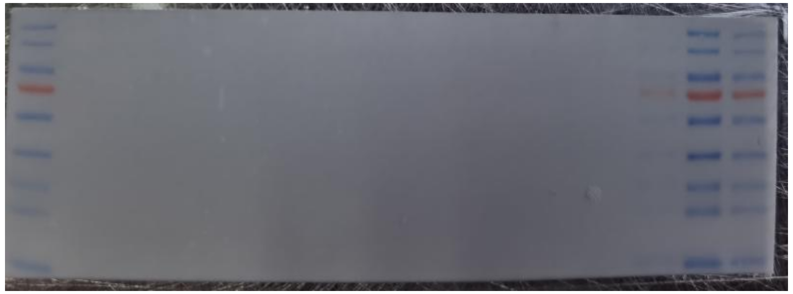

Original cropped image

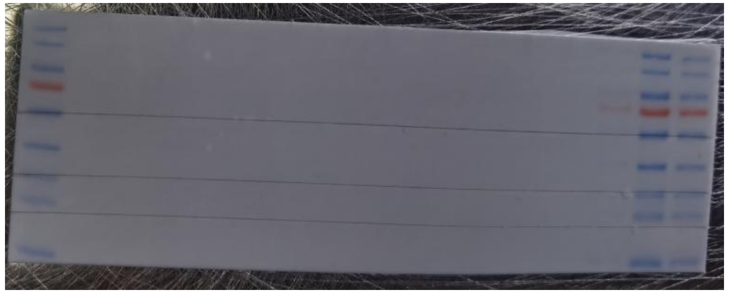

Original cropped image of the entire membrane

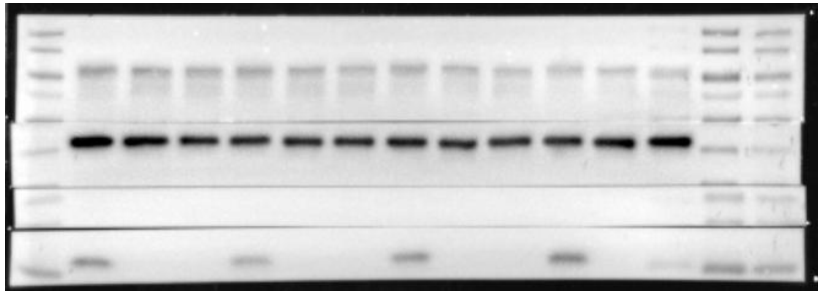

Original cropped image of the single membrane

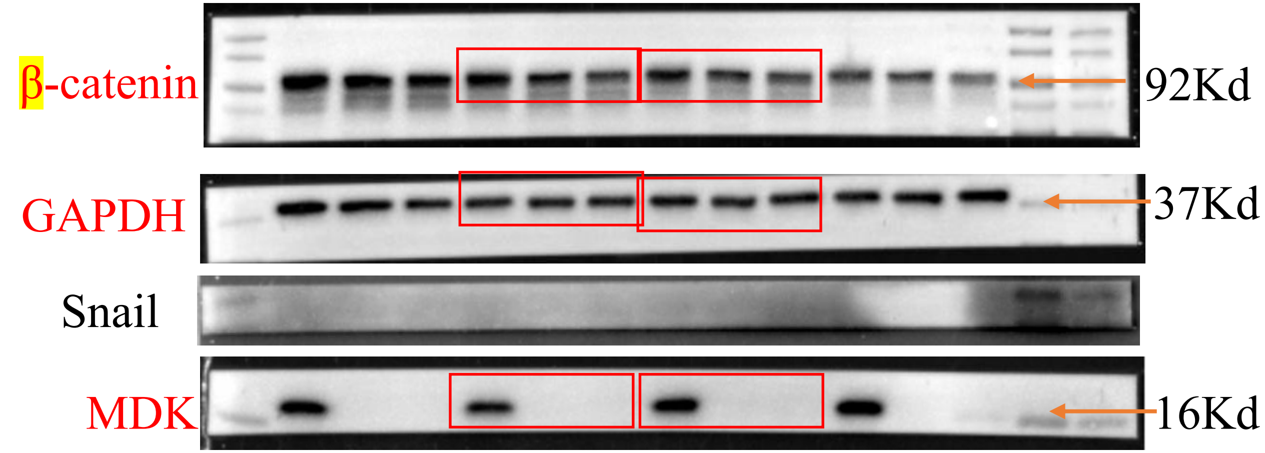

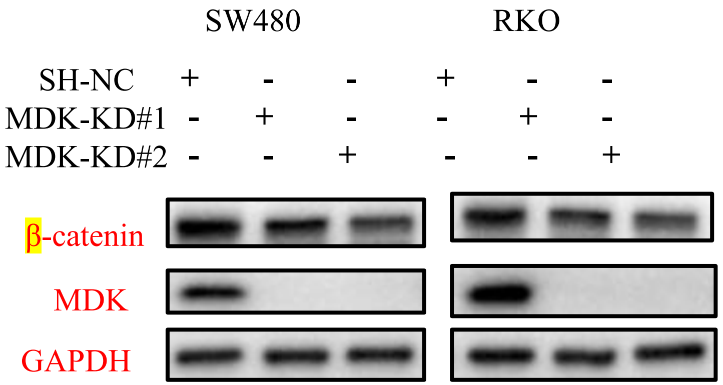

Figure 4A- β-catenin,MDK,GAPDH

Figure 4A- Snail

Original cropped image of the entire membrane

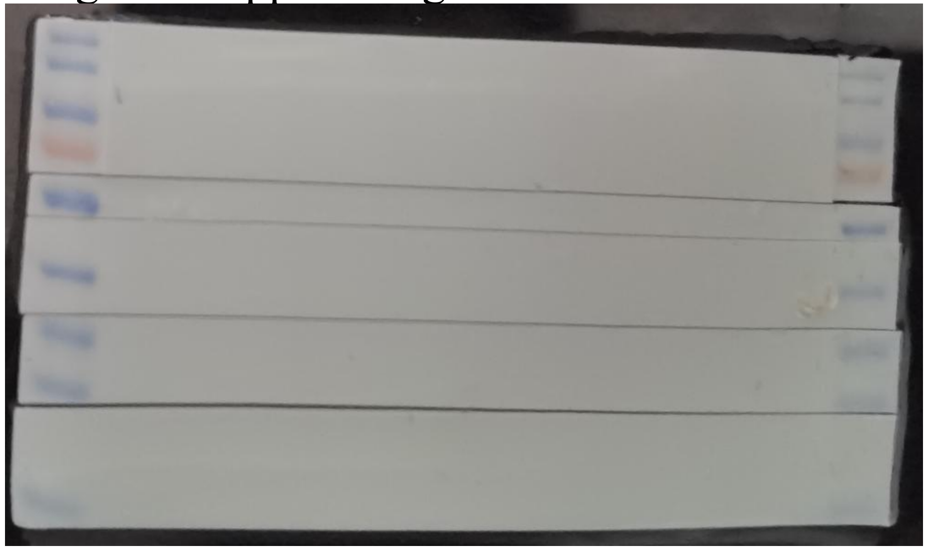

Original cropped image of the single membrane

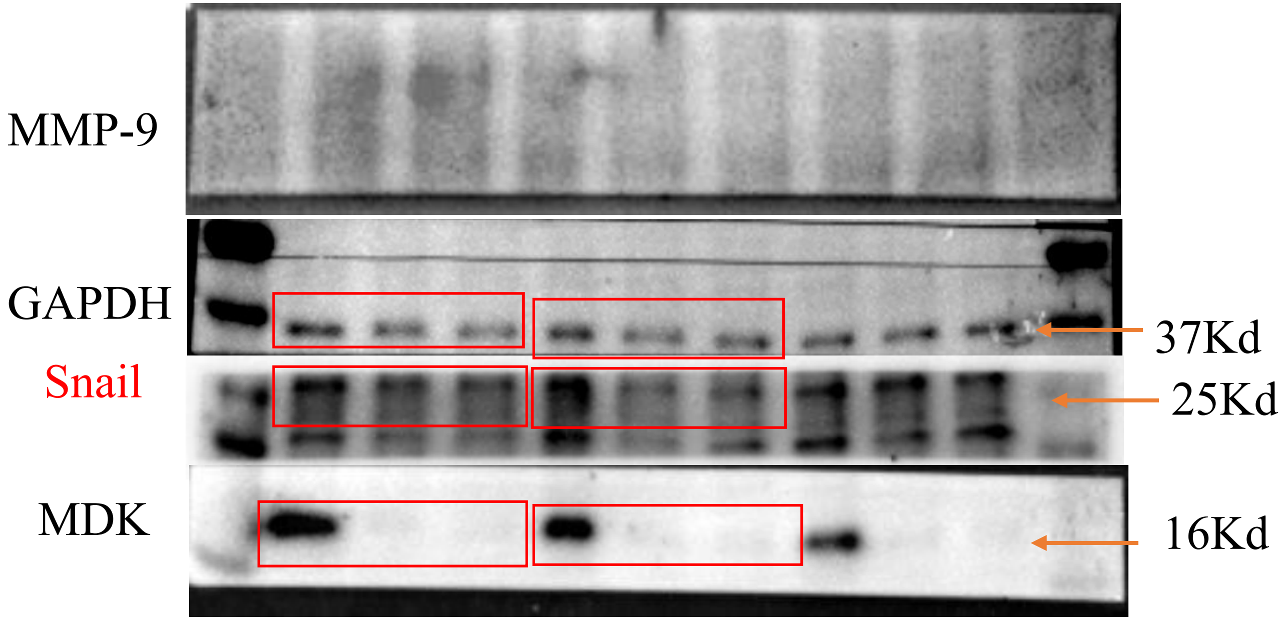

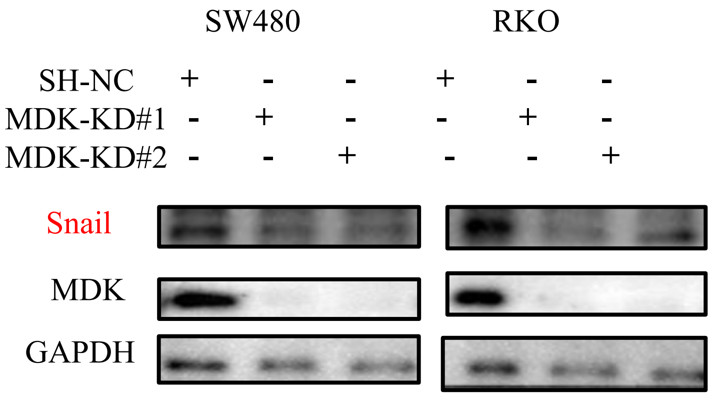

Figure 4A- Snail

Figure 4B-MMP2; 4D-MDK, GAPDH

Original uncropped image

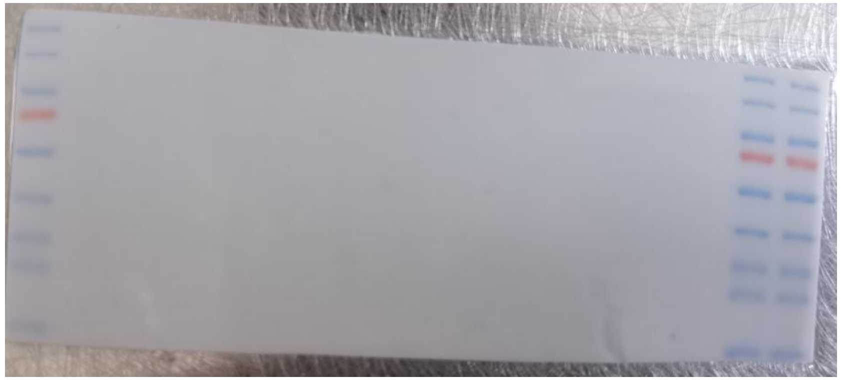

Original cropped image

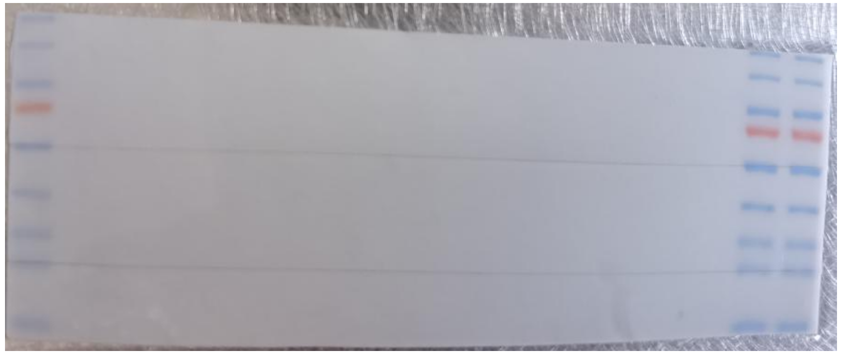

Original cropped image of the entire membrane

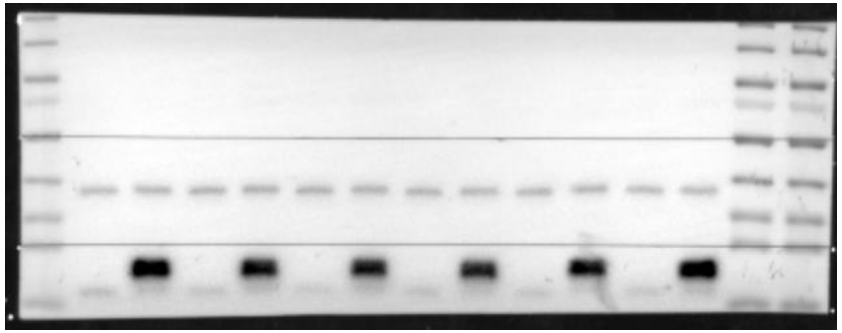

Original cropped image of the single membrane

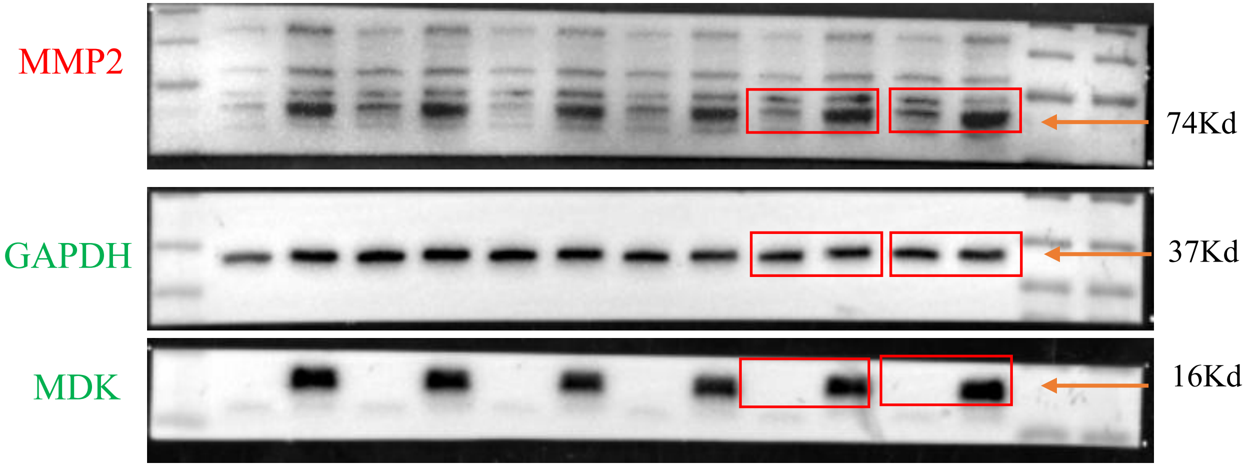

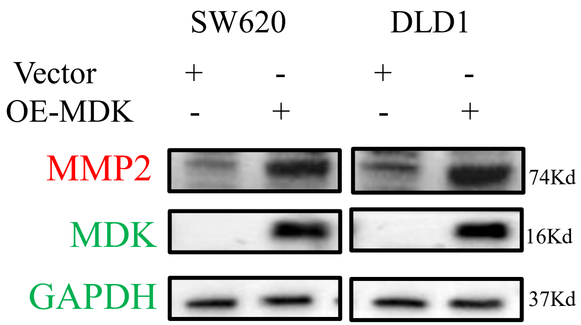

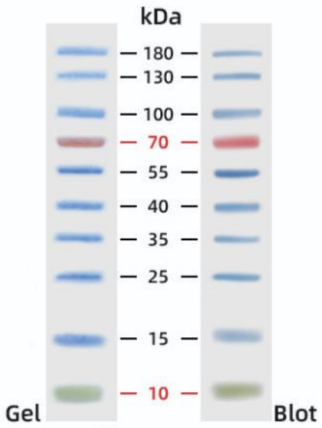

Figure 4B-MMP2; 4D-MDK, GAPDH

Figure 4B-MMP9

Original uncropped image

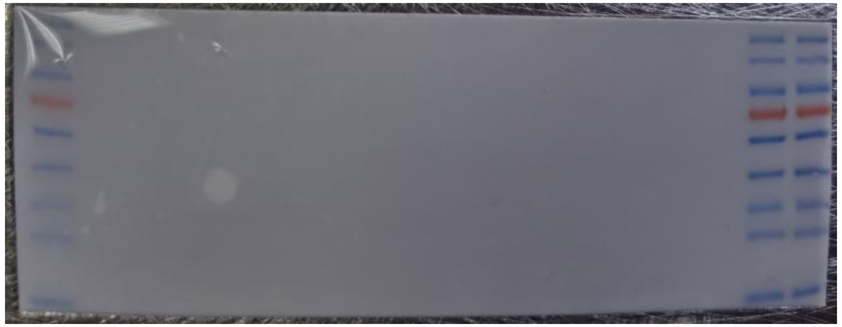

Original cropped image

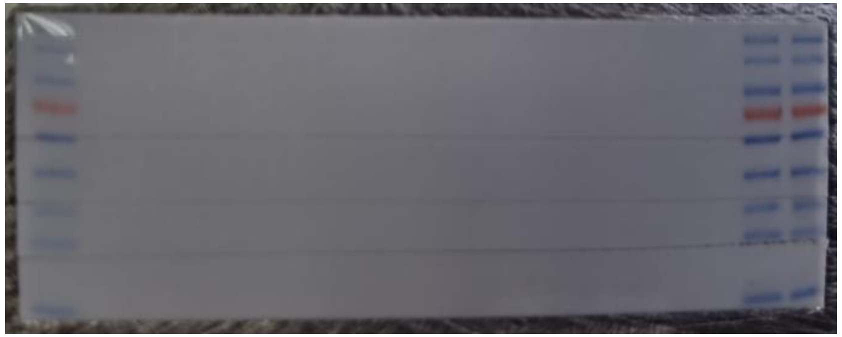

Original cropped image
of the entire membrane

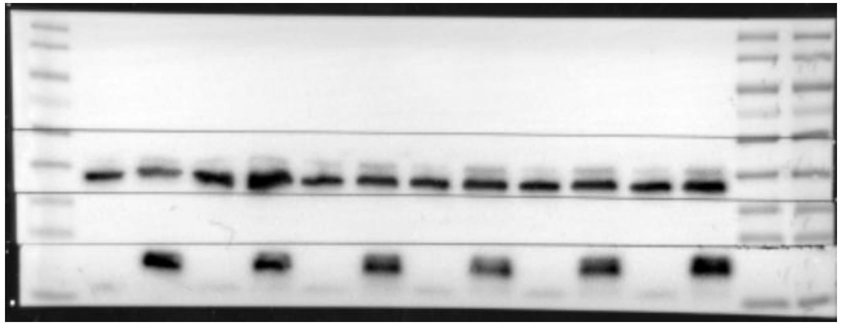

Original cropped image of the single membrane

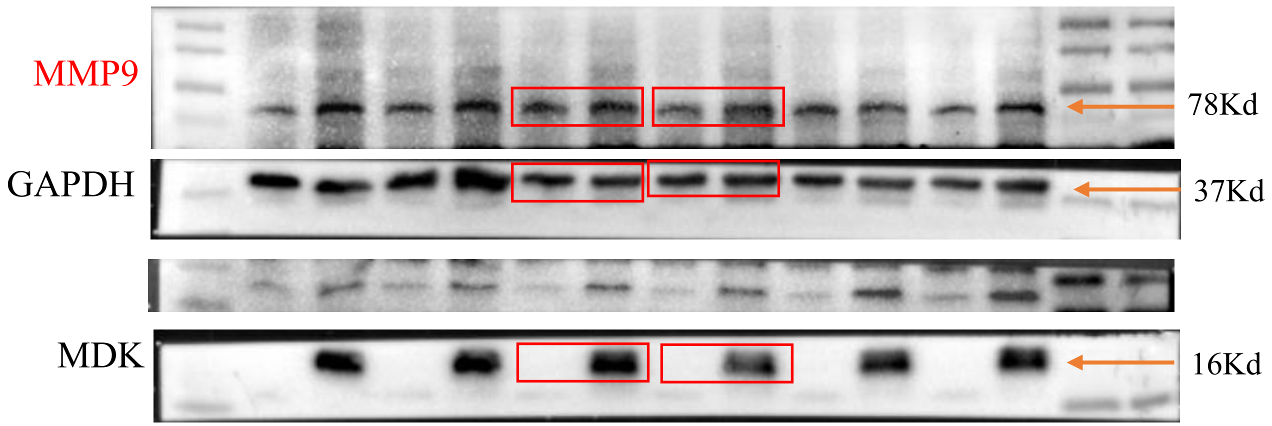

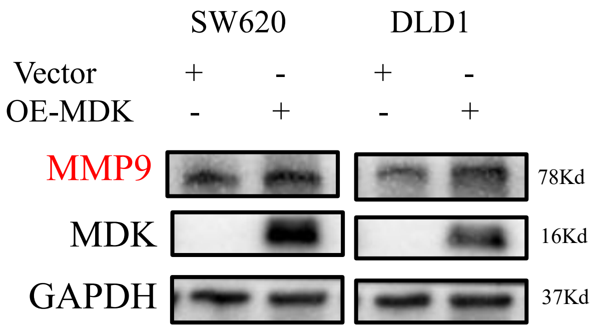

Figure 4B-MMP9

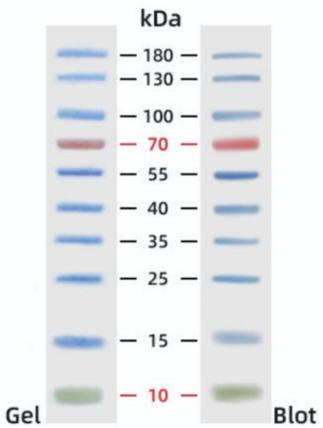

Figure 4B-Vimentin

Original uncropped image

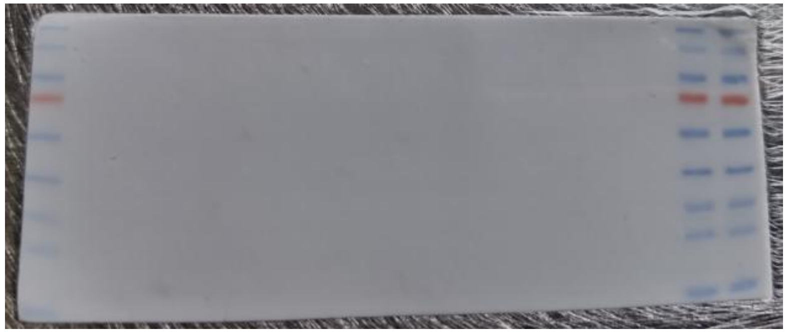

Original cropped image

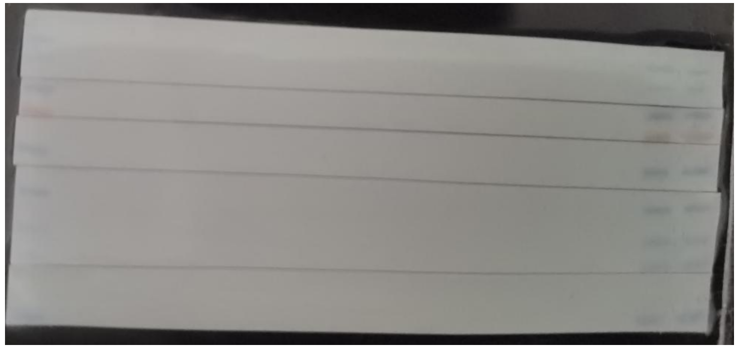

Original cropped image
of the entire membrane

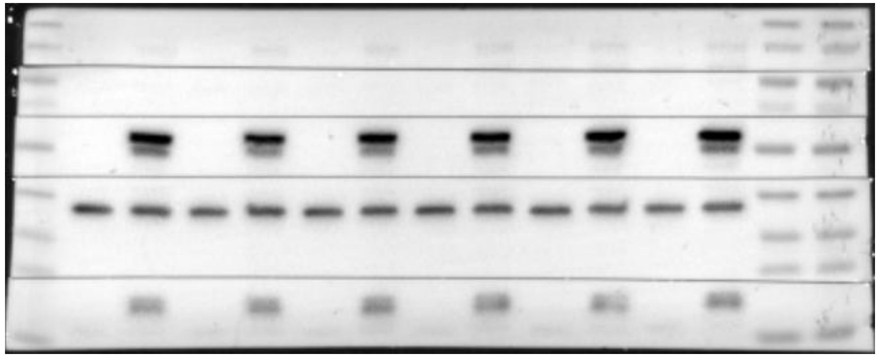

Original cropped image of the single membrane

Vimentin
GAPDH
MDK

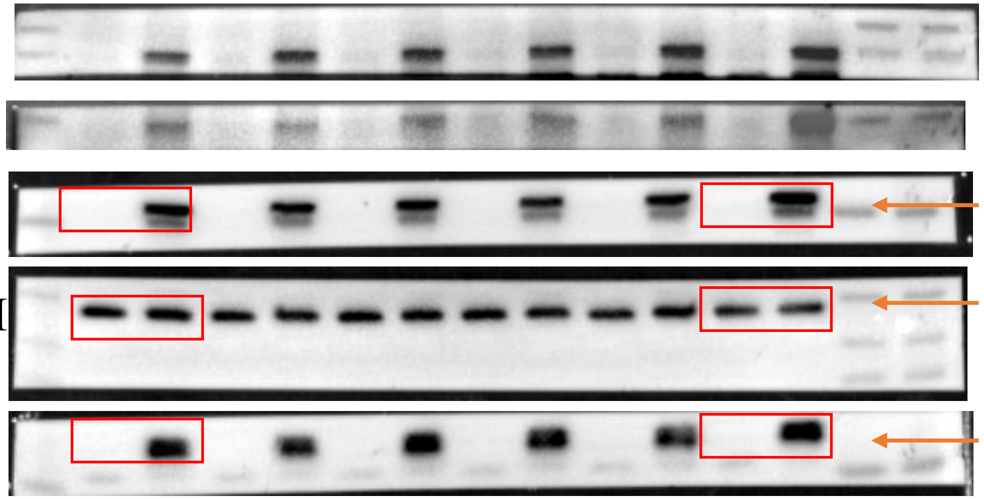

57Kd
37Kd
16Kd

SW620 DLD1

	SW620		DLD1	
Vector	+	-	+	-
OE-MDK	-	+	-	+

Vimentin
MDK
GAPDH

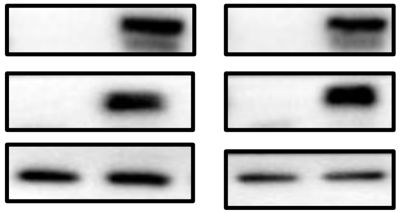

Figure 4B-Vimentin

Figure 4B- N-cad

Original uncropped image

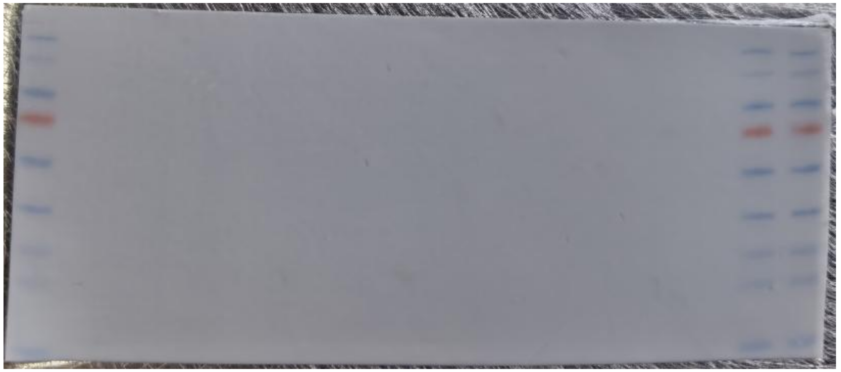

Original cropped image

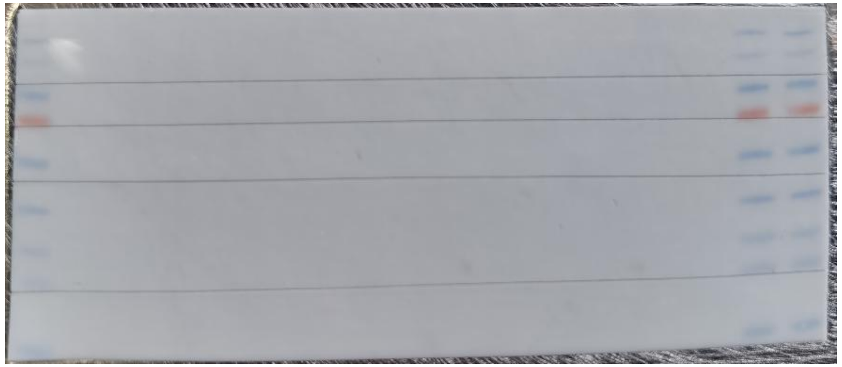

Original cropped image of the entire membrane

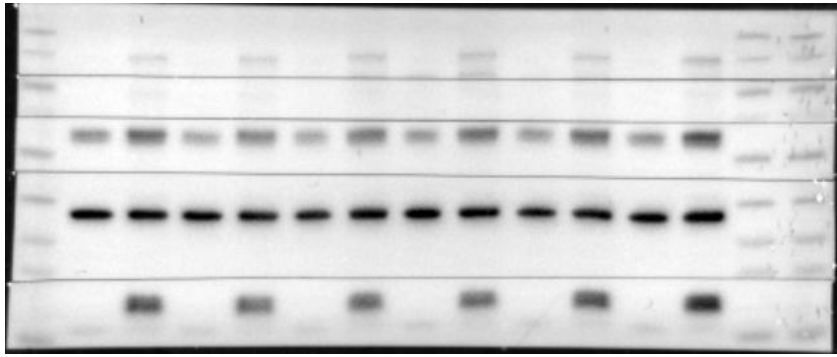

Original cropped image of the single membrane

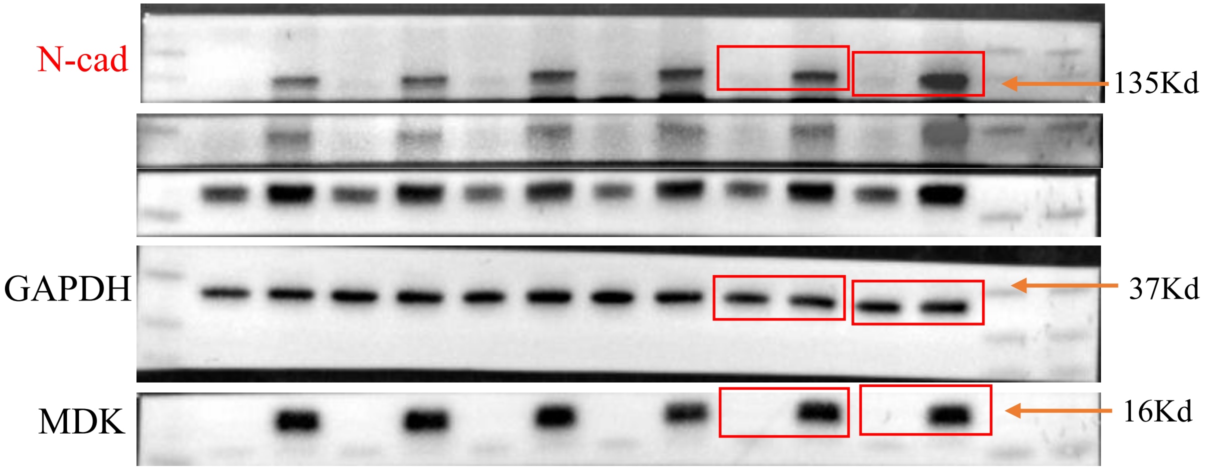

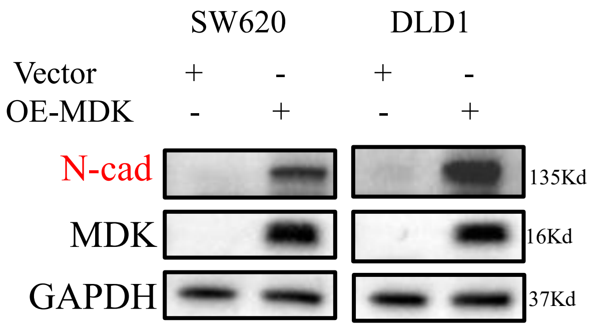

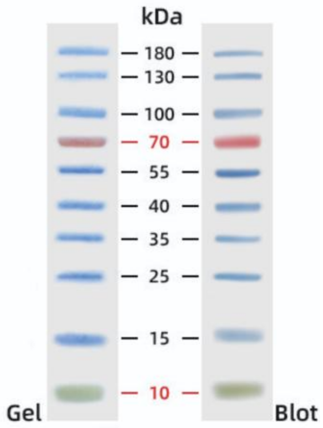

Figure 4B- N-cad

Figure 4B- β-catenin,Snail,MDK,GAPDH

Original uncropped image

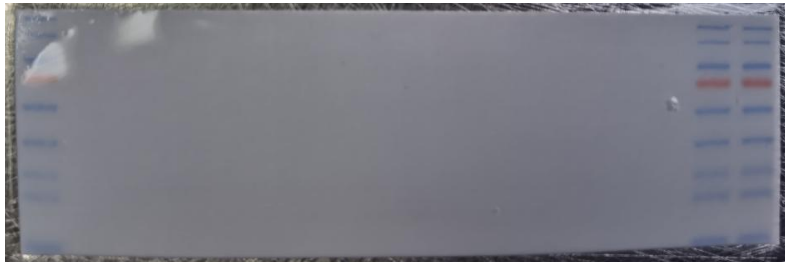

Original cropped image

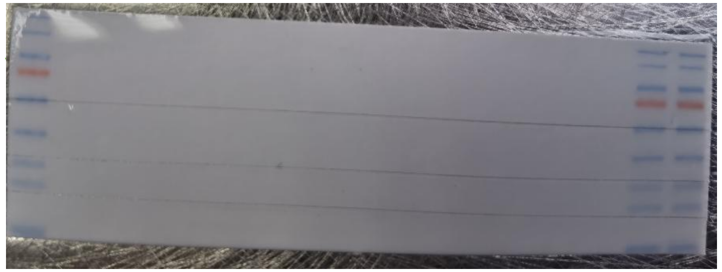

Original cropped image of the entire membrane

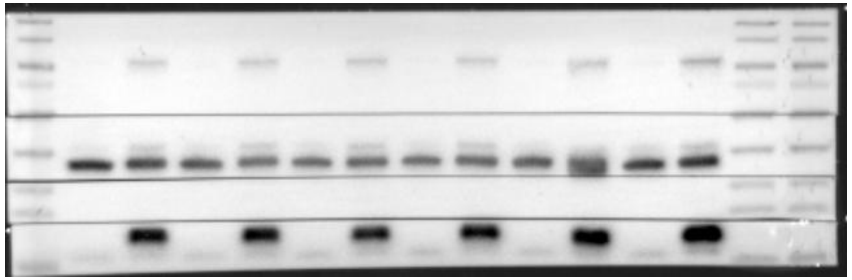

Original cropped image of the single membrane

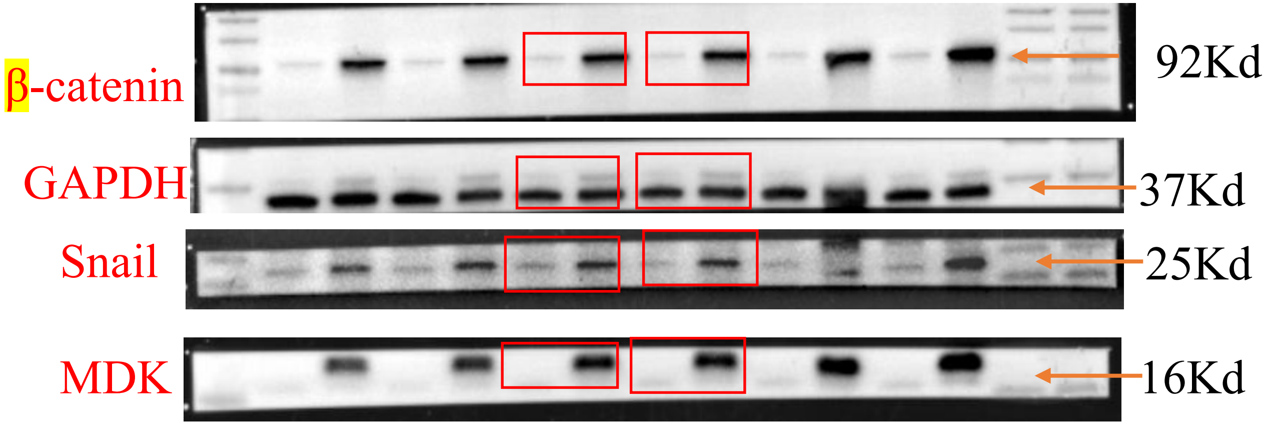

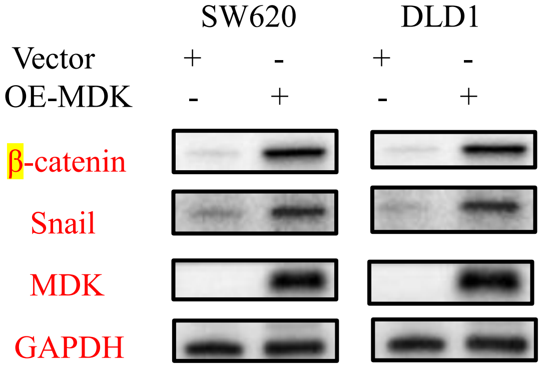

Figure 4B- β-catenin,Snail,MDK,GAPDH

Figure 4C- p-PI3K, p-ERK

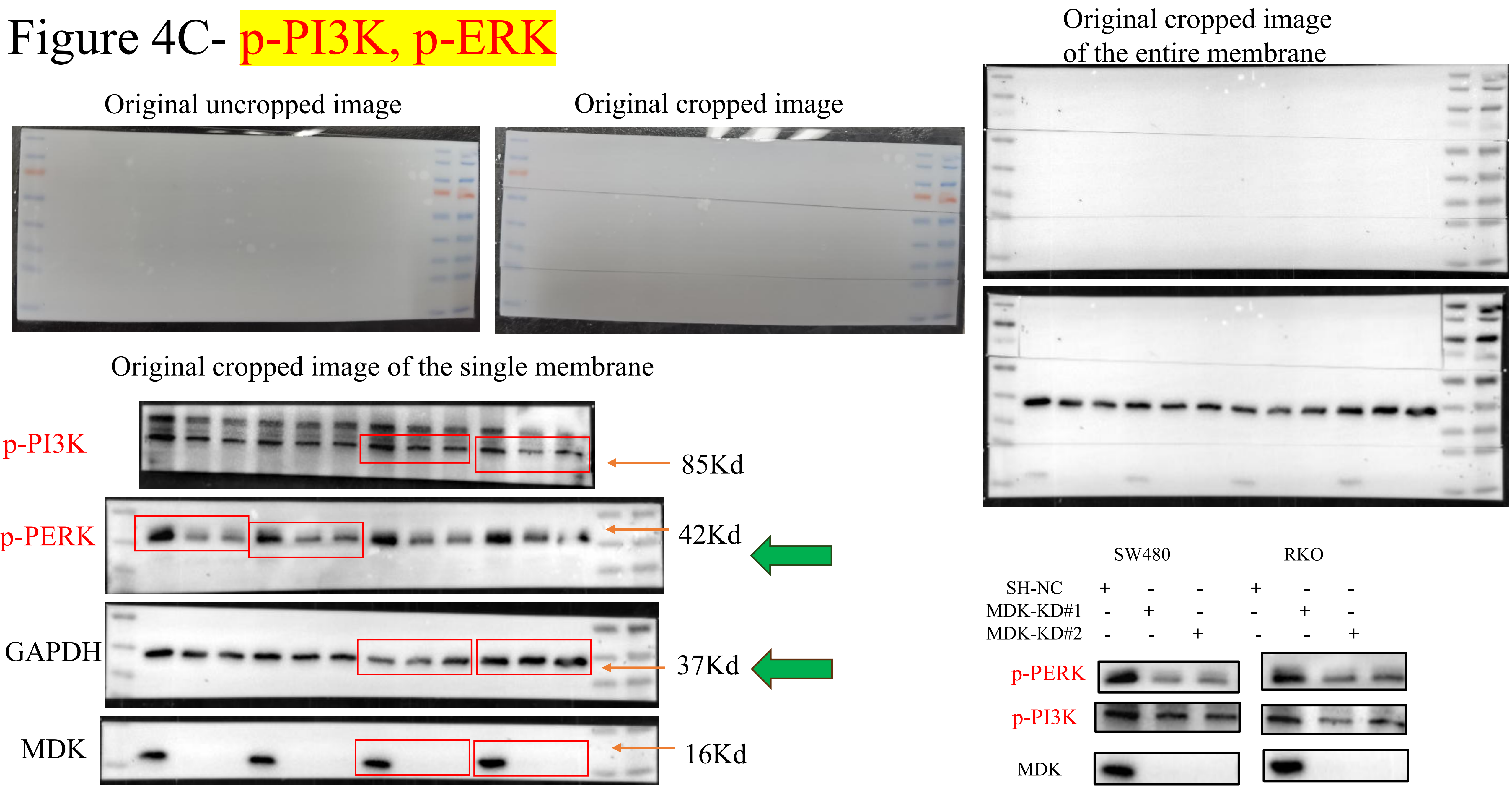

Note: using the same membrane for stripping

Figure 4C- PI3K

Original uncropped image

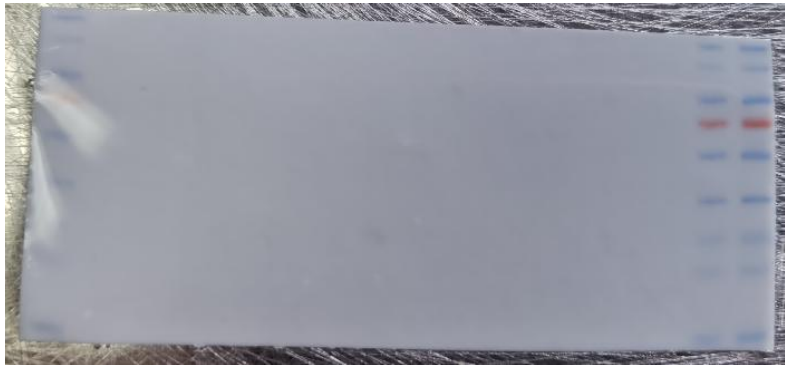

Original cropped image

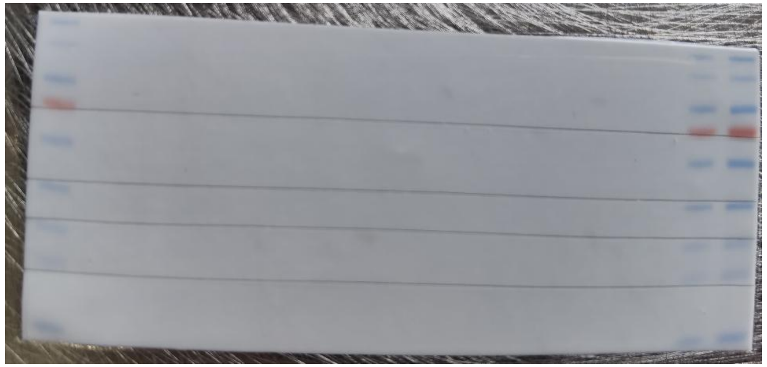

Original cropped image of the entire membrane

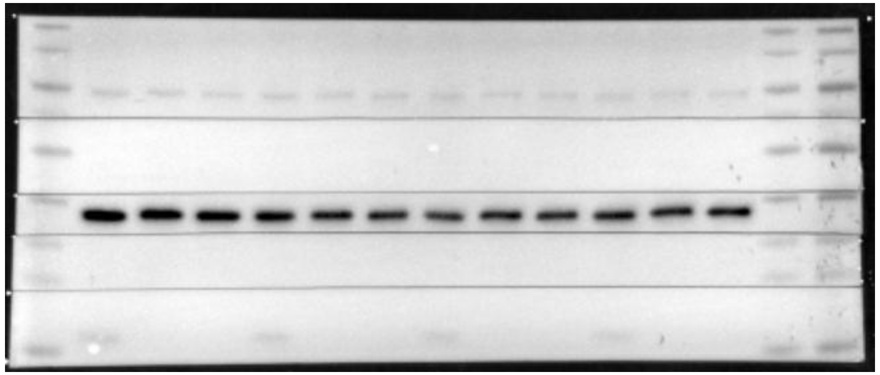

Original cropped image of the single membrane

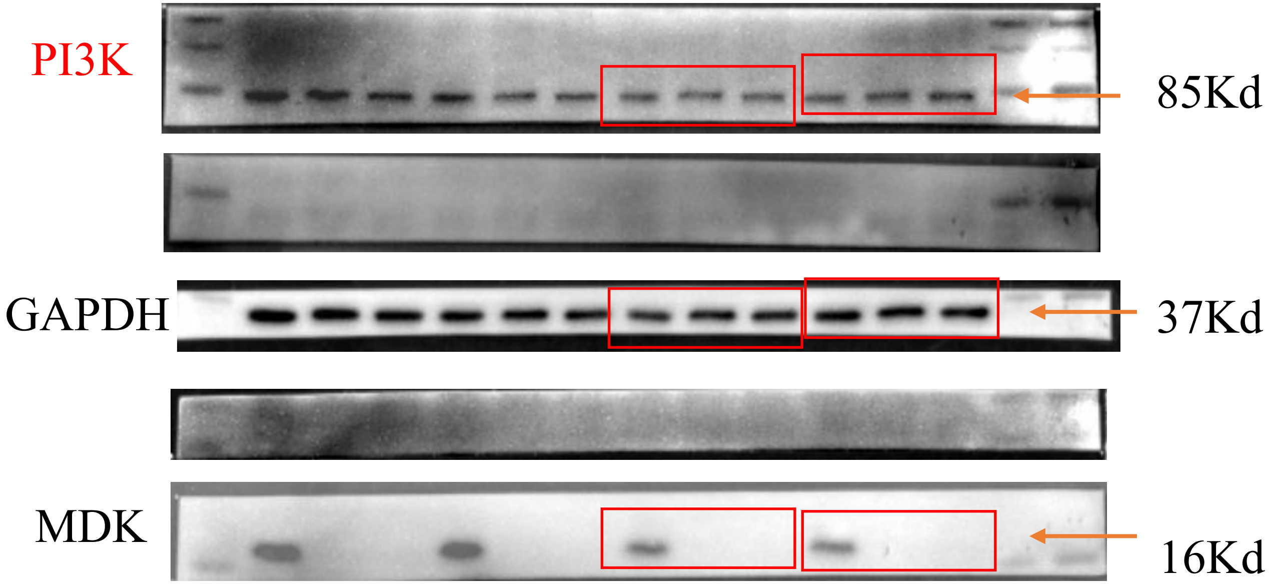

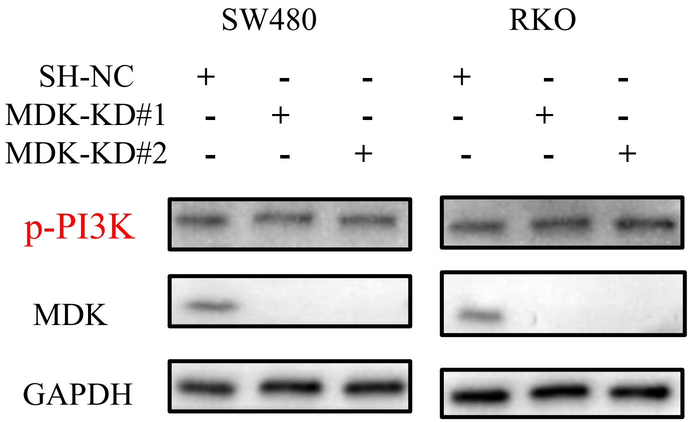

Figure 4C- PI3K

Figure 4C-ERK

Original uncropped image

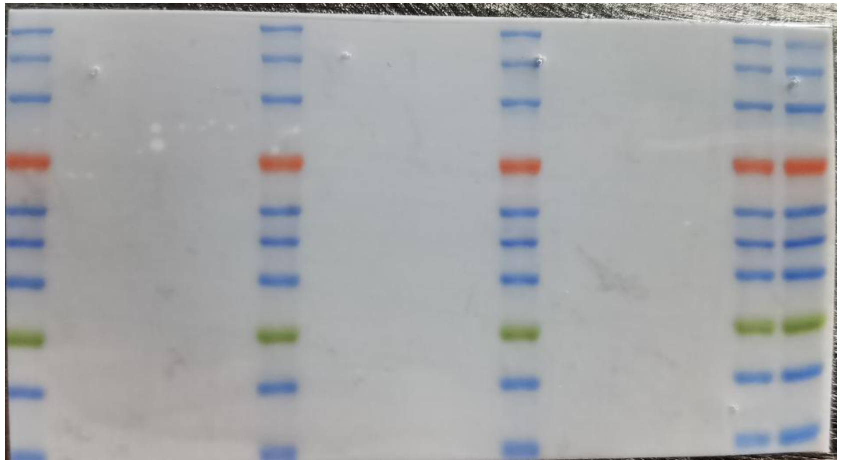

Original cropped image

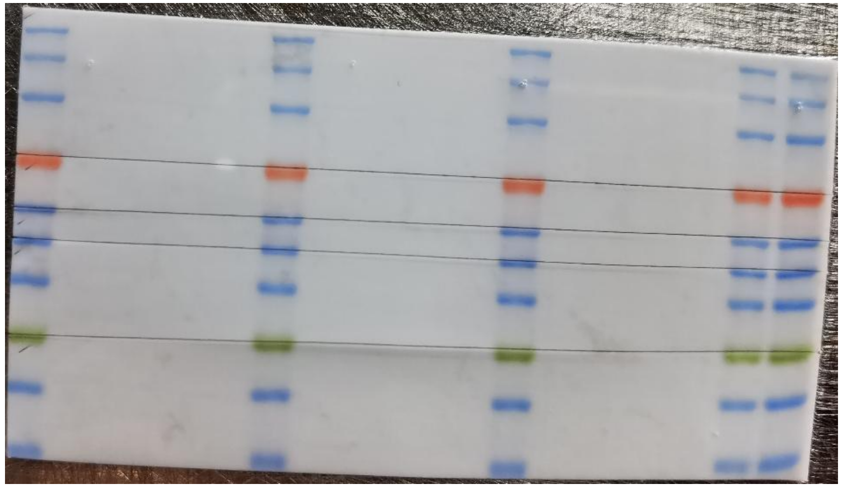

Original cropped image of the entire membrane

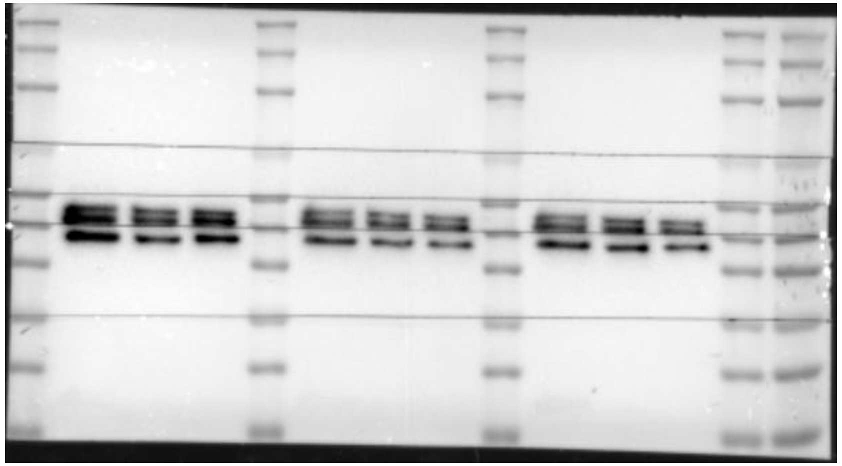

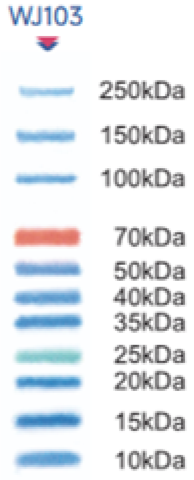

Original cropped image of the single membrane

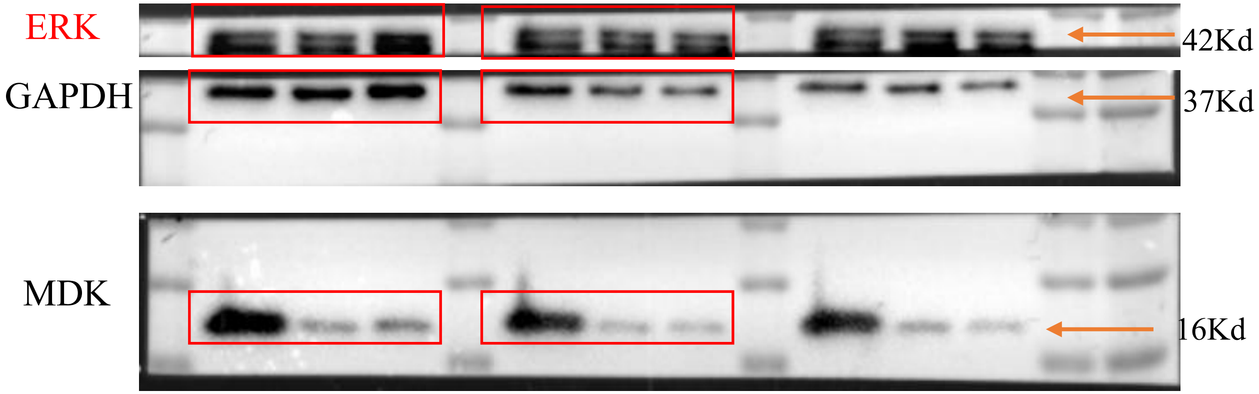

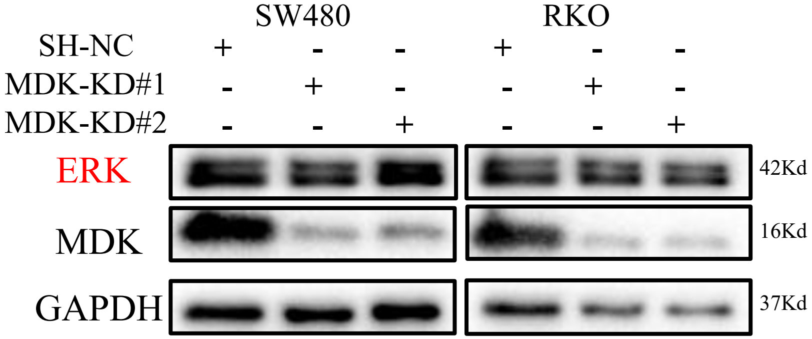

Figure 4C-ERK

Figure 4C- AKT, p-AKT; Figure 5H-AP2A1,MDK,GAPDH

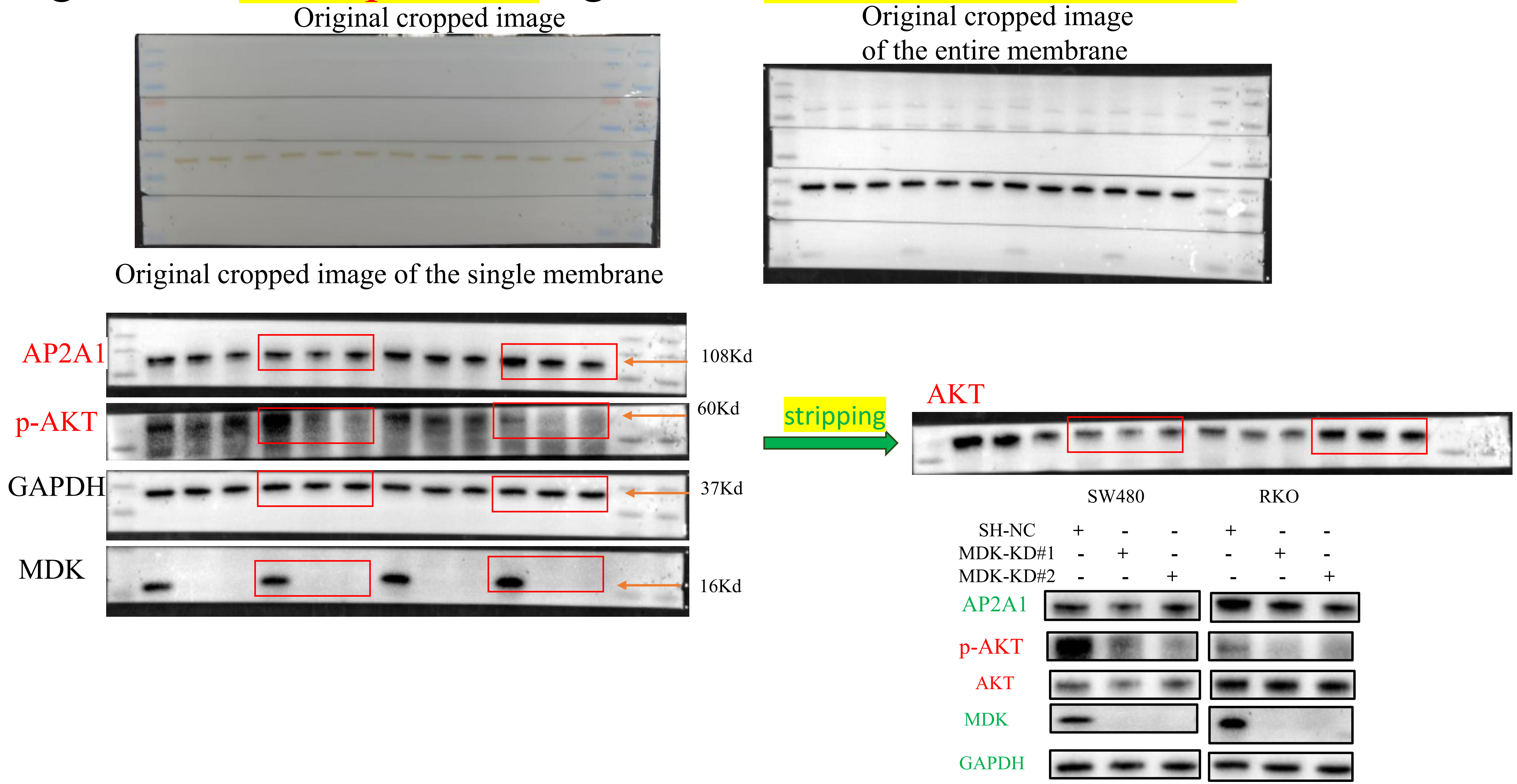

Figure 4C- MDK, GAPDH

Original uncropped image

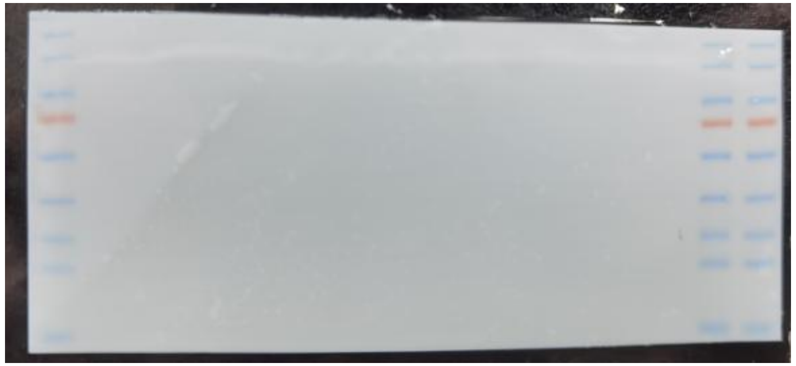

Original cropped image

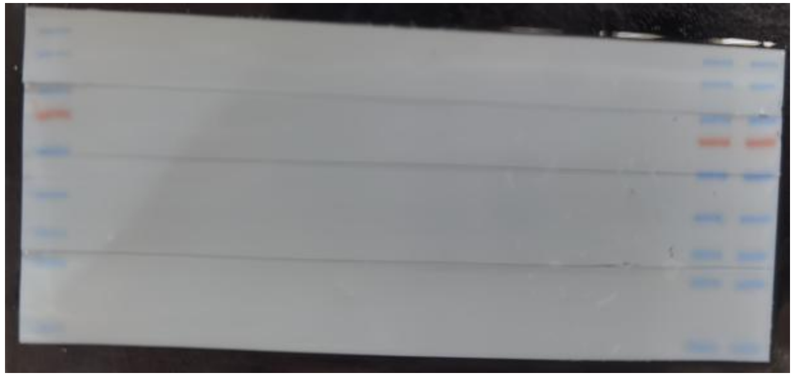

Original cropped image of the entire membrane

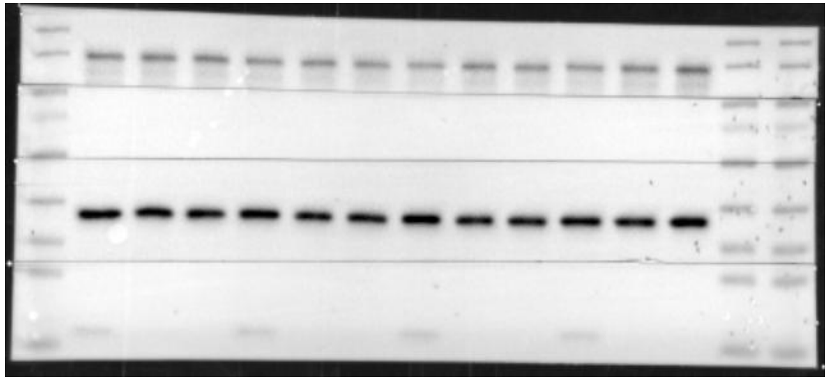

Original cropped image of the single membrane

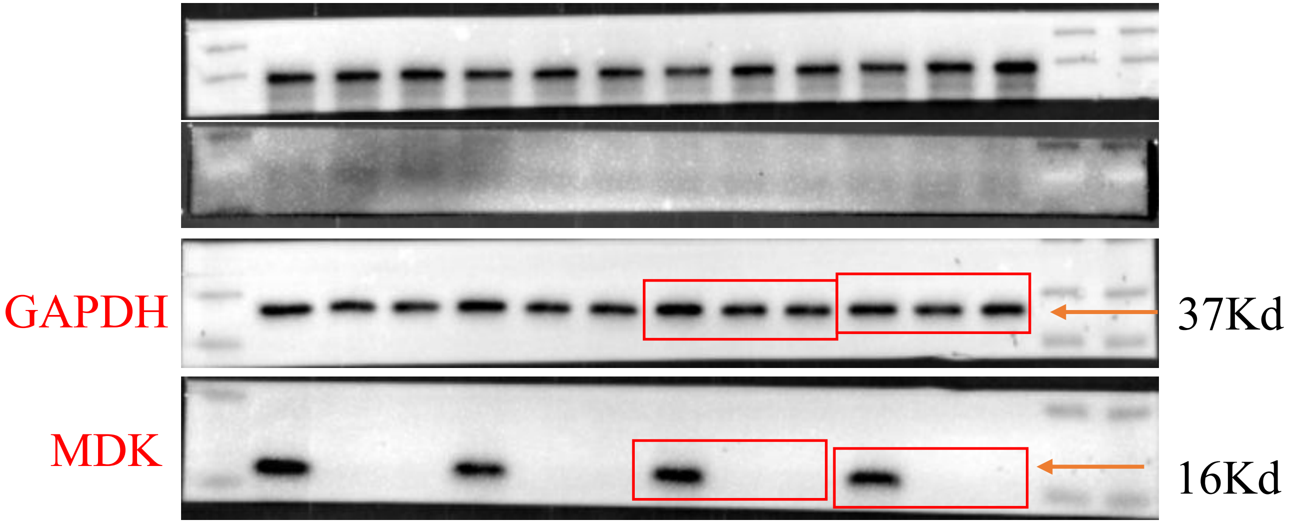

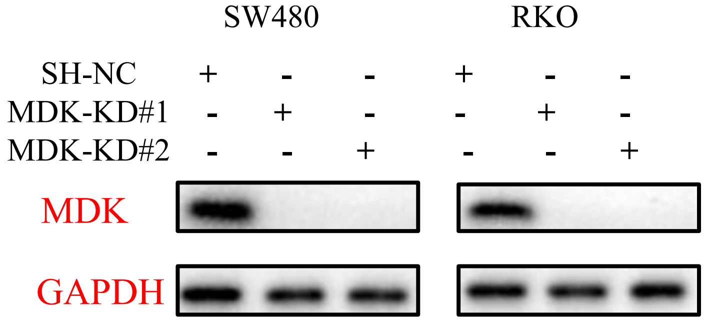

Figure 4D-p-PI3K

Original uncropped image

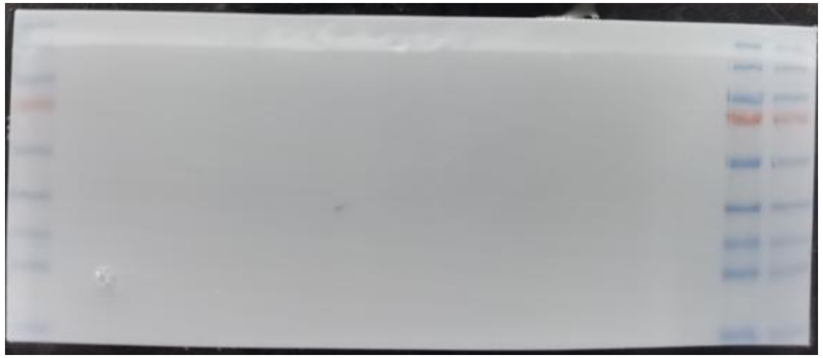

Original cropped image

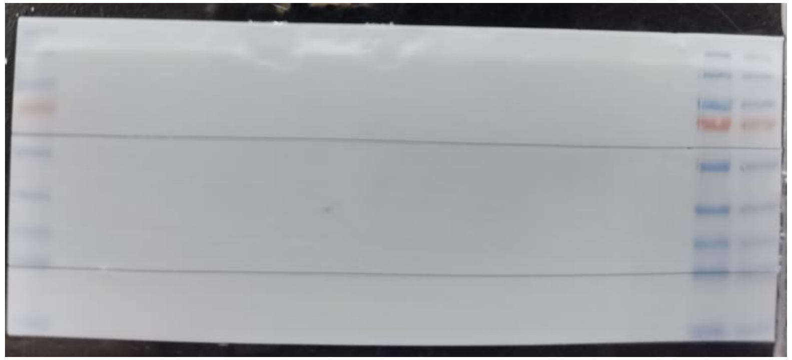

Original cropped image of the entire membrane

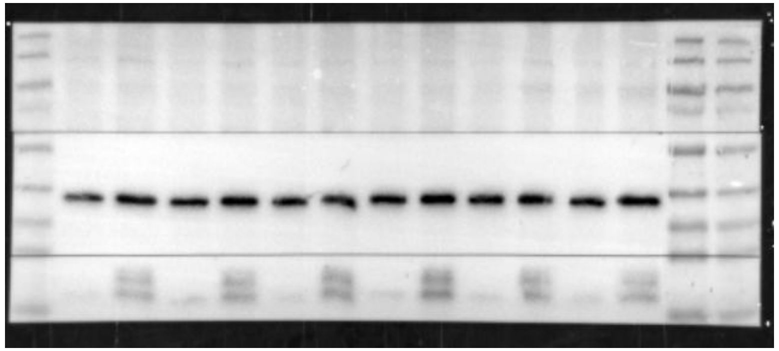

Original cropped image of the single membrane

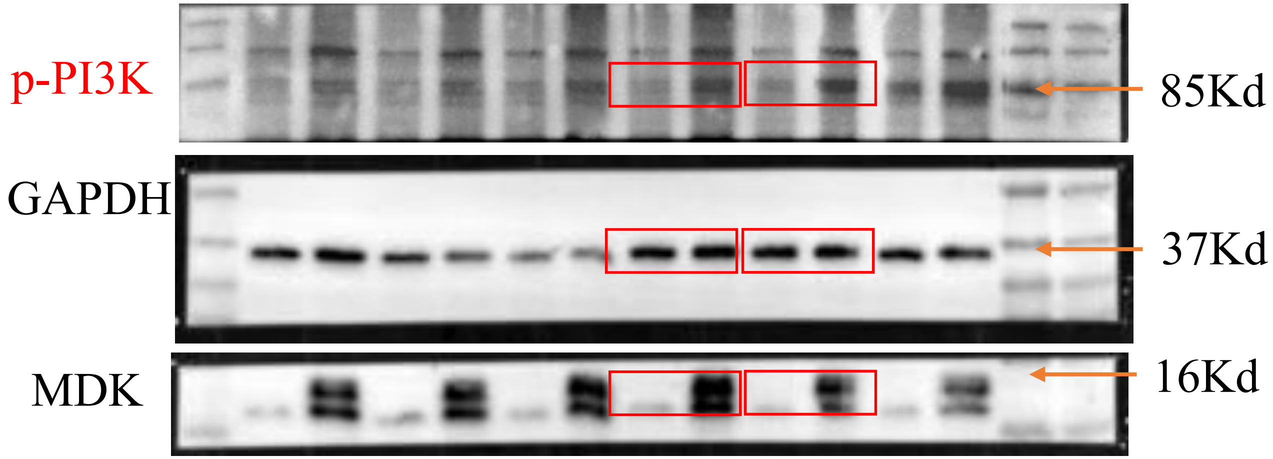

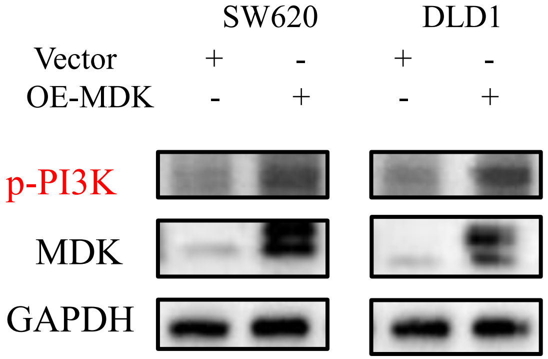

Figure 4D-p-PI3K

Figure 4D-PI3K

Original uncropped image

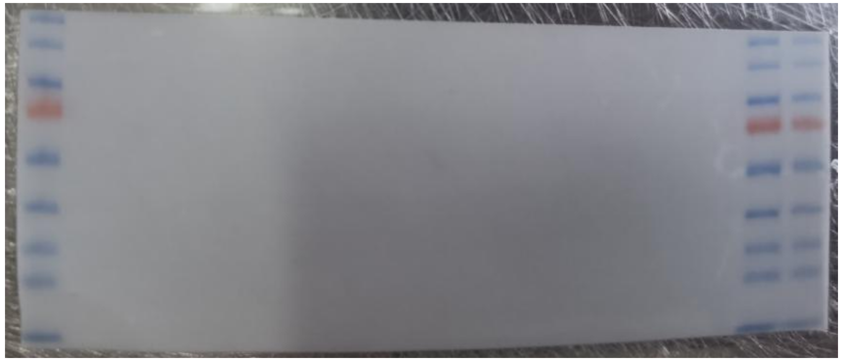

Original cropped image

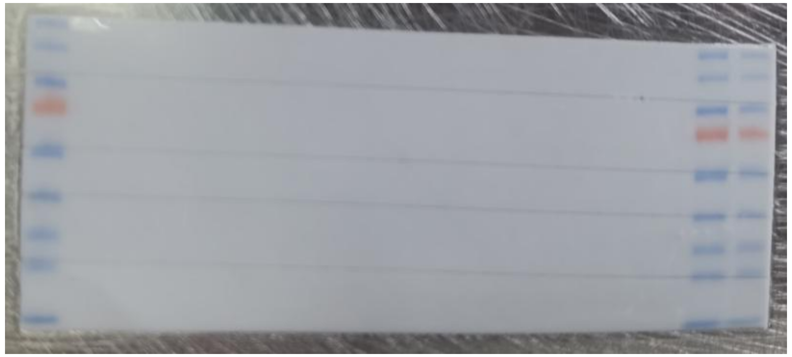

Original cropped image
of the entire membrane

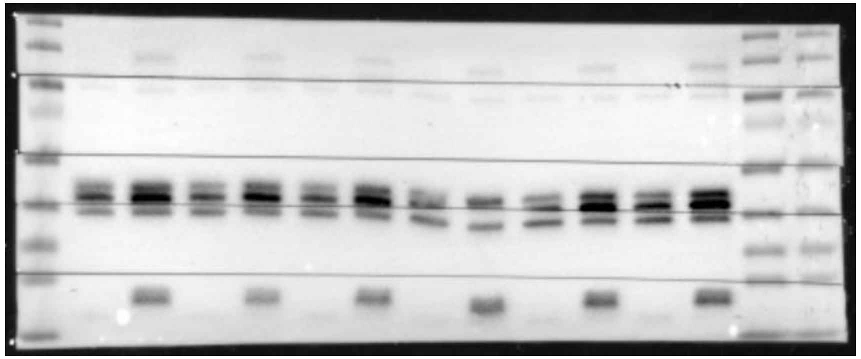

Original cropped image of the single membrane

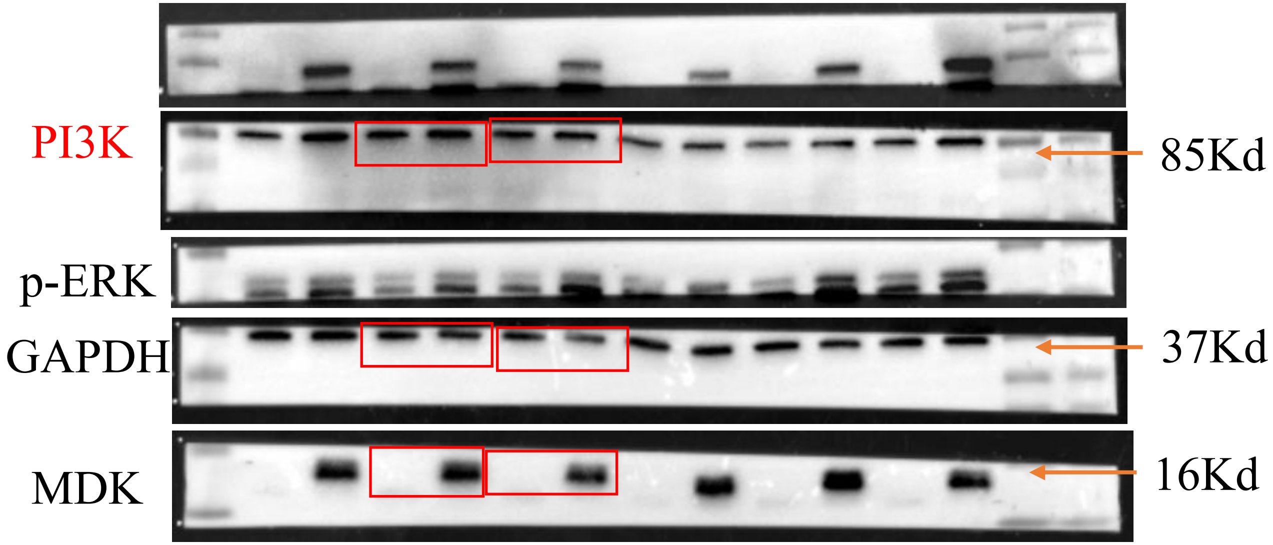

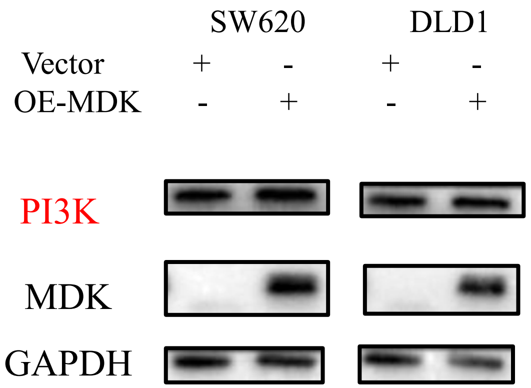

Figure 4D-PI3K

Figure 4D-p-ERK

Original uncropped image

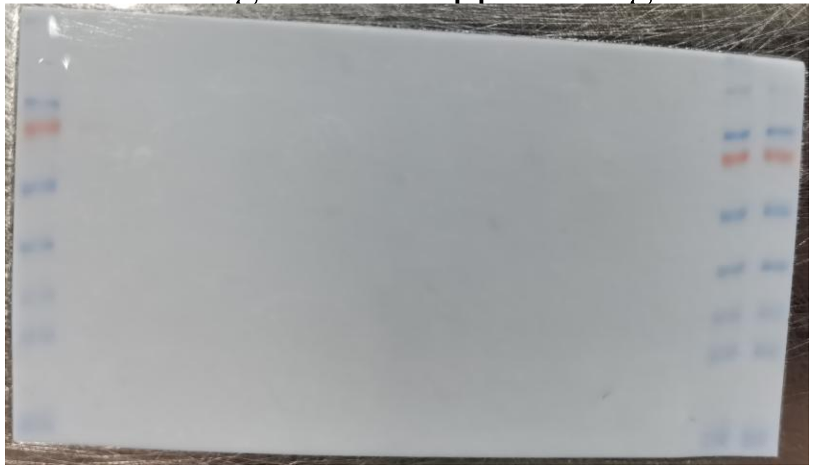

Original cropped image

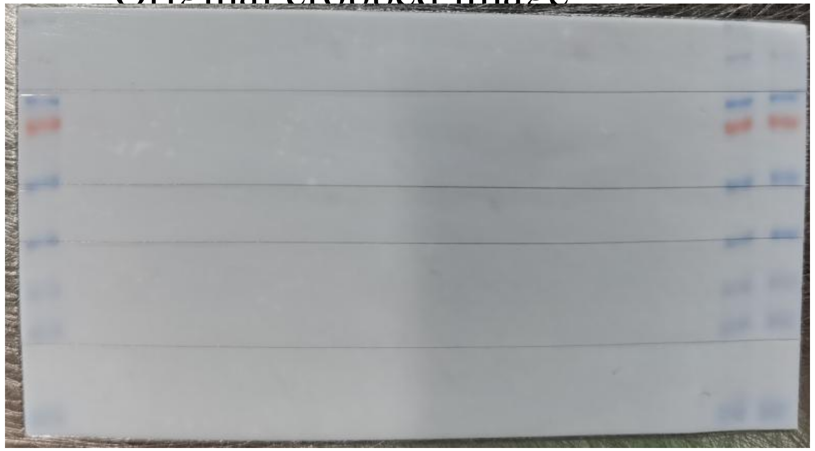

Original cropped image of the entire membrane

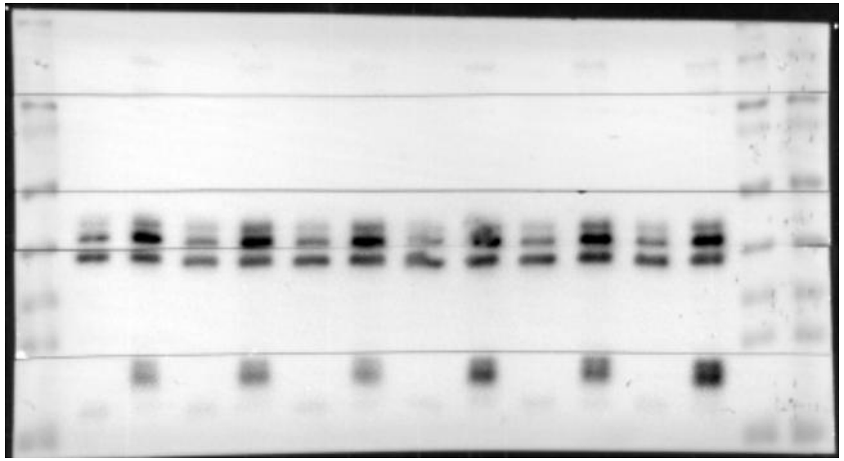

Original cropped image of the single membrane

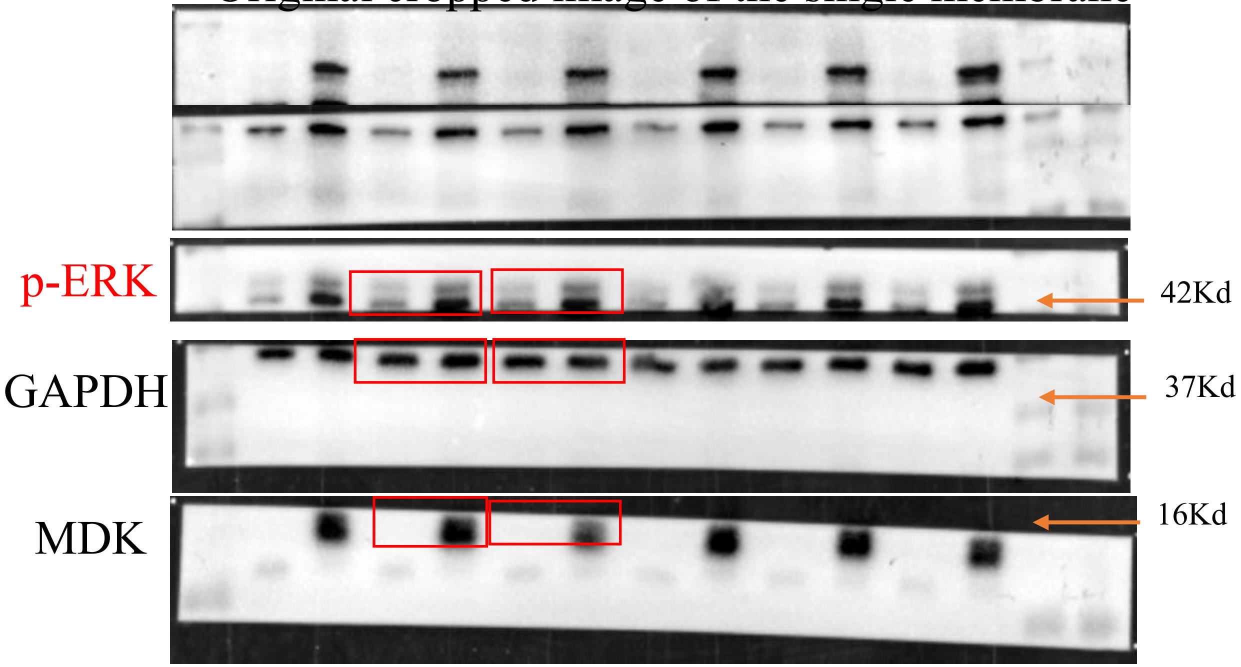

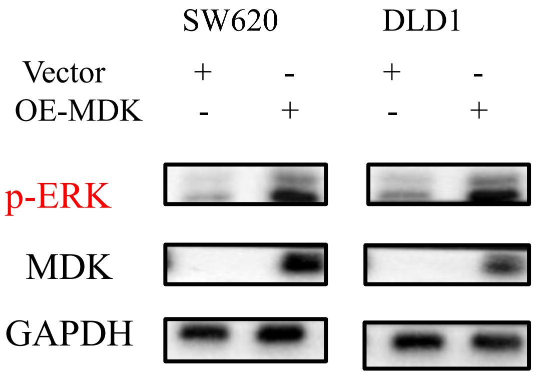

Figure 4D-p-ERK

Figure 4D-ERK

Original uncropped image

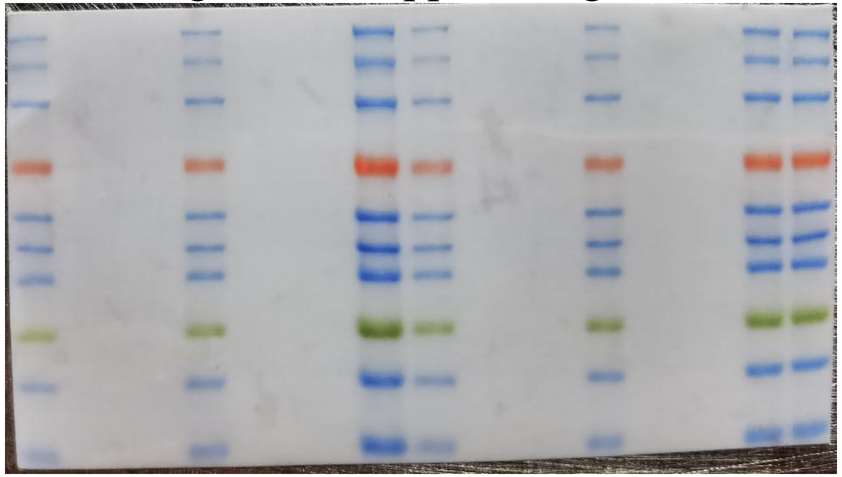

Original cropped image

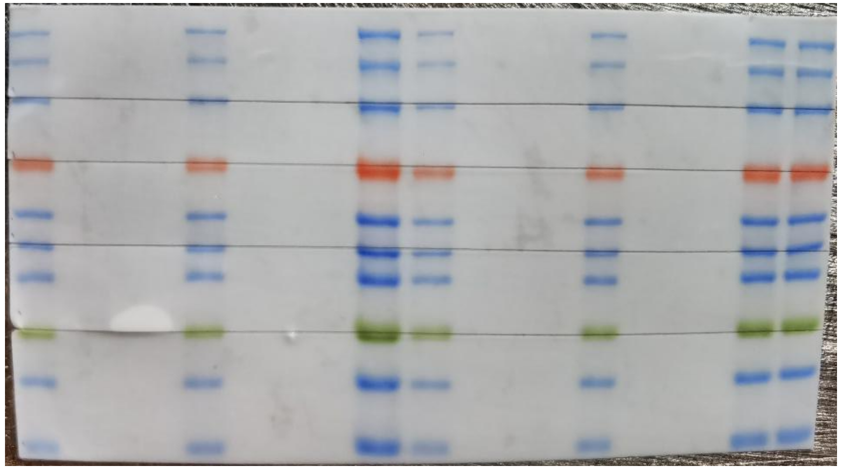

Original cropped image of the entire membrane

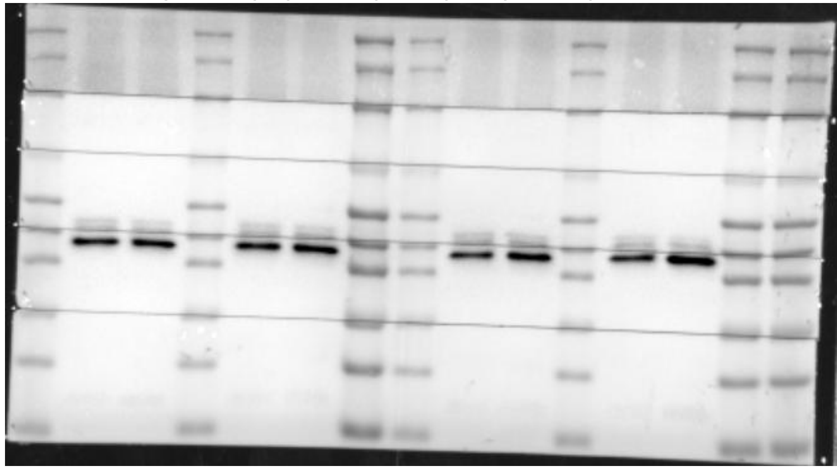

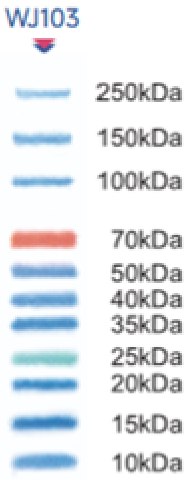

Original cropped image of the single membrane

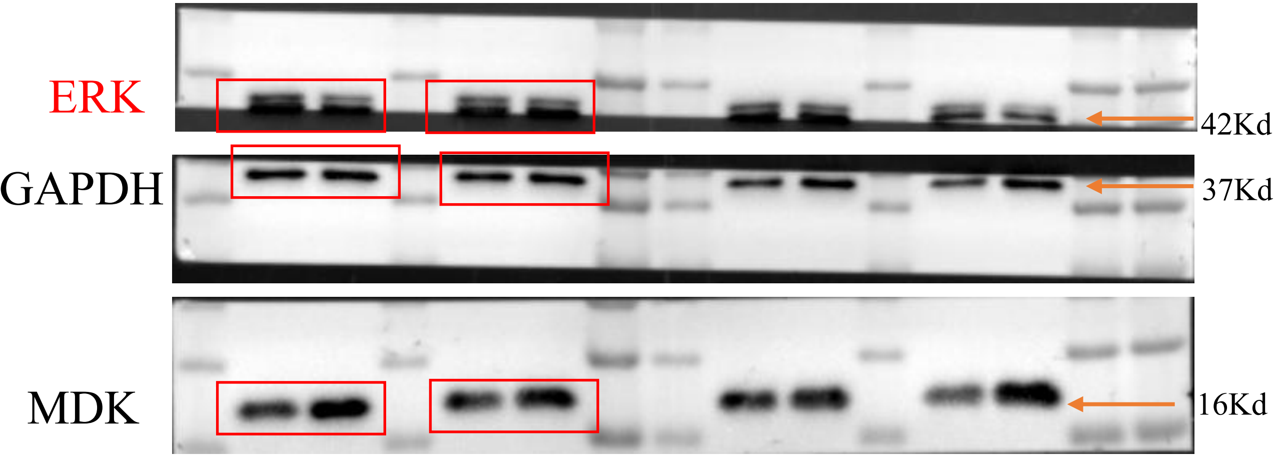

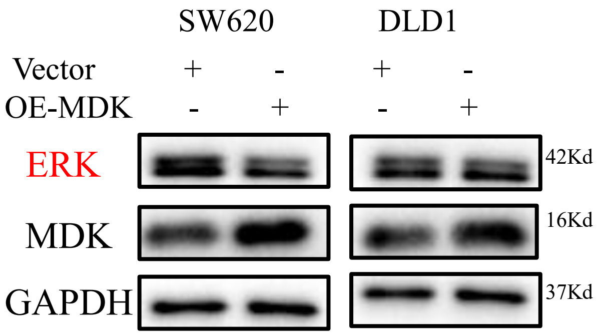

Figure 4D-ERK

Figure 4D-p-AKT

Original uncropped image

Original cropped image

Original cropped image of the entire membrane

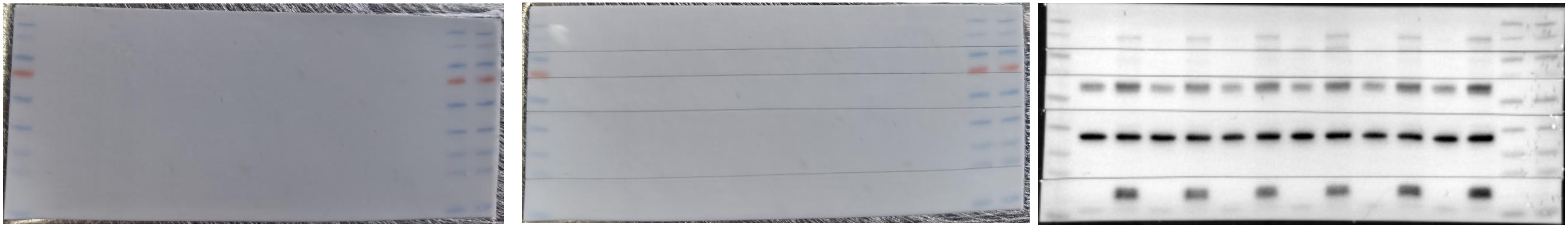

Original cropped image of the single membrane

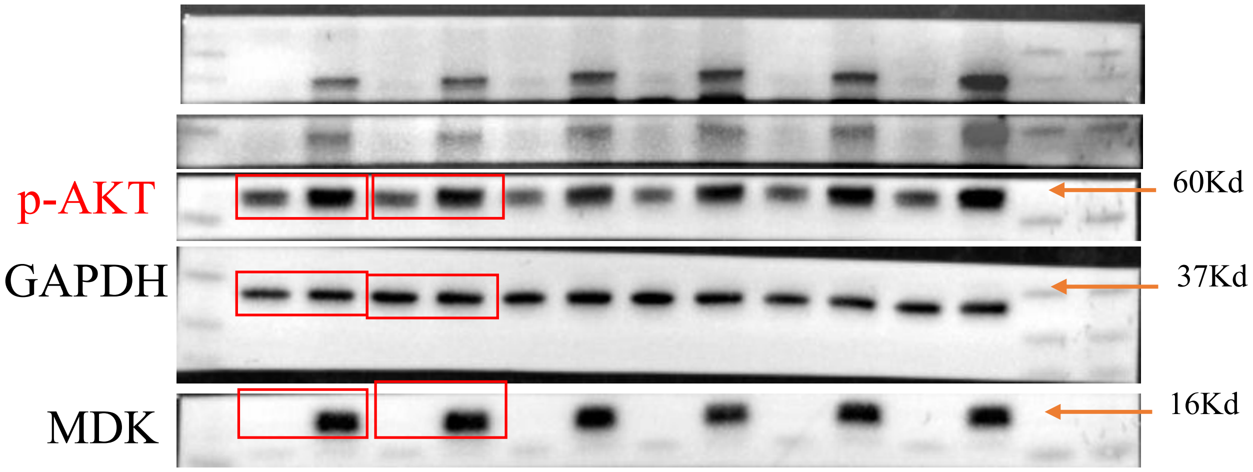

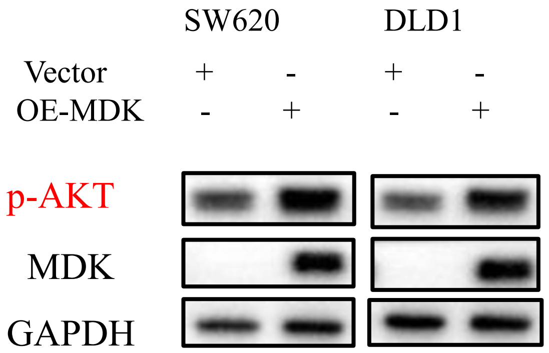

Figure 4D-p-AKT

Figure 4D-AKT;

Original uncropped image

Original cropped image

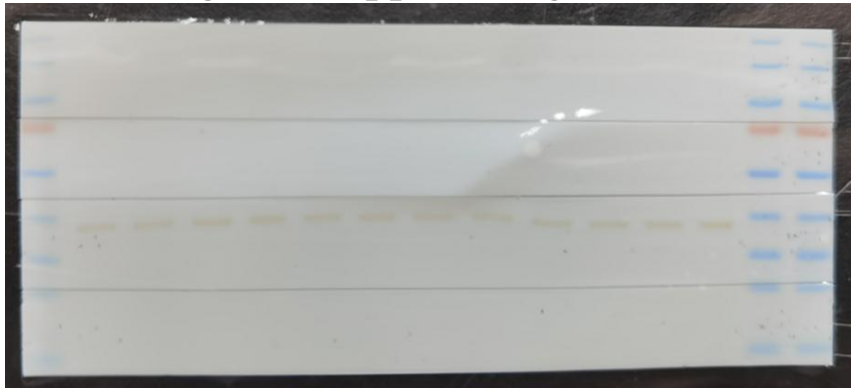

Original cropped image of the entire membrane

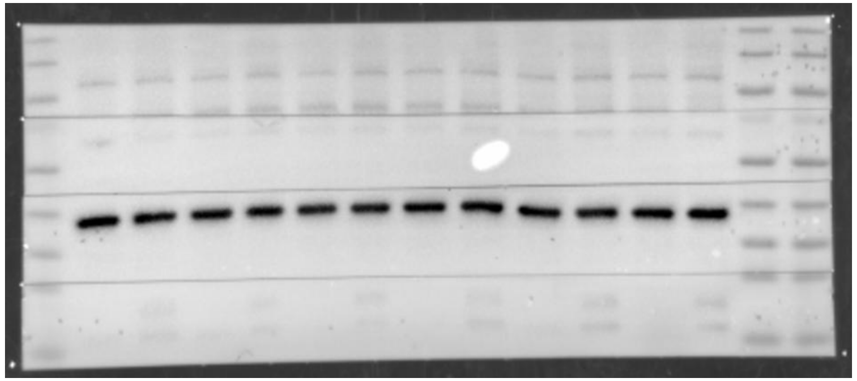

Original cropped image of the single membrane

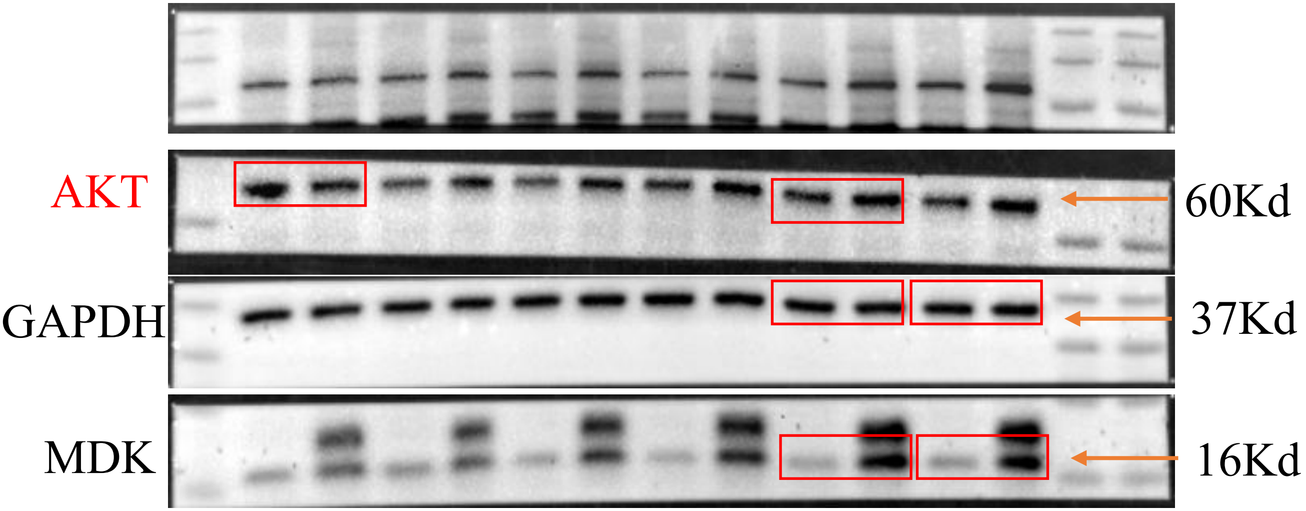

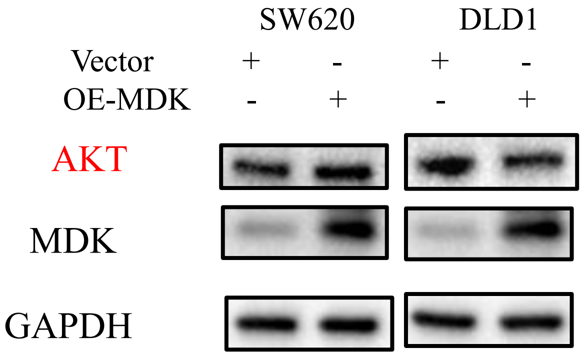

Figure 5I-AP2A1,MDK,GAPDH

Original uncropped image

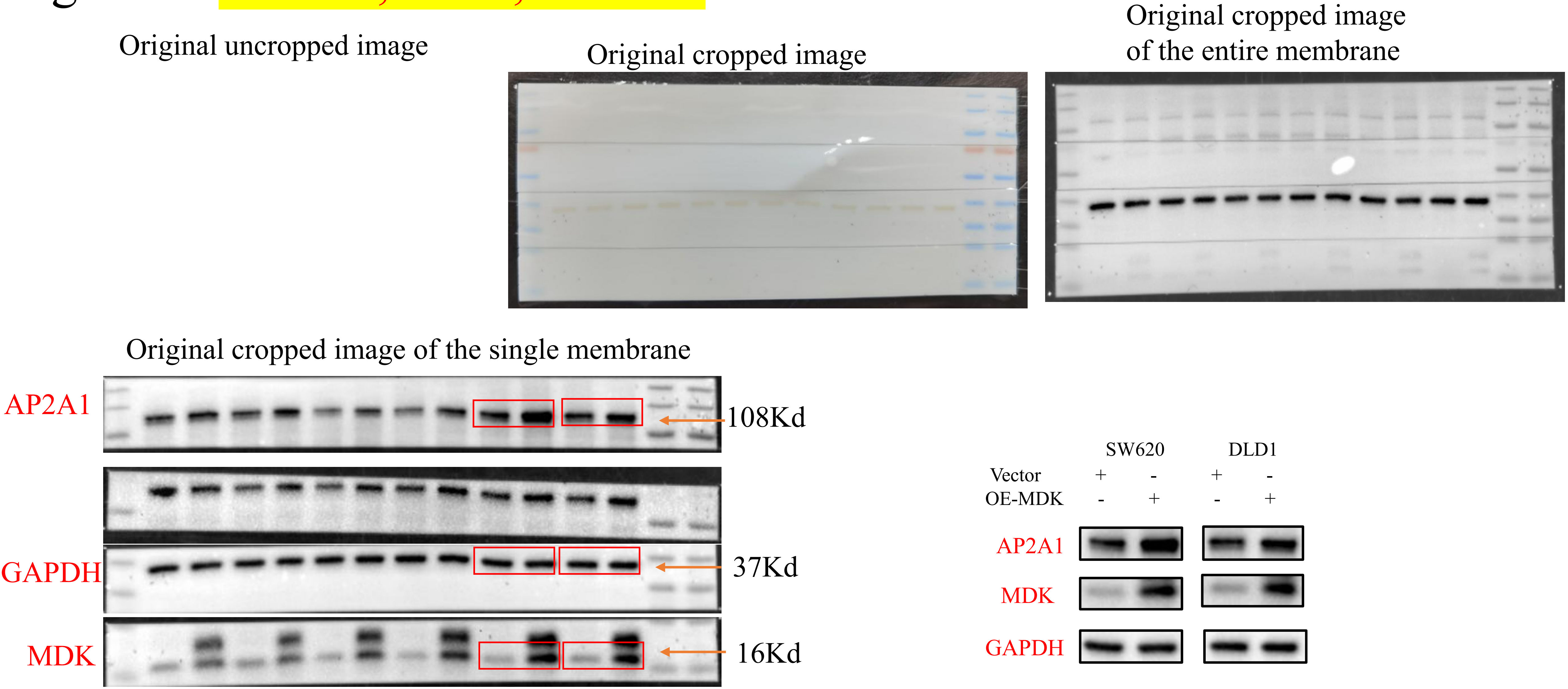

Figure 6A

Original uncropped image

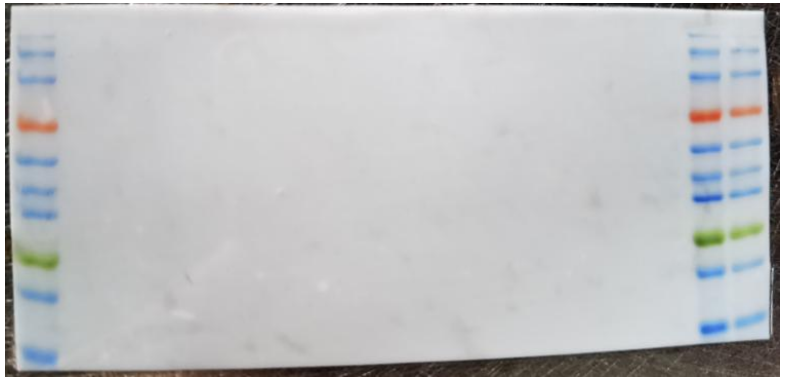

Original cropped image

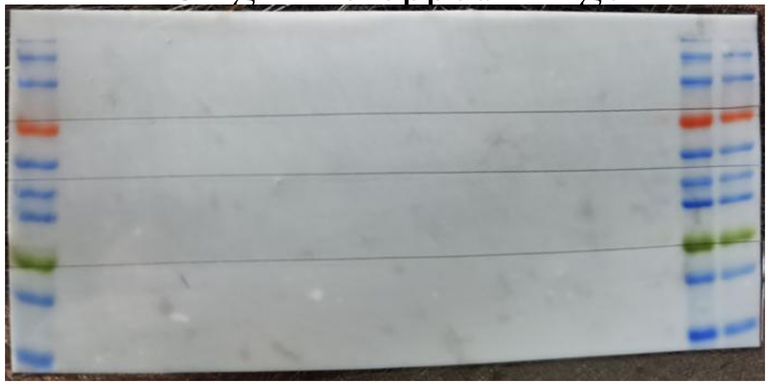

Original cropped image
of the entire membrane

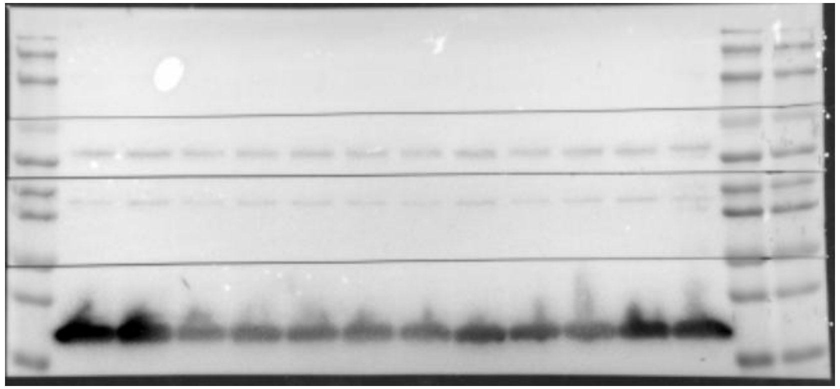

Original cropped image of the single membrane

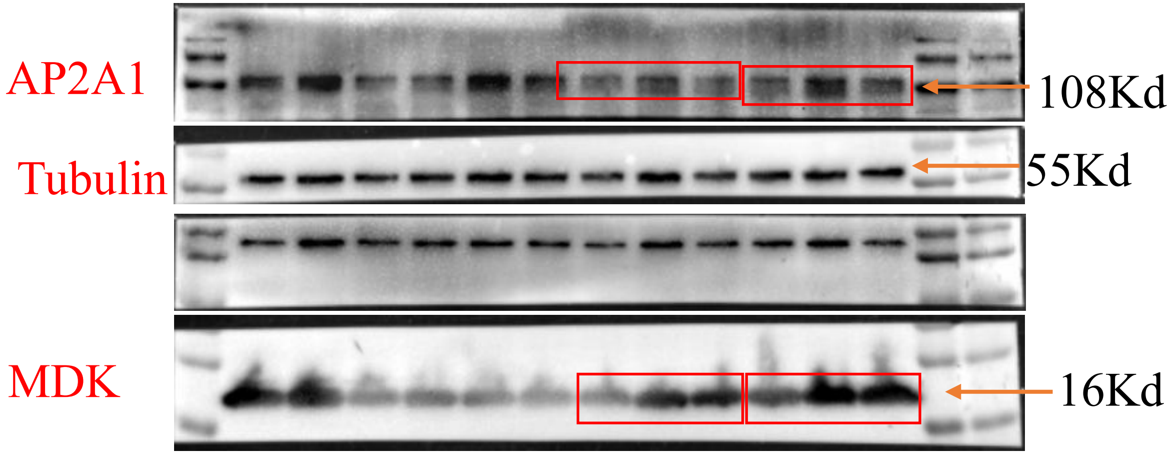

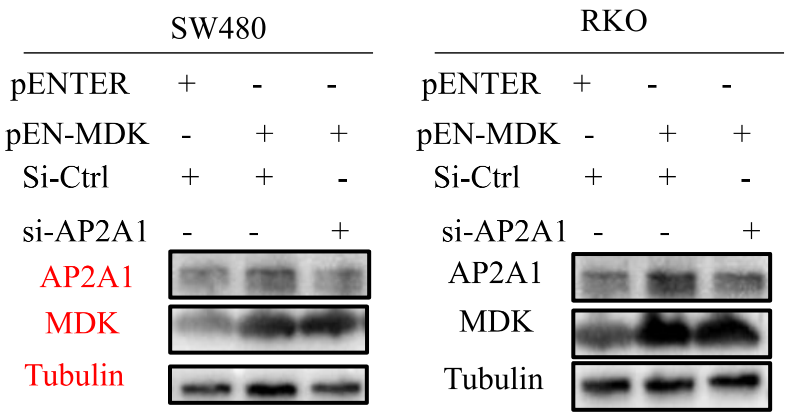

Figure 6B; 8B-MDK

Original uncropped image

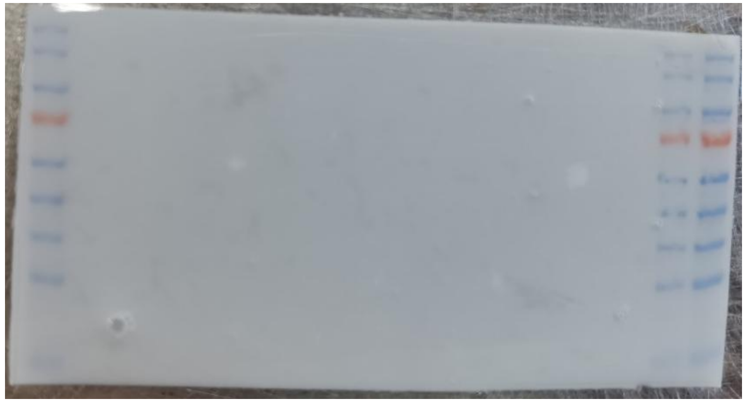

Original cropped image

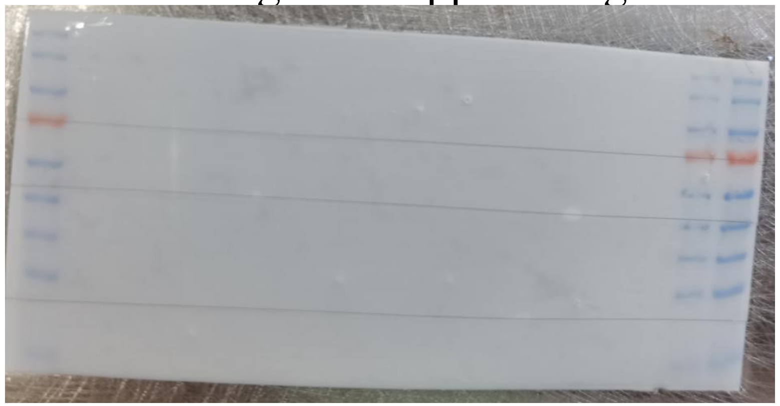

Original cropped image of the entire membrane

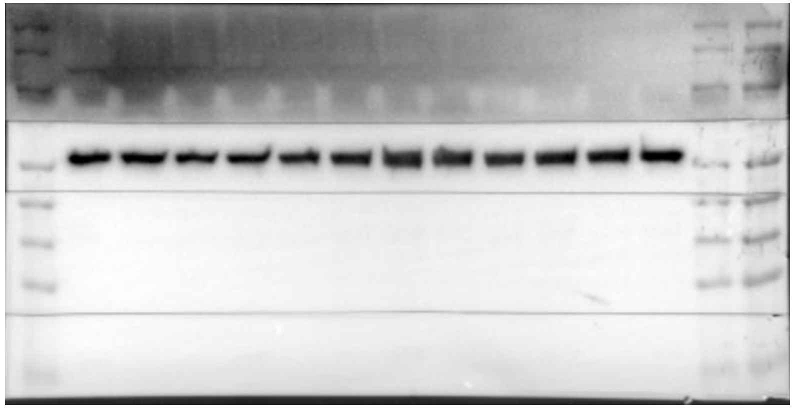

Original cropped image of the single membrane

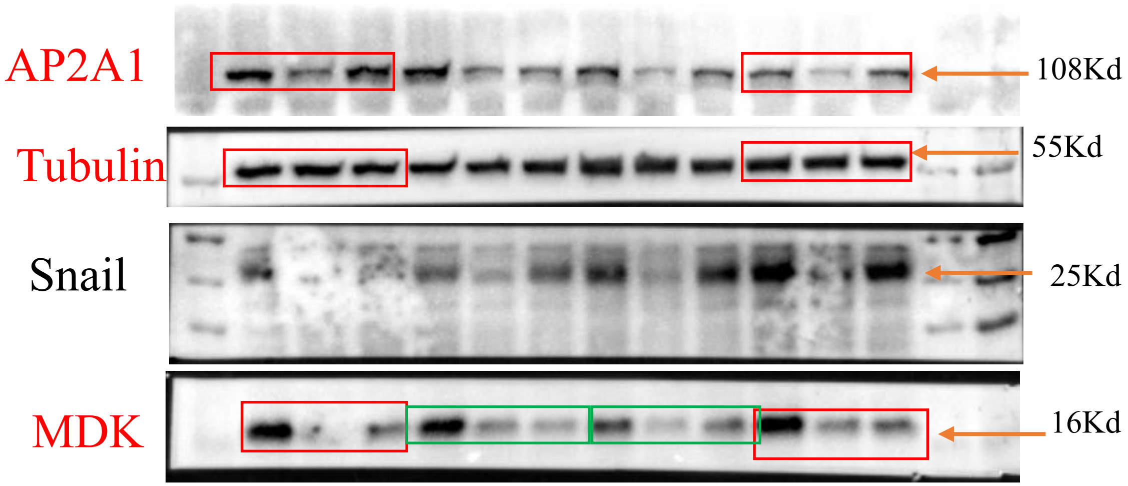

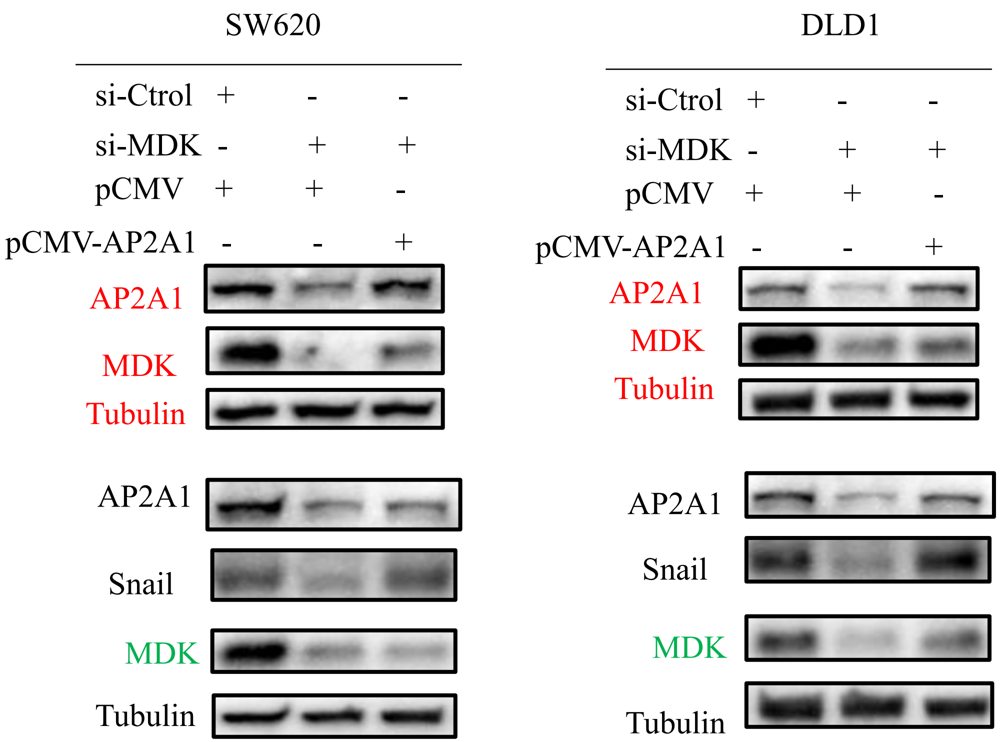

Figure 7E-MDK/AP2A1

Original uncropped image

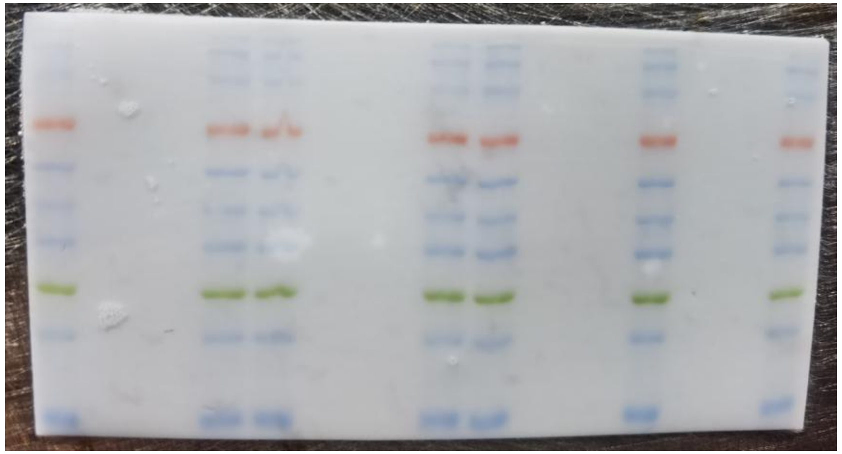

Original cropped image

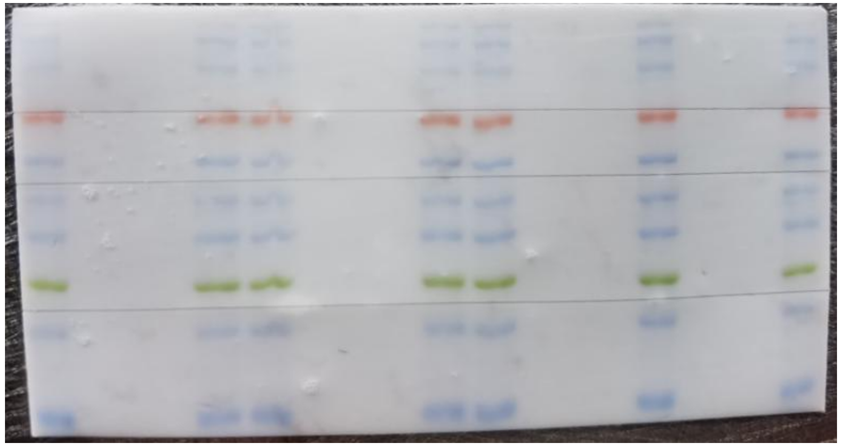

Original cropped image
of the entire membrane

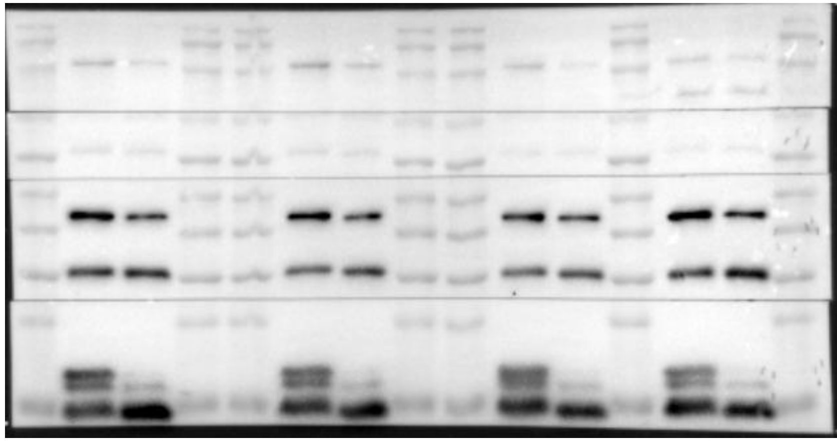

Original cropped image of the single membrane

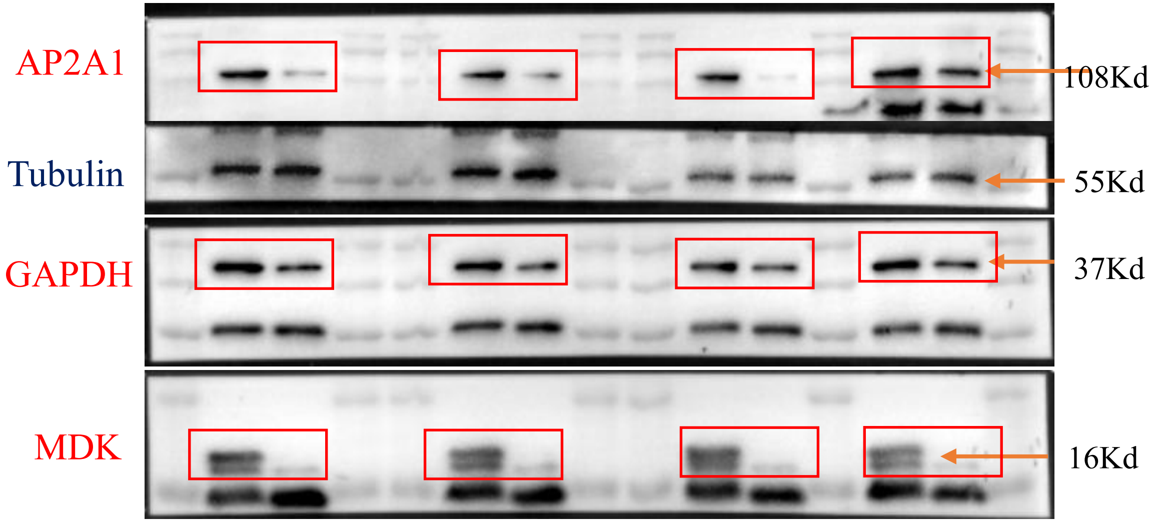

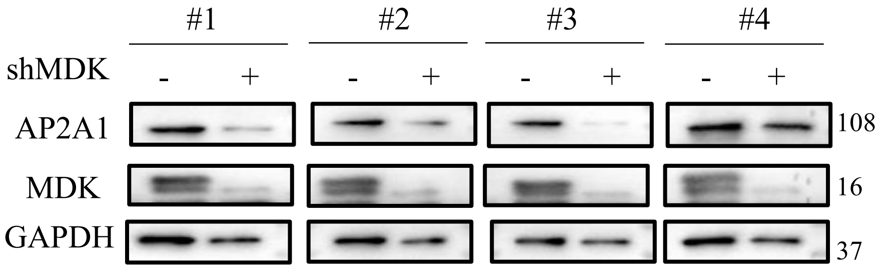

Figure 7E-MDK/AP2A1

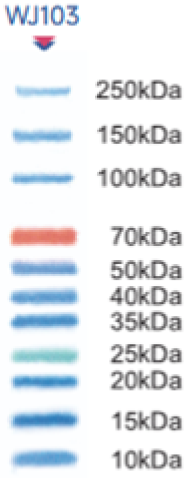

Figure 7E-MDK/AP2A1

Original uncropped image

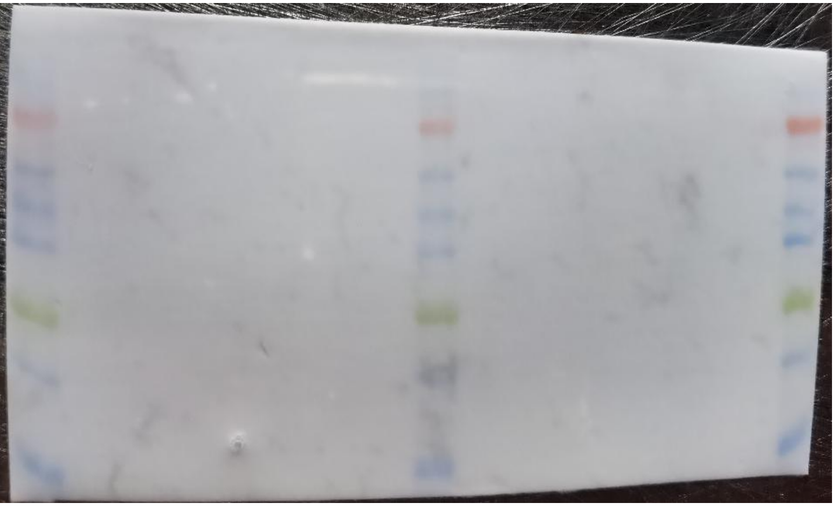

Original cropped image

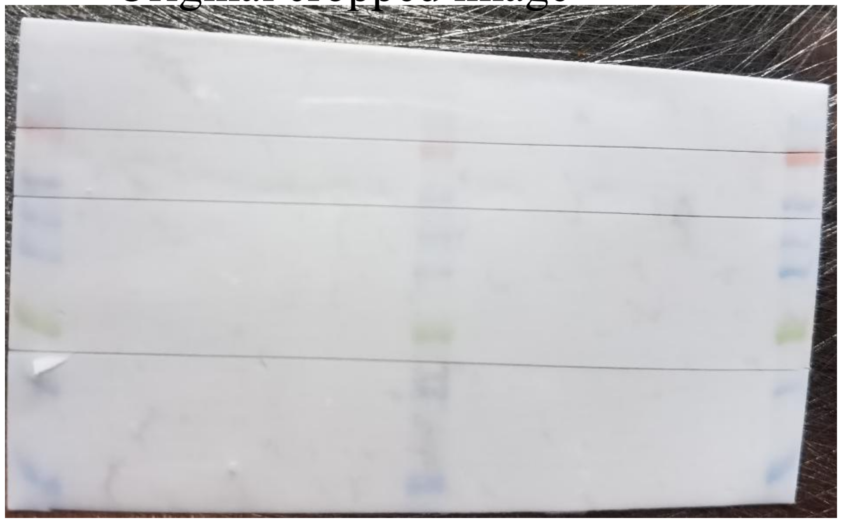

Original cropped image of the entire membrane

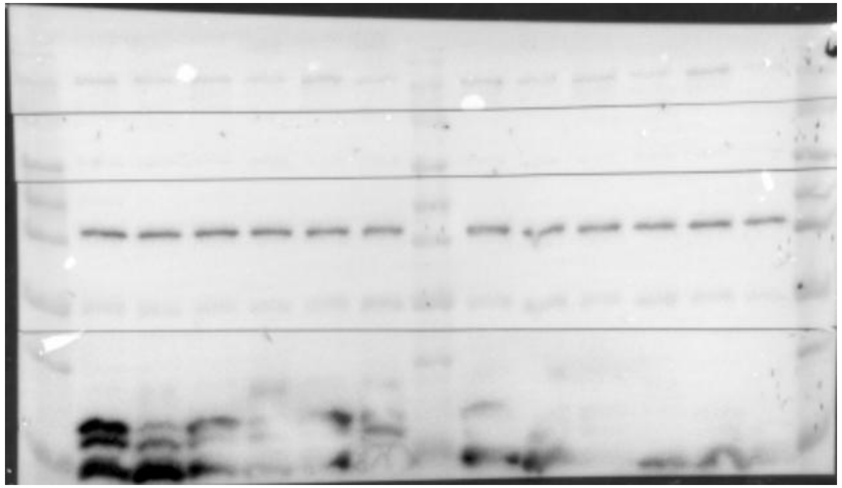

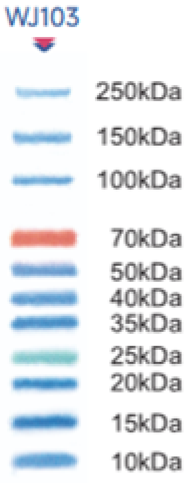

Original cropped image of the single membrane

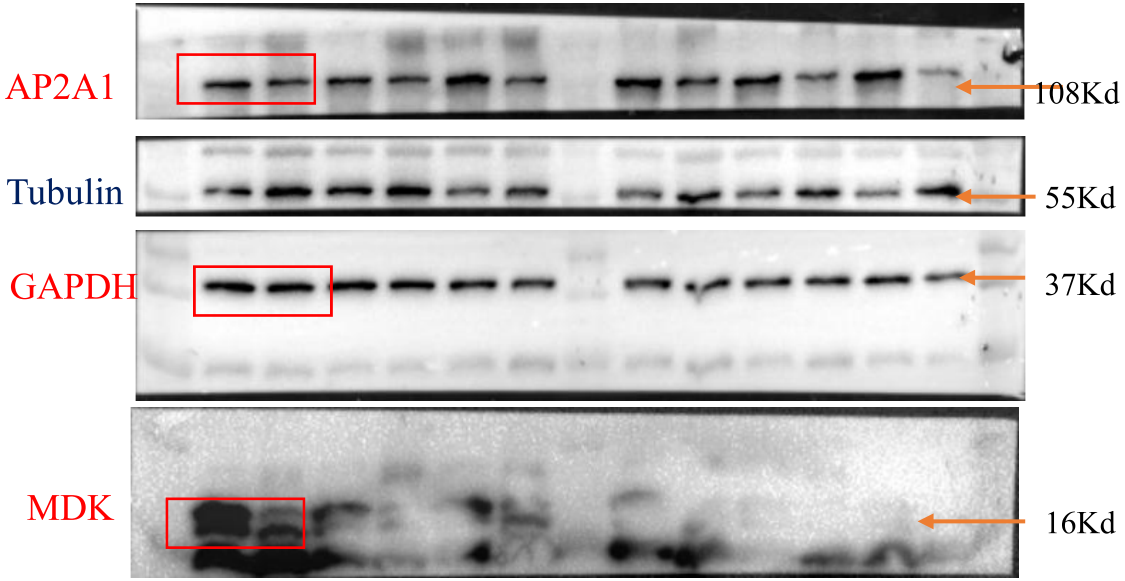

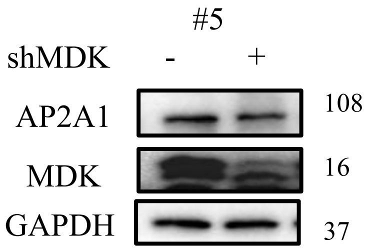

Figure 7E-MDK/AP2A1

Figure 8A

Original uncropped image

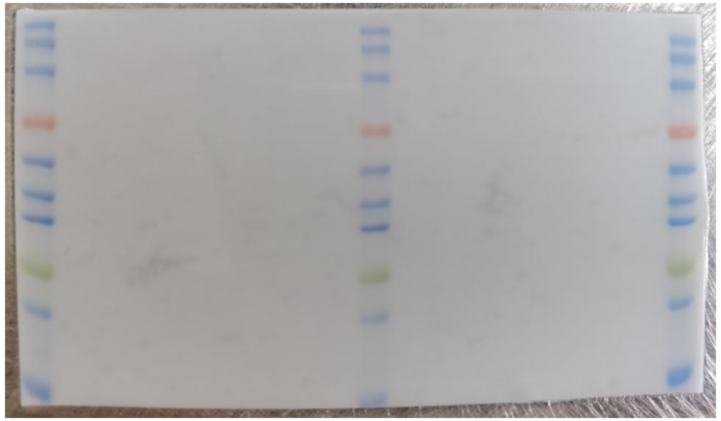

Original cropped image

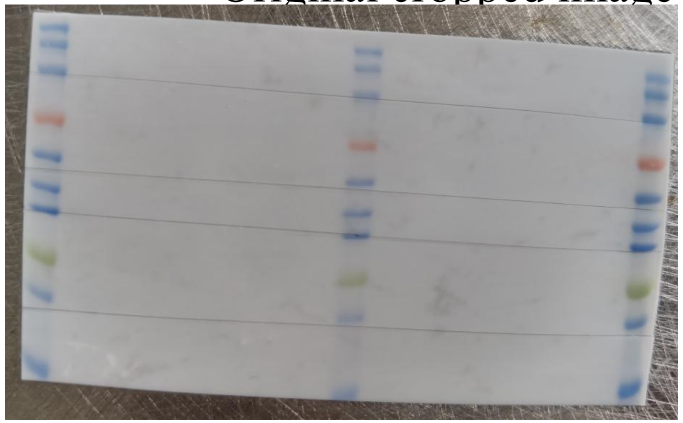

Original cropped image of the entire membrane

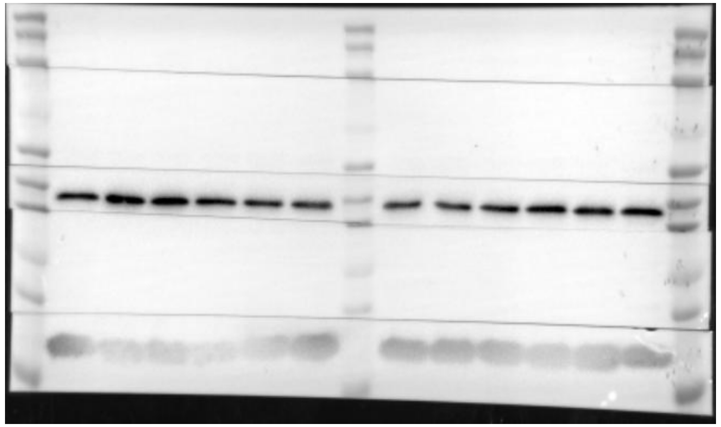

Original cropped image of the single membrane

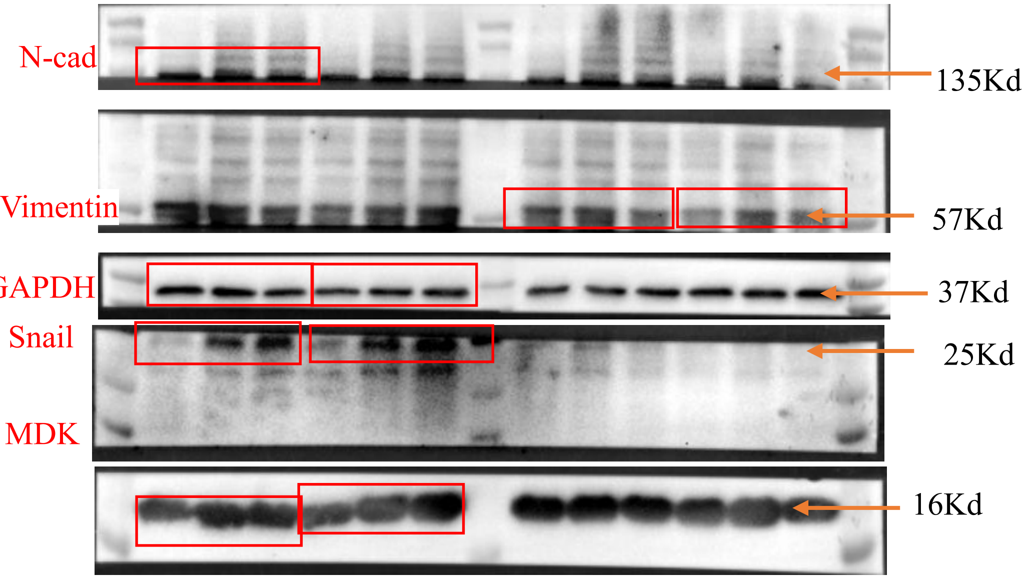

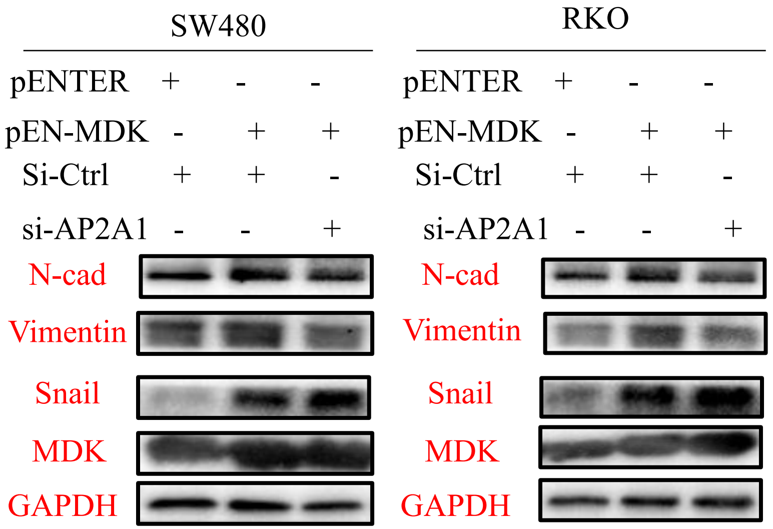

Figure 8B-Vimentin, 8C-AP2A1,GAPDH

Original uncropped image

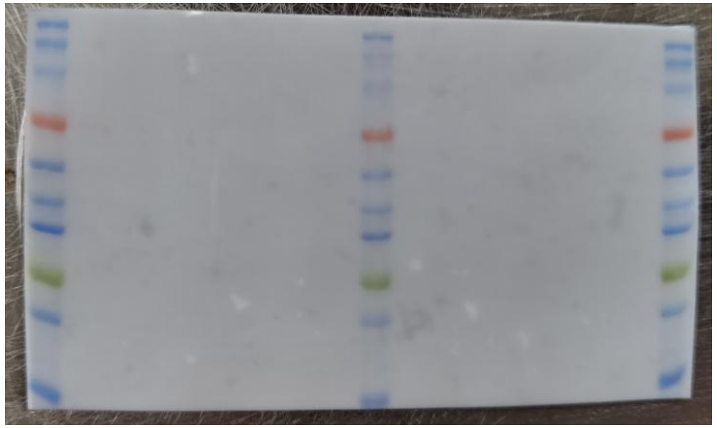

Original cropped image

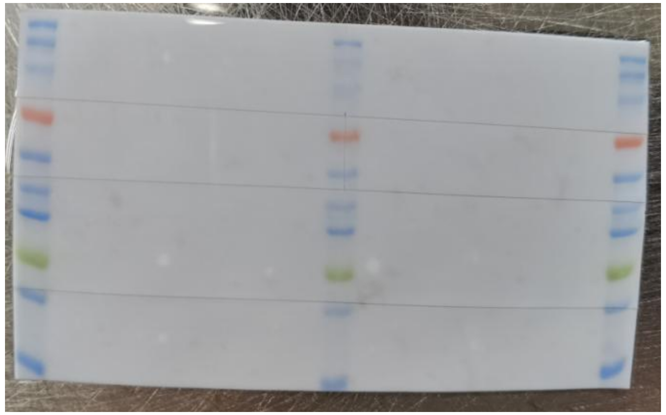

Original cropped image of the entire membrane

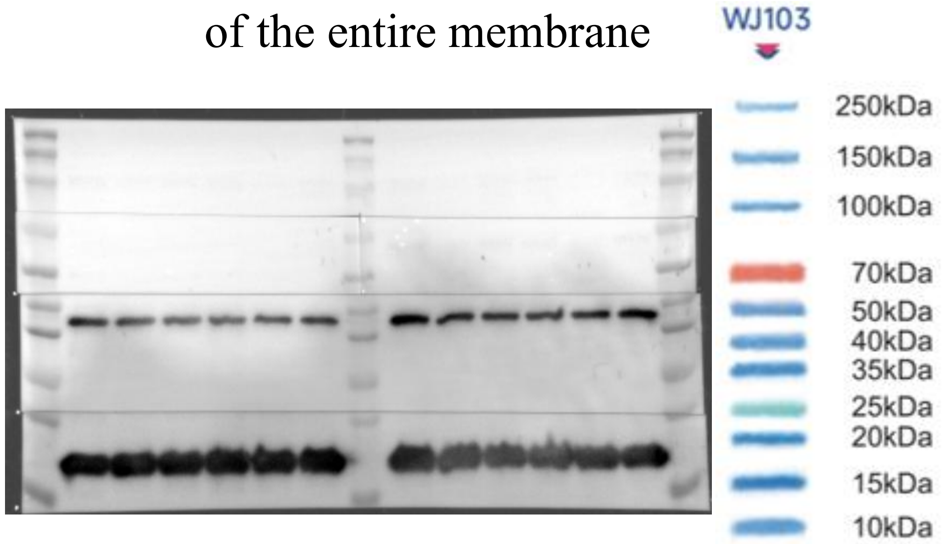

Original cropped image of the single membrane

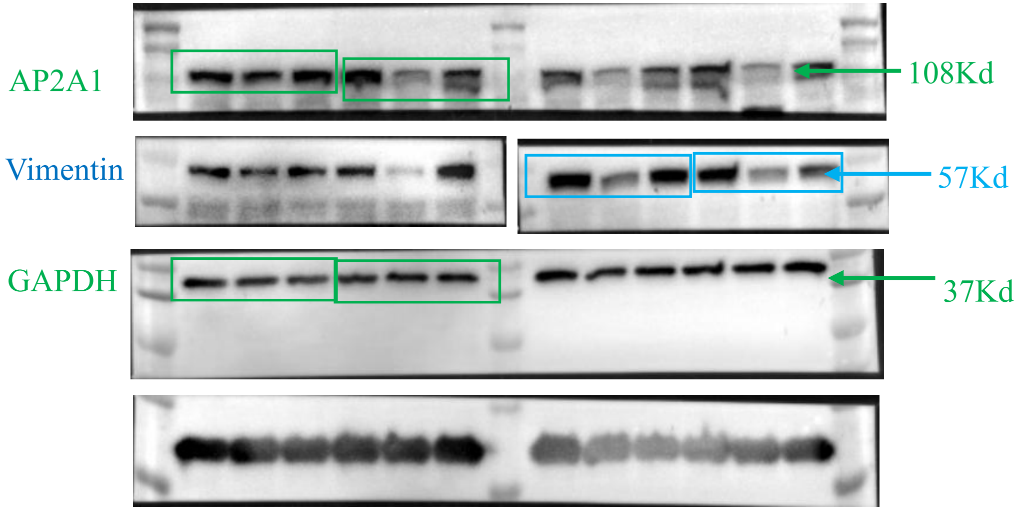

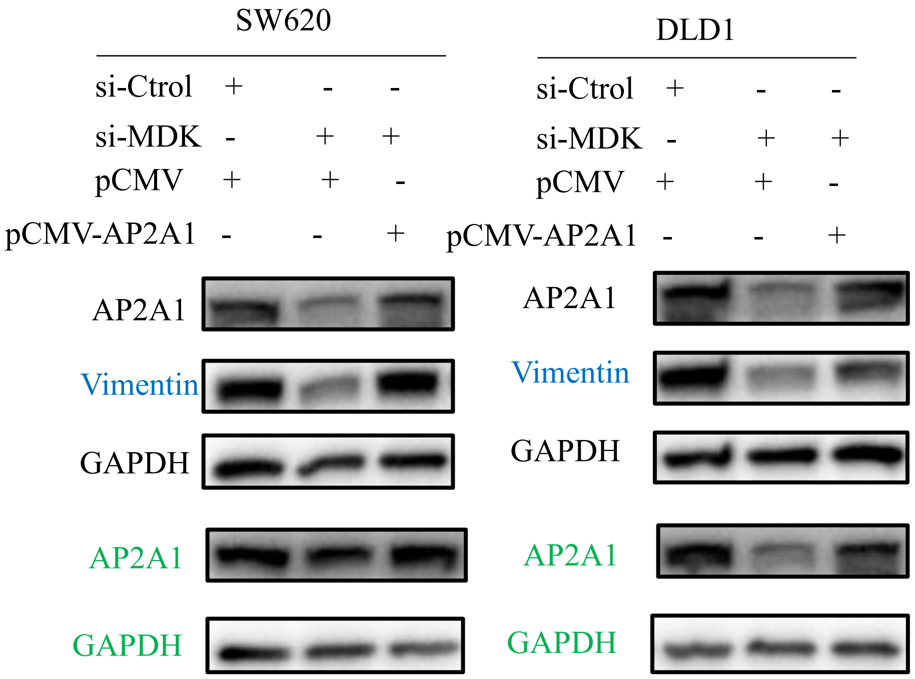

Figure 8B

Original uncropped image

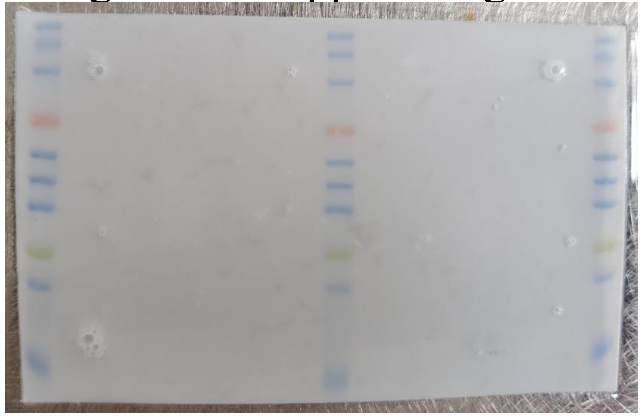

Original cropped image

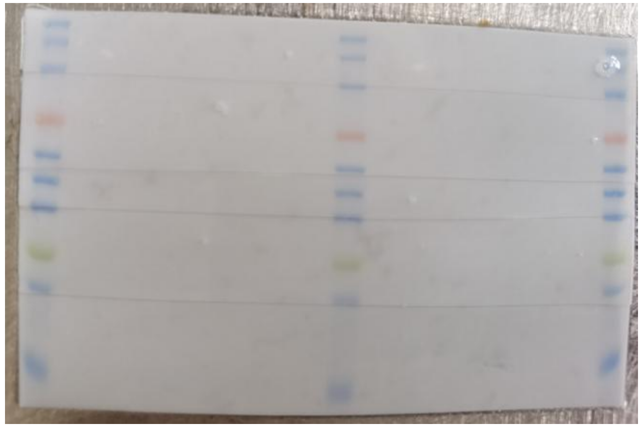

Original cropped image of the entire membrane

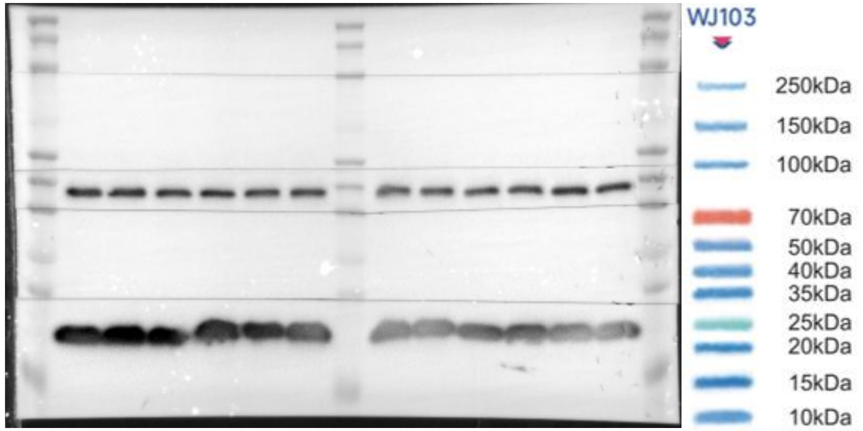

Original cropped image of the single membrane

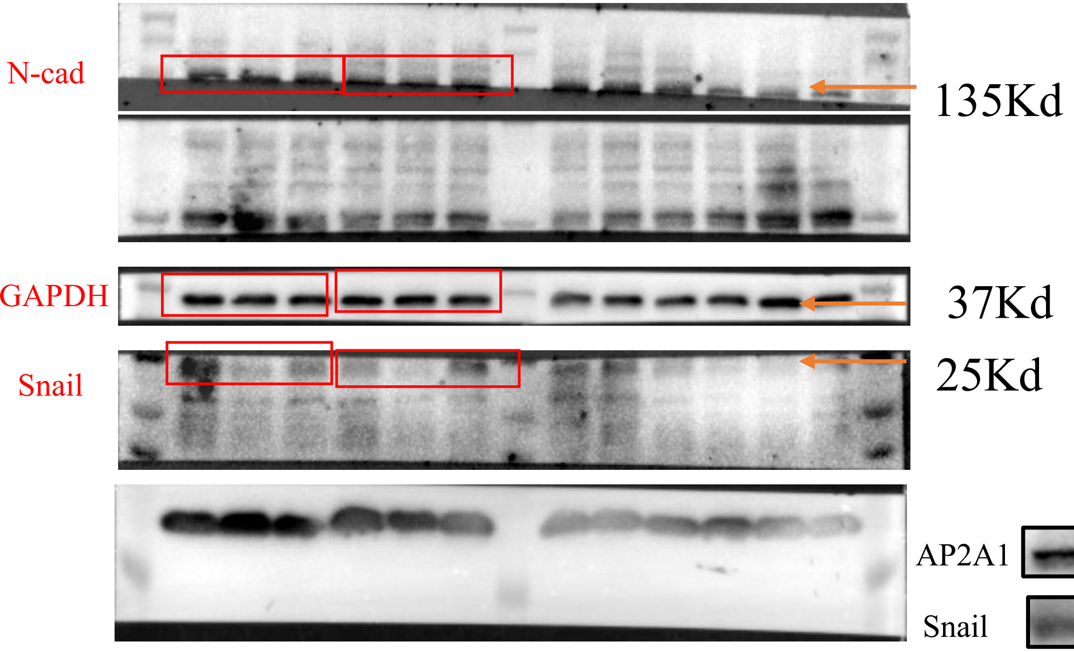

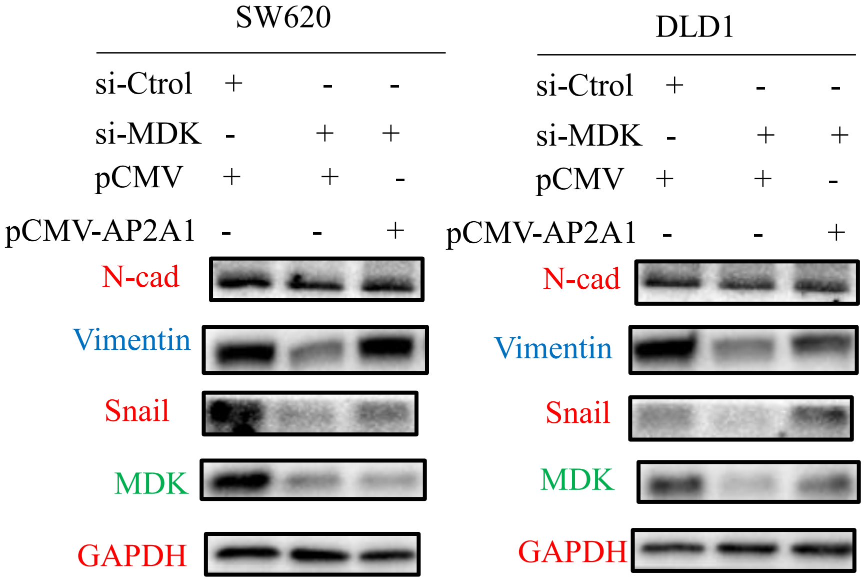

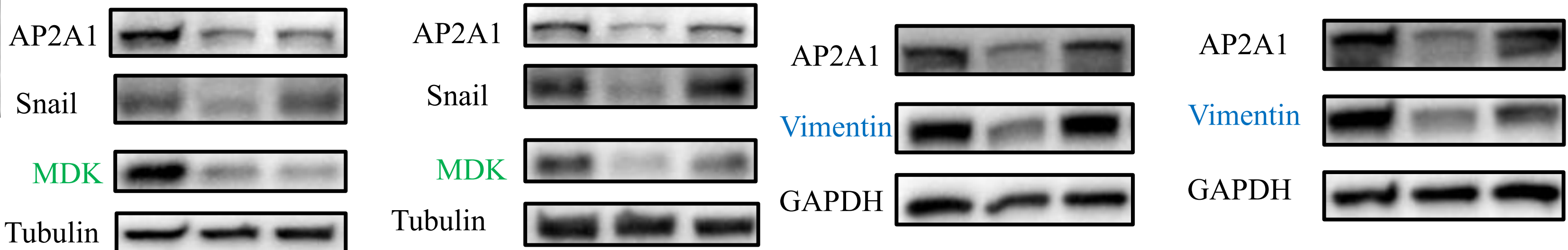

Figure 8C-p-PI3K

Original uncropped image

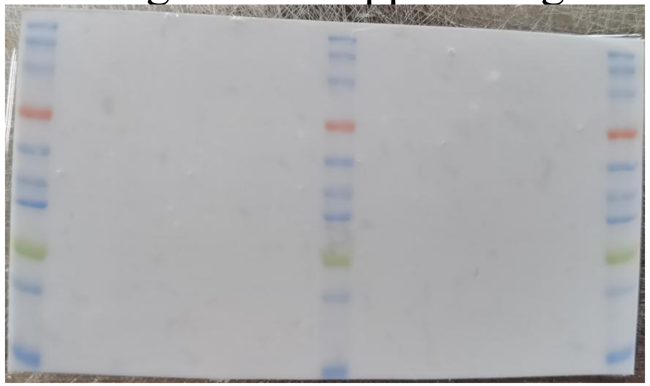

Original cropped image

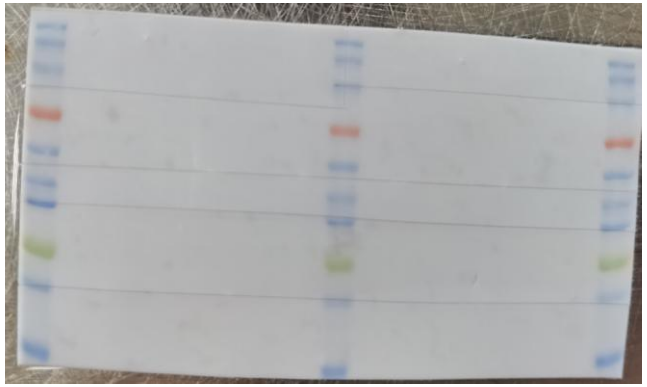

Original cropped image of the entire membrane

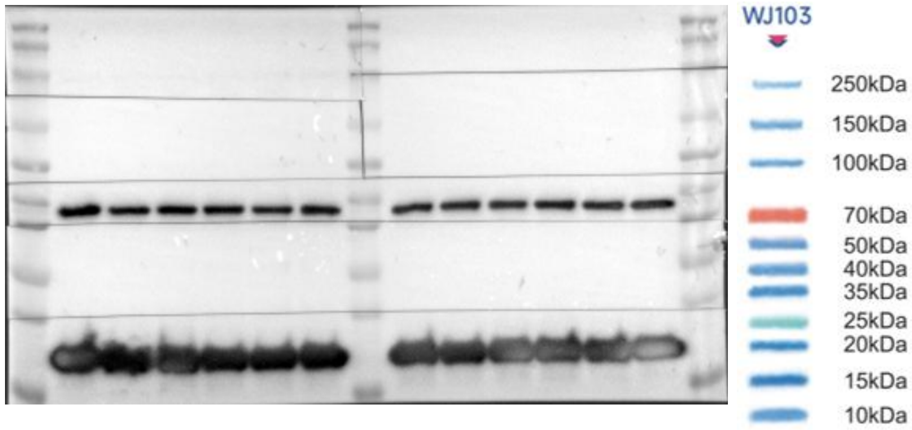

Original cropped image of the single membrane

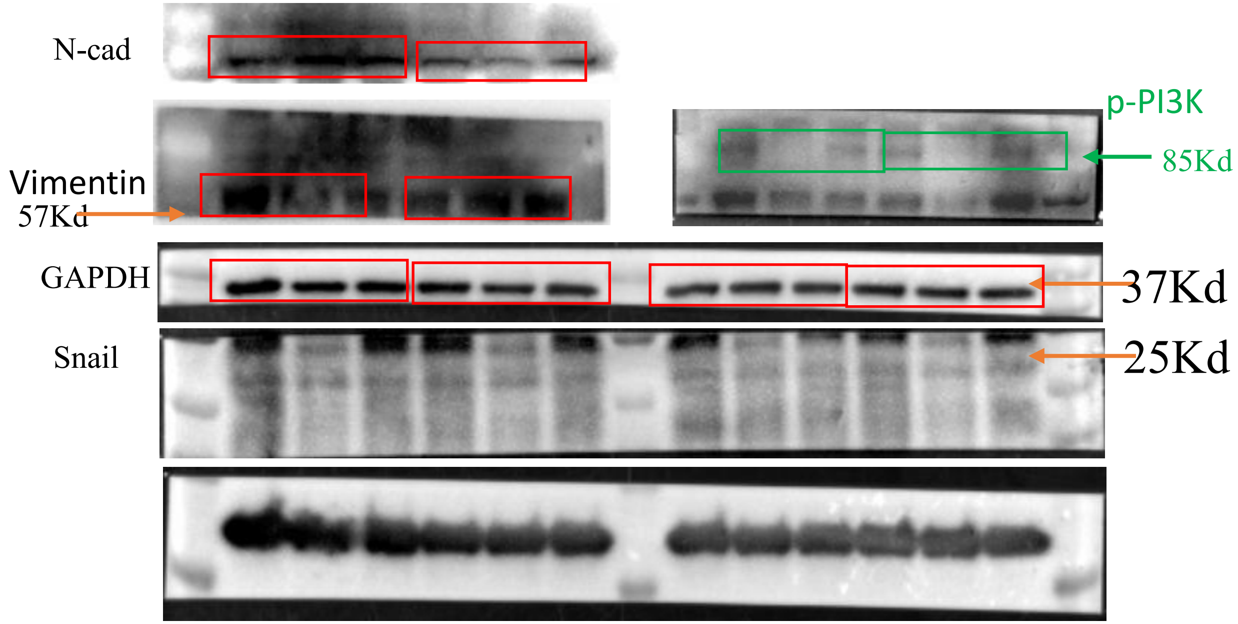

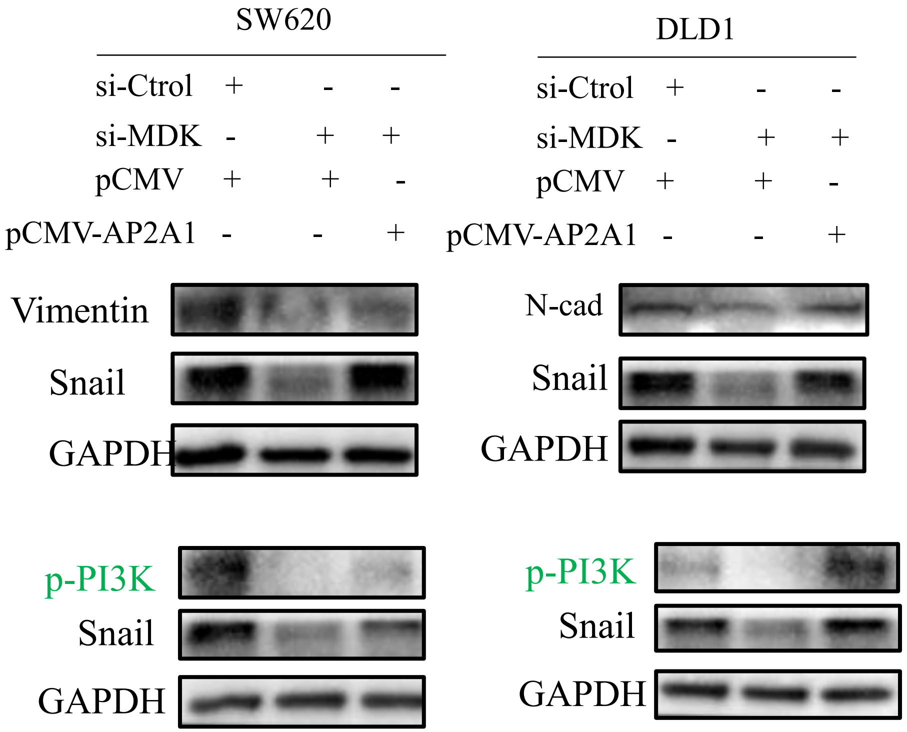

Figure 8C

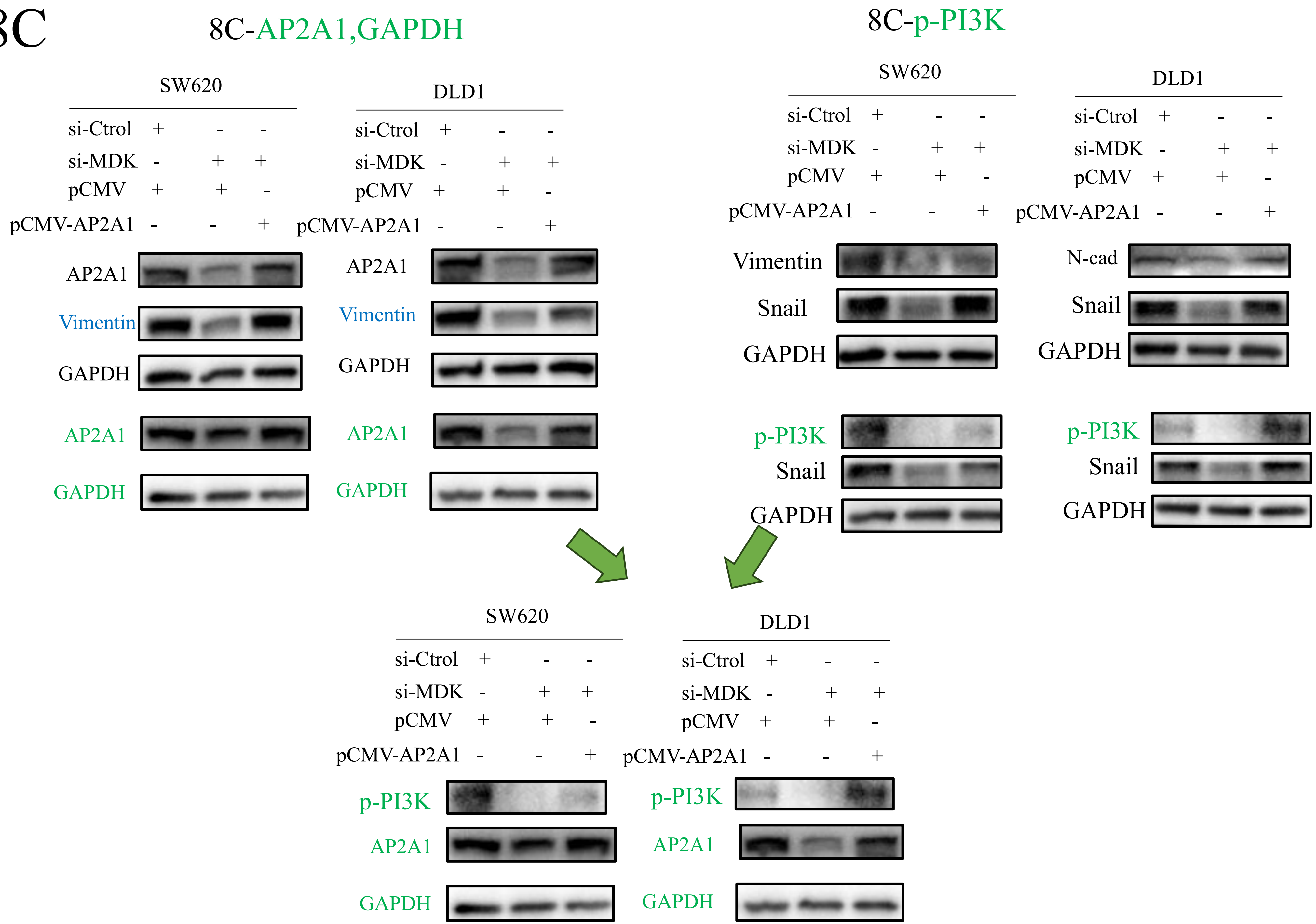
8C-AP2A1,GAPDH
8C-p-PI3K
SW620
DLD1
si-Ctrol
si-MDK
pCMV
pCMV-AP2A1
AP2A1
Vimentin
GAPDH
N-cad
Snail
p-PI3K

Figure 8D

Original uncropped image

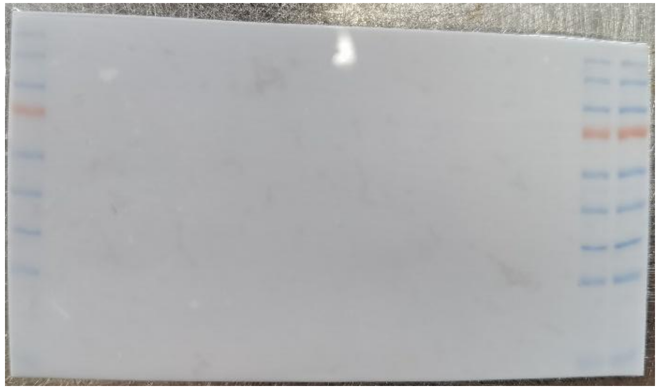

Original cropped image

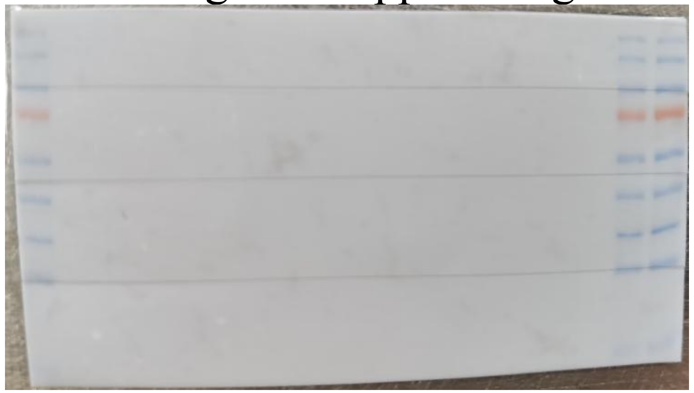

Original cropped image of the entire membrane

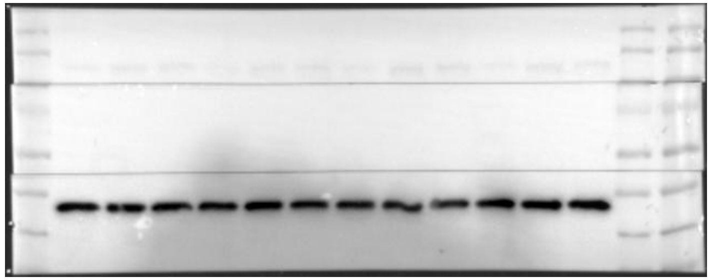

Original cropped image of the single membrane

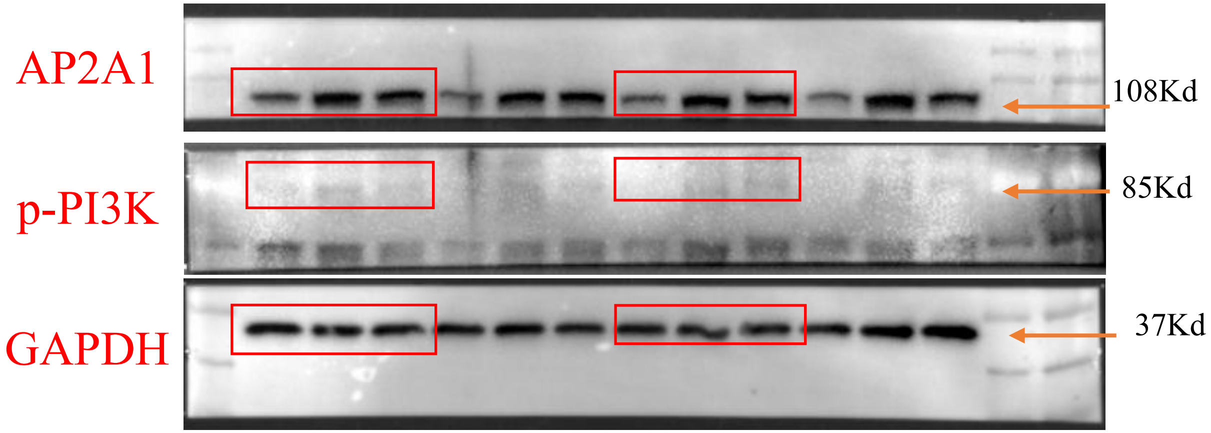

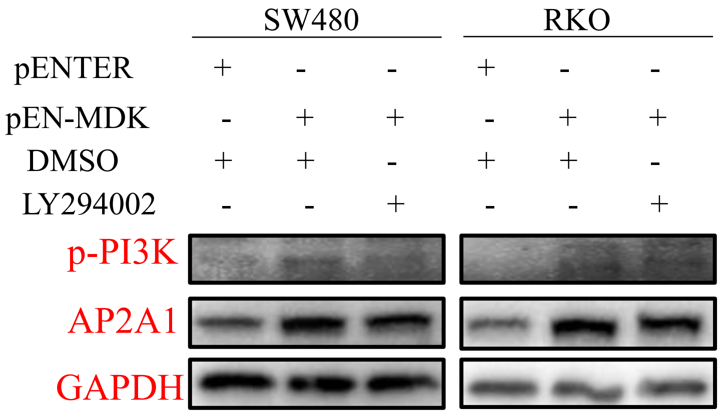

Supplement: Supplementary file 1 [file cancers-18-01311-s001.zip › File S1. full uncropped Gels and Blots images.pdf]
