# Supplementary material for: MDK Activates the PI3K/AKT Axis to Induce AP2A1 Expression and Epithelial–Mesenchymal Transition in Colorectal Cancer
Source: Cancers (Basel). 2026 Apr 21;18(8):1311. doi: 10.3390/cancers18081311 (PMC13114282; doi:10.3390/cancers18081311)
Supplement: Supplementary file 1 [file cancers-18-01311-s001.zip › Supplementary Figure S1-S5 and Tables S1-S6.pdf]

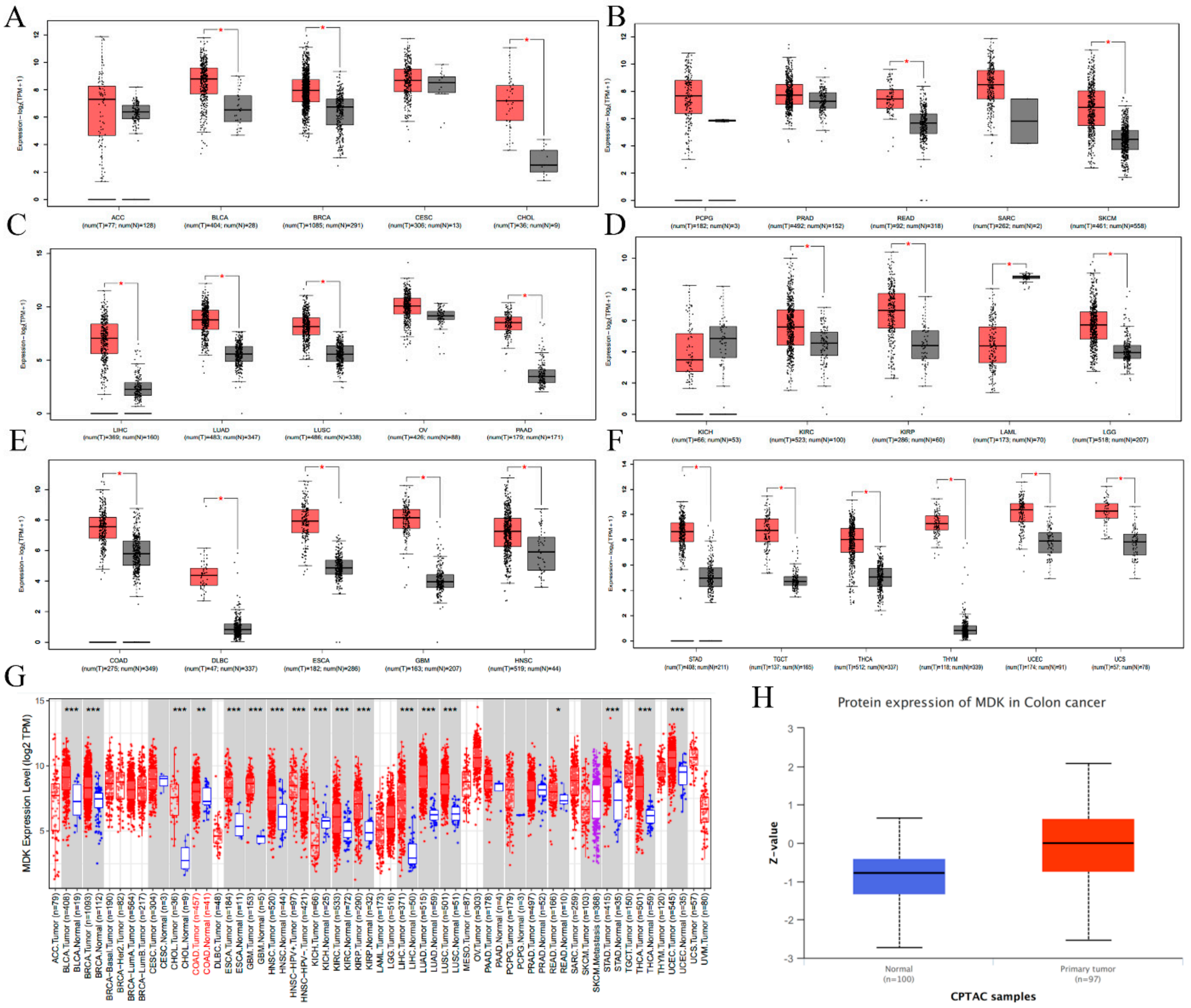

Figure S1. mRNA expression patterns of MDK in different types of human cancer. (A-F) The expression levels of MDK mRNA in tumor tissues and normal tissues were analyzed based on the GEPIA2 and (G) TIMER2 database. (H) Protein expression of MDK in Colon cancer in CPTAC samples.

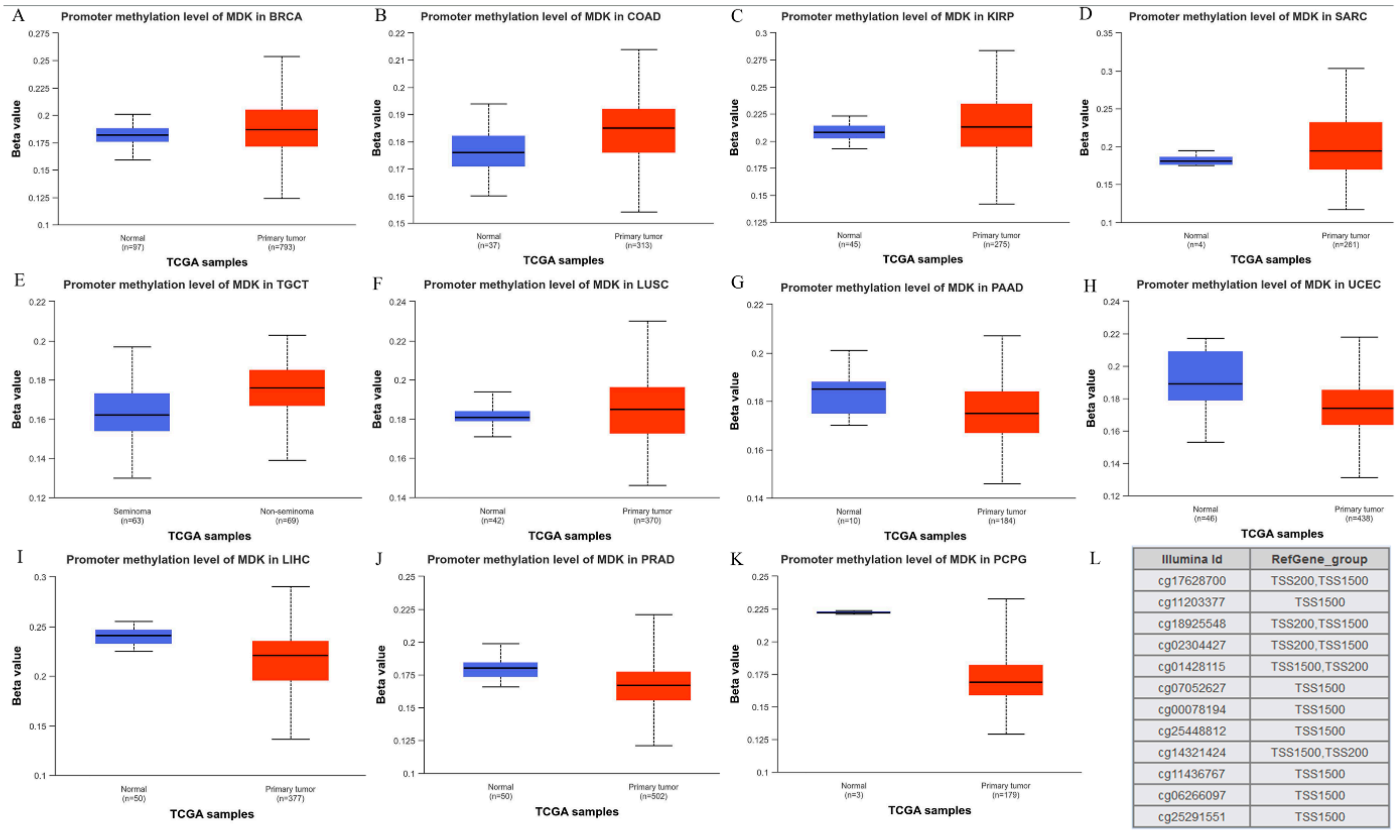

L

Illumina Id	RefGene_group
cg17628700	TSS200,TSS1500
cg11203377	TSS1500
cg18925548	TSS200,TSS1500
cg02304427	TSS200,TSS1500
cg01428115	TSS1500,TSS200
cg07052627	TSS1500
cg00078194	TSS1500
cg25448812	TSS1500
cg14321424	TSS1500,TSS200
cg11436767	TSS1500
cg06266097	TSS1500
cg25291551	TSS1500

Figure S2. DNA methylation aberration of MDK in tumors. (A-F) Six tumors with high MDK expression presented with hypomethylation in BRCA, COAD, KIRP, SARC, TCGT and LUSC respectively. (G-K) Five tumors with high MDK expression showed hypomethylation in PAAD, UCEC, LIHC, PRAD and PCPG. * $p< 0.05$. This data was obtained using Ualcan. (L) Probes for detecting DNA methylation of MDK promoter.

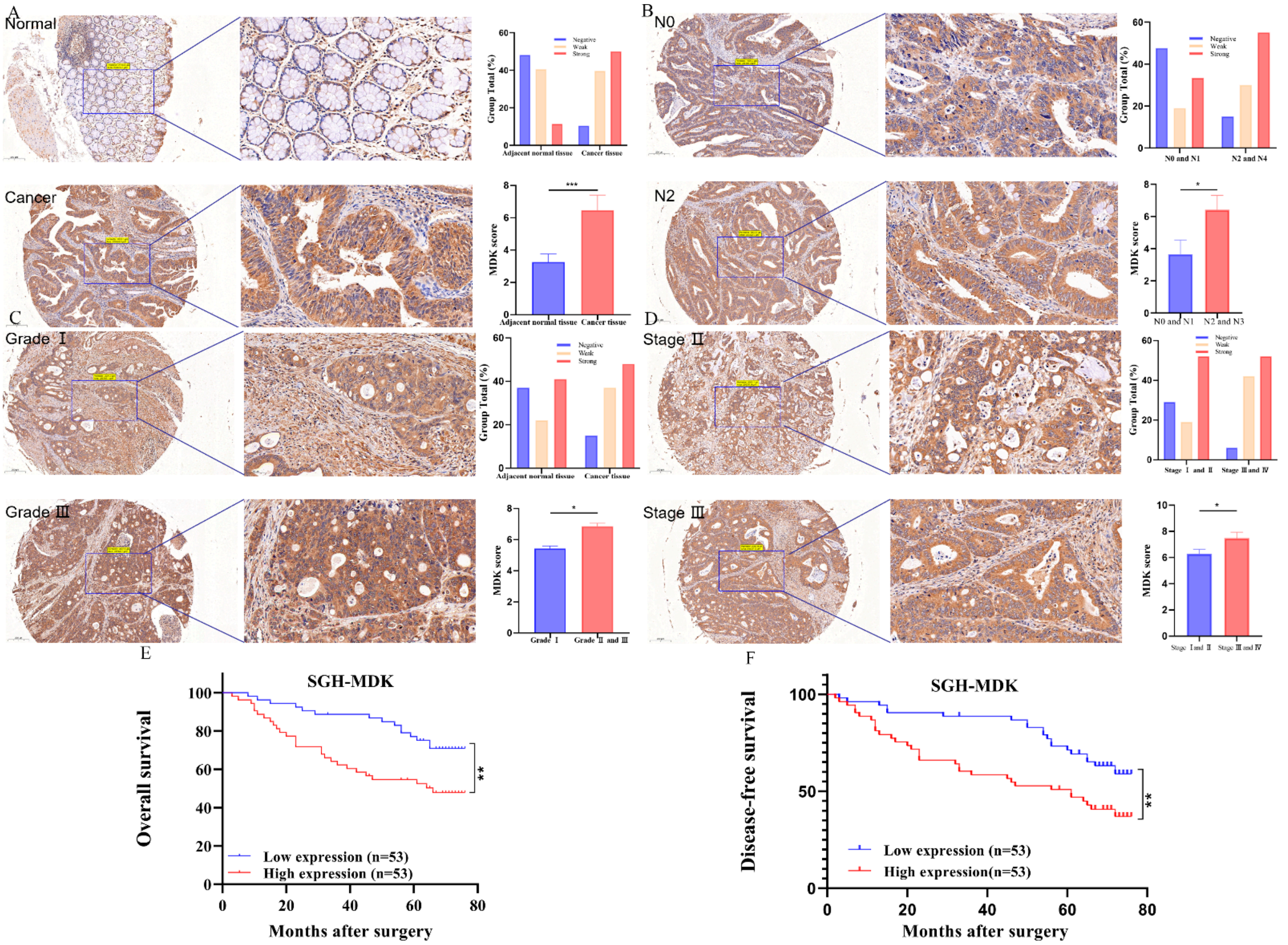

Figure S3. Overexpression of MDK indicates poor clinical outcome of colorectal cancer (CRC). (A) Representative images of MDK levels in CRC tissues and matched normal mucosa (magnification, 100× and 400×) from the tissue microarray (including 106 CRC tissues and matched normal tissues). (B-D) MDK expression between N0 and N1 vs N2 and N3 (B, P=0.001), grade I vs grade II and III (C, P=0.023), TNM stage I and II vs TNM stage III and IV (G, P < 0.001). (E-F) Kaplan-Meier survival analysis (log-rank test) showed that CRC patients with strong positive expression of MDK had a lower overall survival and disease free survival than those with negative and weak positive expression. $^{*}p < 0.05$, $^{**}p < 0.01$, $^{***}p < 0.001$.

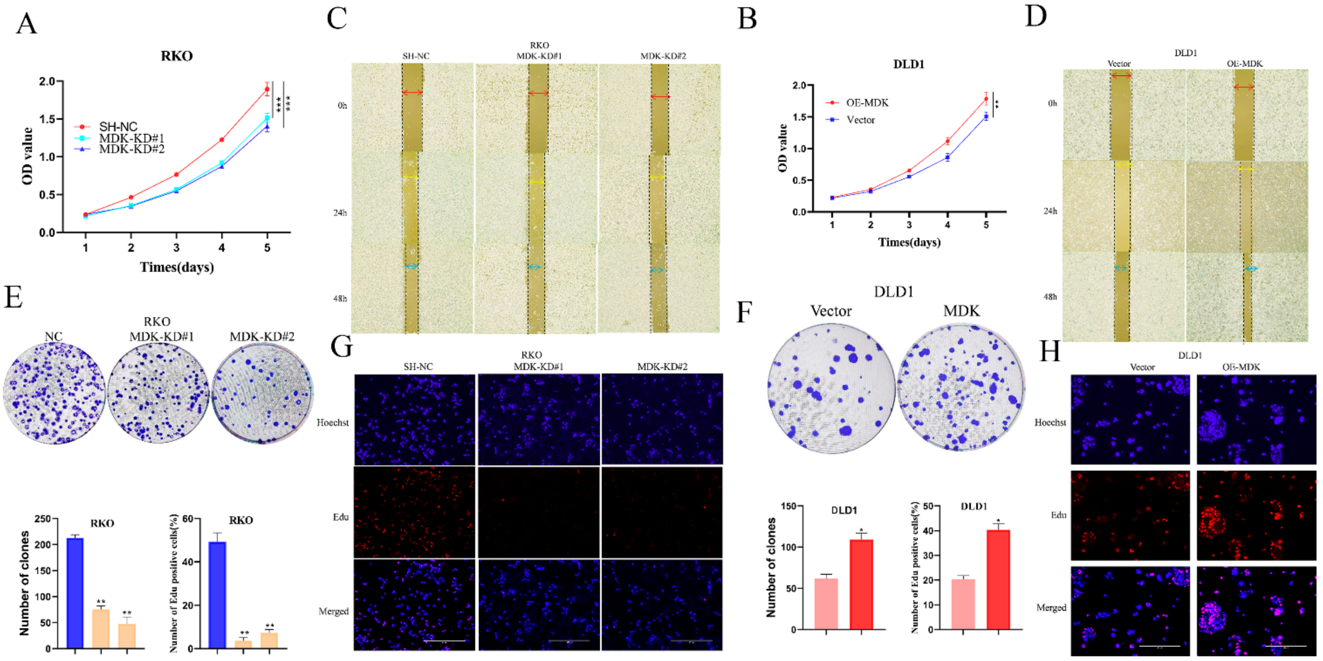

Figure S4. MDK promoted CRC cell proliferation and migration. (A) MDK downregulation inhibited the proliferation of RKO cell. (B) MDK upregulation promoted the proliferation of DLD1 cell. (C) MDK downregulation inhibited the migration of RKO cell. (D) MDK upregulation promoted the migration of DLD1 cell. (E) MDK downregulation weakened colony formation in RKO cell and the number of colones were calculated. (F) MDK upregulation enhanced colony formation in DLD1 cell and the number of colones were calculated. (G-H) Edu assays revealed the proliferation of RKO and DLD1 CRC cells. The representative results were showd. *p < 0.05, **p < 0.01, ***p < 0.001.

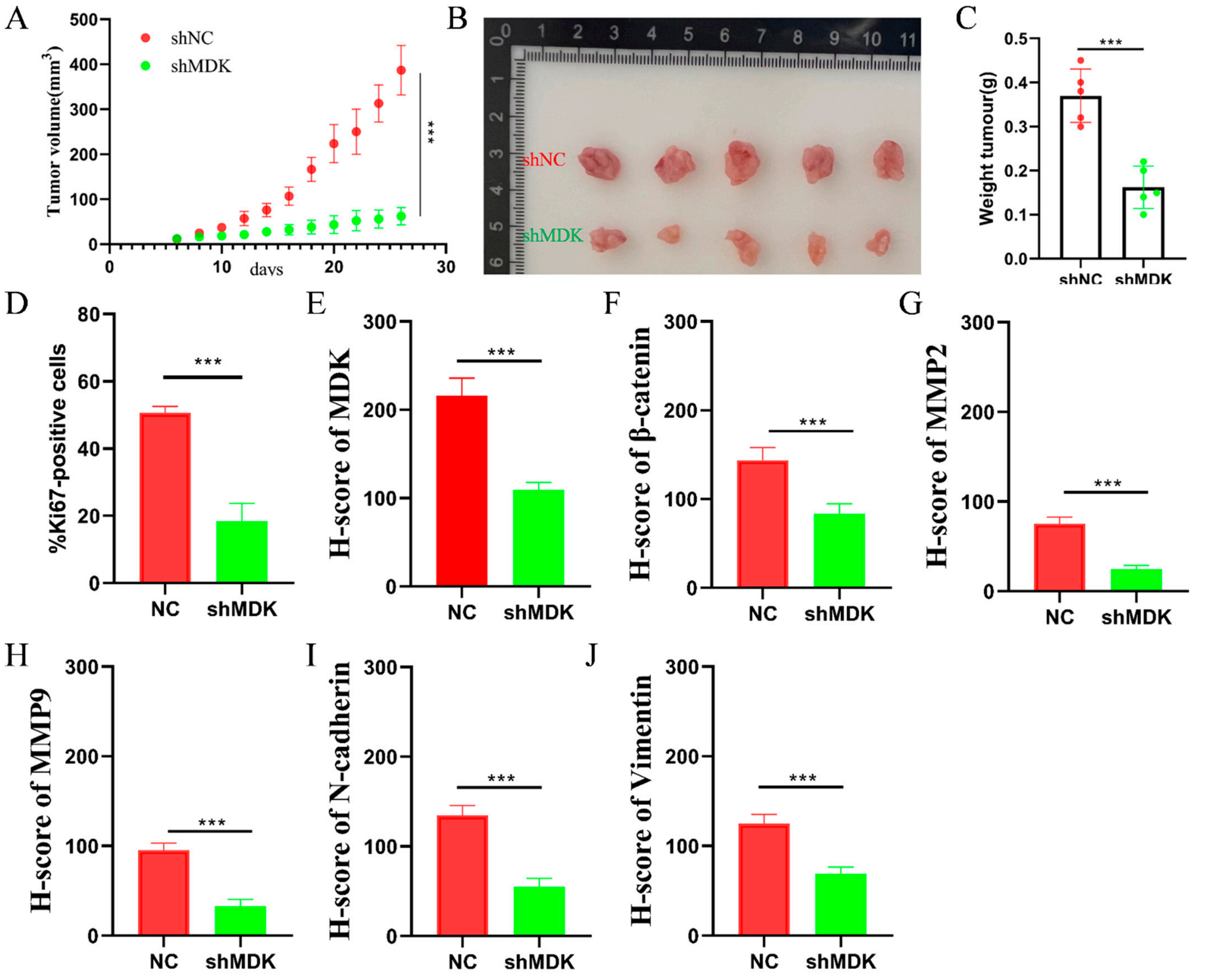

Figure S5. MDK knockdown inhibits tumor growth in vivo and quantification of immunohistochemical staining. (A) Tumor growth curves were plotted for nude mice subcutaneously injected with shMDK or shNC GC cells. (B, C) Representative images of dissected tumors (B) and quantitative analysis of tumor weight (C) are shown (mean ± SD, n=5). (D) Positive cell percentage of Ki-67 (E-J) H-score analysis of MDK, β-catenin, MMP2, MMP9, N-cadherin, and Vimentin. Data are presented as mean ± SD (n = 5 per group). **P < 0.01, ***P < 0.001 vs. shNC group (Student's t-test).

Table S1. Primers used in the study

Name	Sequence
GAPDH	Forward: 5′- TGACATCAAGAAGGTGGTGAAGCAG -3′<br>Reverse: 5′- GTGTCGCTGTTGAAGTCAGAGGAG -3′
MDK	Forward: 5′- AAGGAGTTTGGAGCCGACTG -3′<br>Reverse: 5′- CATTGTAGCGCGCCTTCTTC -3′
AP2A1	Forward: 5′- CACCGACCTCCAGGACTACA -3′<br>Reverse: 5′- CAGTCTCCAGACATTCCACCA -3′

Table S2. Details of antibodies used in this study.

Antibody	Catalog No.	Source	Dilution or amount
GAPDH	60004-1-Ig	Proteintech	WB/1:50000
MDK	AB52637	Abcam	WB/1:1000
AP2A1	9887-1-AP	Proteintech	WB/1:1000
β-catenin	10153	BD biosciences	WB/1:1000; IHC/1:100
MMP-2	YT2798	Immunoway	WB/1:1000
MMP-9	YT1892	Immunoway	WB/1:1000
Vimentin	455134	Abmart	WB/1:1000
N-cadherin	22018-1-AP	Proteintech	WB/1:1000
Snail	3879	CST	WB/1:1000
PI3K	40115	Abmart	WB/1:1000
p-PI3K	40116	Abmart	WB/1:1000
ERK	4695	CST	WB/1:1000
p-ERK	4370	CST	WB/1:1000
AKT	4691	CST	WB/1:1000
p-AKT	4060	CST	WB/1:2000
MDK	11009-1-AP	Proteintech	IHC/1:50
Ki-67	Ab15580	Abcam	IHC/1:50
MMP-2	40994	CST	IHC/1:50
MMP-9	2270	CST	IHC/1:50
N-cadherin	13116	CST	IHC/1:50
Vimentin	5741	CST	IHC/1:50

WB: Western blot;

IHC: Immunohistochemistry.

Table S3. The siRNA sequences used in this study.

siRNA	sense (5′-3′)	antisense (5′-3′)
NC	UUCUCCGAACGUGUCACGUTT	ACGUGACACGUUCGGAGAATT
siMDK	UGUCUGCUCGUUAGCUUUAAUTT	AUUAAAGCUAACGAGCAGACATT
SiAP2A1	CCUCAUCAACAACGCCAUCAATT	UUGAUGGCGUUGUUGAUGAGGTT

Table S4. Clinicopathological characteristics in relation to MDK expression status in SGH cohort.

Characteristics	SGH cohort(N=106)	MDK expression High N=53(%)	MDK expression Low N=53(%)	χ2	P value
**Age**					
<65 y	51	26(51)	25(49)	0.038	0.846
>=65y	55	27(49.1)	28(50.9)		
**Gender**					
male	55	26(47.3)	29(52.7)	0.340	0.560
female	51	27(52.9)	24(47.1)		
**Differentiation**					
Well	19	5(26.3)	14(73.7)	5.194	**0.023**
Moderate +poor	87	48(55.2)	39(4.8)		
**T**					
T1-T2	12	4(33.3)	8(66.7)	1.504	0.220
T3-T4	94	49(52.1)	45(47.9)		
**N**					
N0+N1	82	34(41.5)	48(58.5)	10.557	**0.001**
N2+N3	24	19(79.2)	5(20.8)		
**Tumor size,cm**					
<5	59	30(50.8)	29(49.2))	1.226	0.542
>=5	47	23(48.9)	24(51.1)		
**Pathologic Stage**					
stage I-II	51	14(27.5)	37(72.5)	19.991	**< 0.001**
stage III-IV	55	39(70.9)	16(29.1		
**Status**					
Alive	64	26(40.6)	38(59.4)	5.679	**0.017**
Dead	42	27(64.3)	15(35.7)		

Statistical significance was determined by Chi-square test or Fisher's exact test. Bold values indicate statistical significance ($p < 0.05$).

Table S5. Univariate and multivariate analysis for overall survival (OS) in patients with CRC

Characteristics	No.	OS				DFS			
		Univariate analysis		multivariate analysis		Univariate analysis		multivariate analysis	
		$\chi2$	p	$\chi2$	p	$\chi2$	p	$\chi2$	p
**Age**									
< 65 y	51	3.633	0.057			3.707	0.054		
> =65y	55								
**Gender**									
male	55	0.068	0.794			0.283	0.595		
female	51								
**Differentiation**									
Well	19	1.1645	0.204			2.375	0.123		
Moderate +poor	87								
**T**									
T1-T2	12	1.132	0.287			2.286	0.131		
T3-T4	94								
**N**									
N0+N1	82	3.082	0.097			3.585	0.058		
N2+N3	24								
**Tumor size,cm**									
<5	59	0.076	0.783			0.029	0.866		
>=5	47								
**Pathologic Stage**									
stage I-II	51	5.449	**0.020**			5.549	**0.019**		
stage III-IV	55								
**MDK expression**									
High	53	7.193	**0.007**	1.979	**0.044**	5.921	**0.009**	1.186	**0.046**
Low	53								

Statistical significance was determined by Chi-square test or Fisher's exact test. Bold values indicate statistical significance ($p < 0.05$). -

Table S6. Clinicopathological characteristics in relation to MDK expression status in TCGA cohort.

Characteristics	TCGA cohort(N=438)	MDK expression High N=284(%)	MDK expression Low N=154(%)	χ2	P value
**Age**					
<65 y		108(63.9)	61(36.1)	0.105	0.745
>=65y		176(65.4)	93(34.6)		
**Gender**					
male		149(63.9)	84(36.1)	0.174	0.667
female		135(65.9)	70(34.1)		
**Race**					
ASIAN		6(54.5)	5(45.5)		
BLACK or AFRICAN AMERICAN		34(58.6)	24(41.4)		
WHITE		122(58.1)	88(41.9)	15.784	**0.003**
AMERICAN INDIAN or ALASKA NATIVE		1(100)	0(0)		
NA		121(68.6)	37(31.4)		
**T**					
T1-T2		56(64.4)	31(35.6)	0.011	0.918
T3-T4		228(65)	123(35)		
**N**					
N0+N1		235(62.5)	135(37.5)	4.856	**0.028**
N2+N3		59(75.6)	19(24.4)		
**M**					
M0		214(64.8)	116(35.2)		
M1		44(72.1)	17(27.9)	3.291	0.193
MX		26(65.5)	21(34.5)		
**Pathologic Stage**					
stage I-II		150(61.5)	94(38.5)	2.744	0.254
stage III-IV		132(69.1)	59(30.9)		
NA		2(66.7)	1(33.3)		
**Status**					
Alive		2111(62.1)	129(37.9)	5.156	**0.023**
Dead		73(74.5)	25(25.5)		

Statistical significance was determined by Chi-square test or Fisher's exact test. Bold values indicate statistical significance ($p < 0.05$).
