# Supplementary figures and images for: MDK Activates the PI3K/AKT Axis to Induce AP2A1 Expression and Epithelial–Mesenchymal Transition in Colorectal Cancer
Source: Cancers (Basel). 2026 Apr 21;18(8):1311. doi: 10.3390/cancers18081311 (PMC13114282; doi:10.3390/cancers18081311)

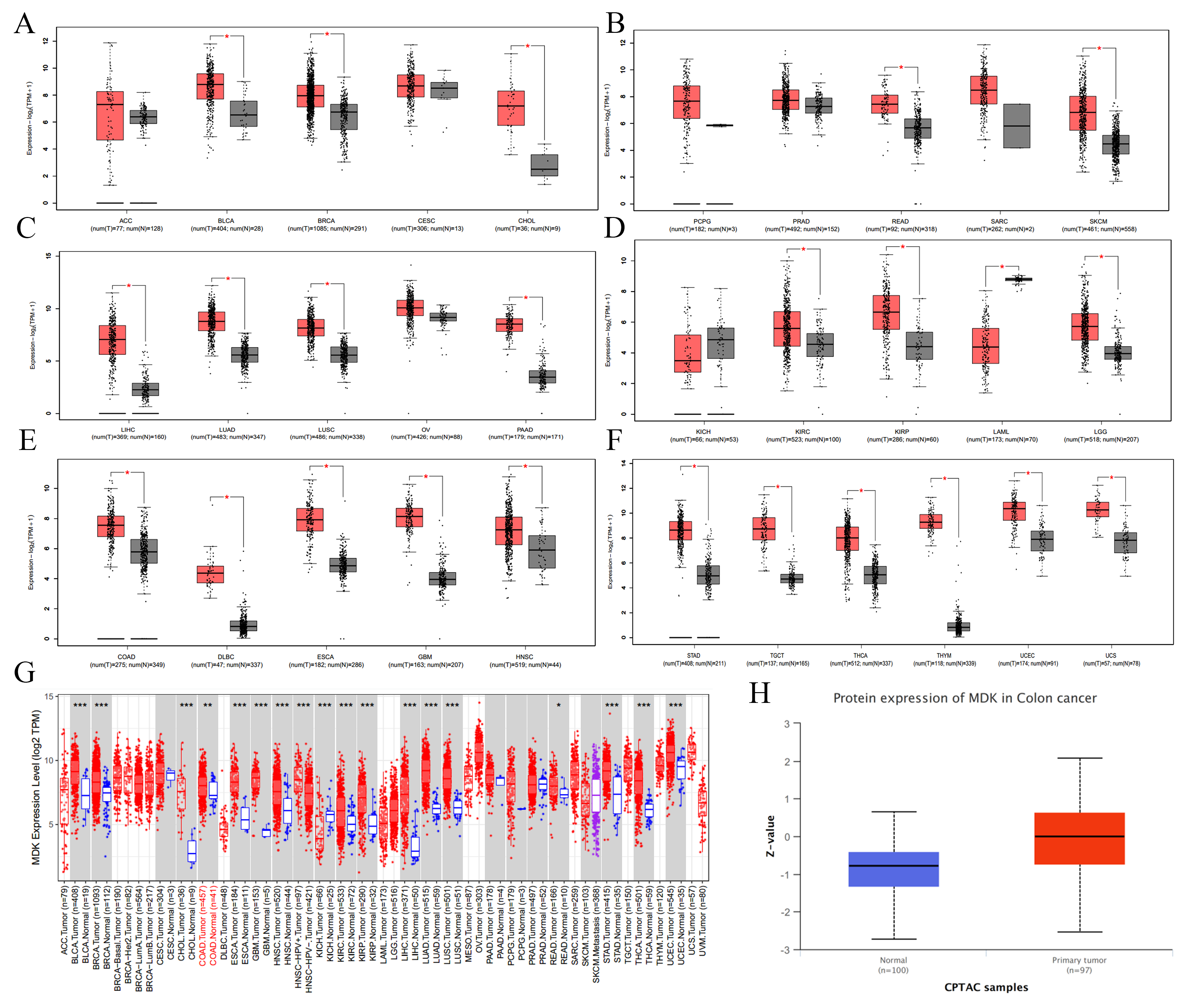

Supplement: Supplementary file 1 [file cancers-18-01311-s001.zip › Supplementary Figure S1.tif]

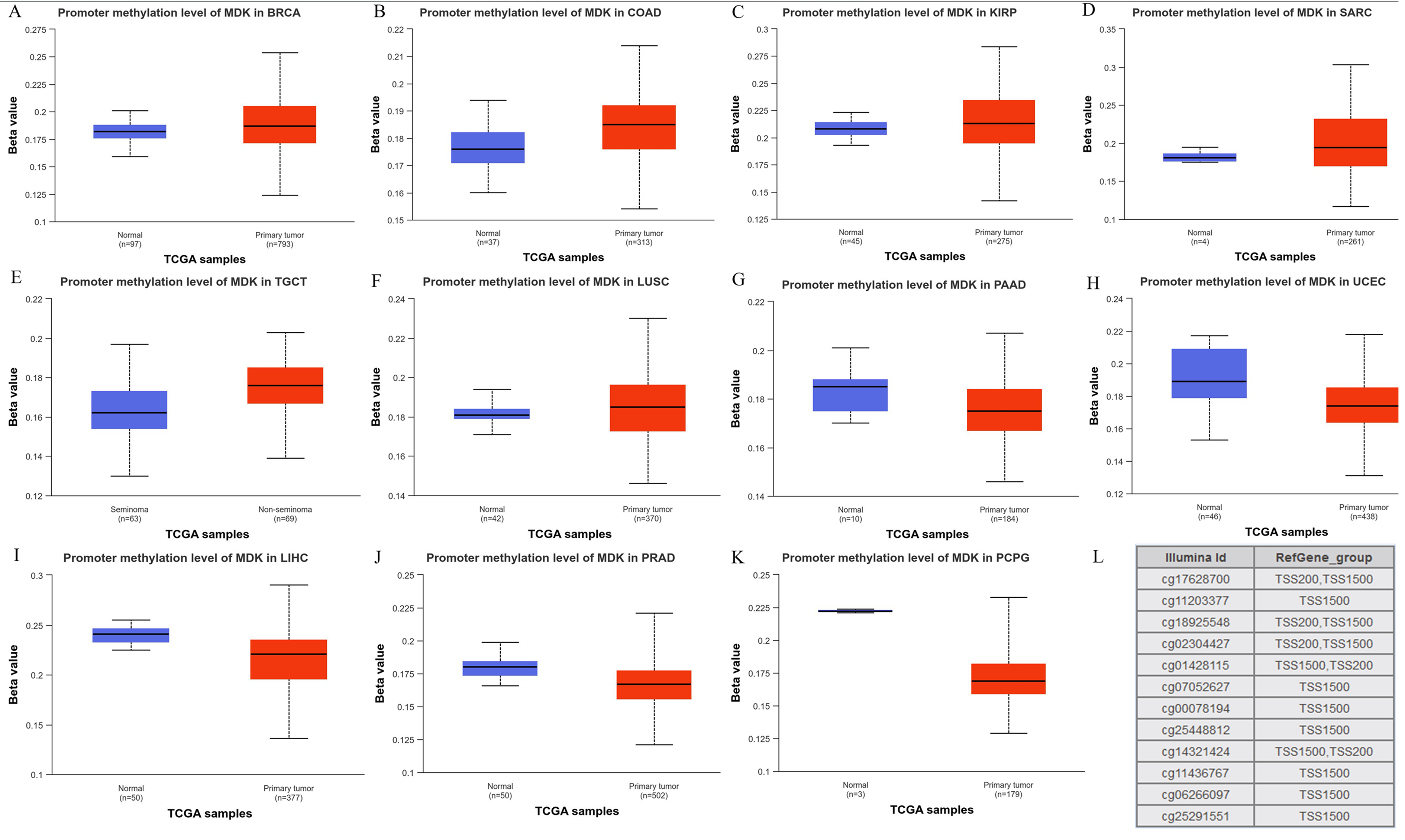

Supplement: Supplementary file 1 [file cancers-18-01311-s001.zip › Supplementary Figure S2.tif]

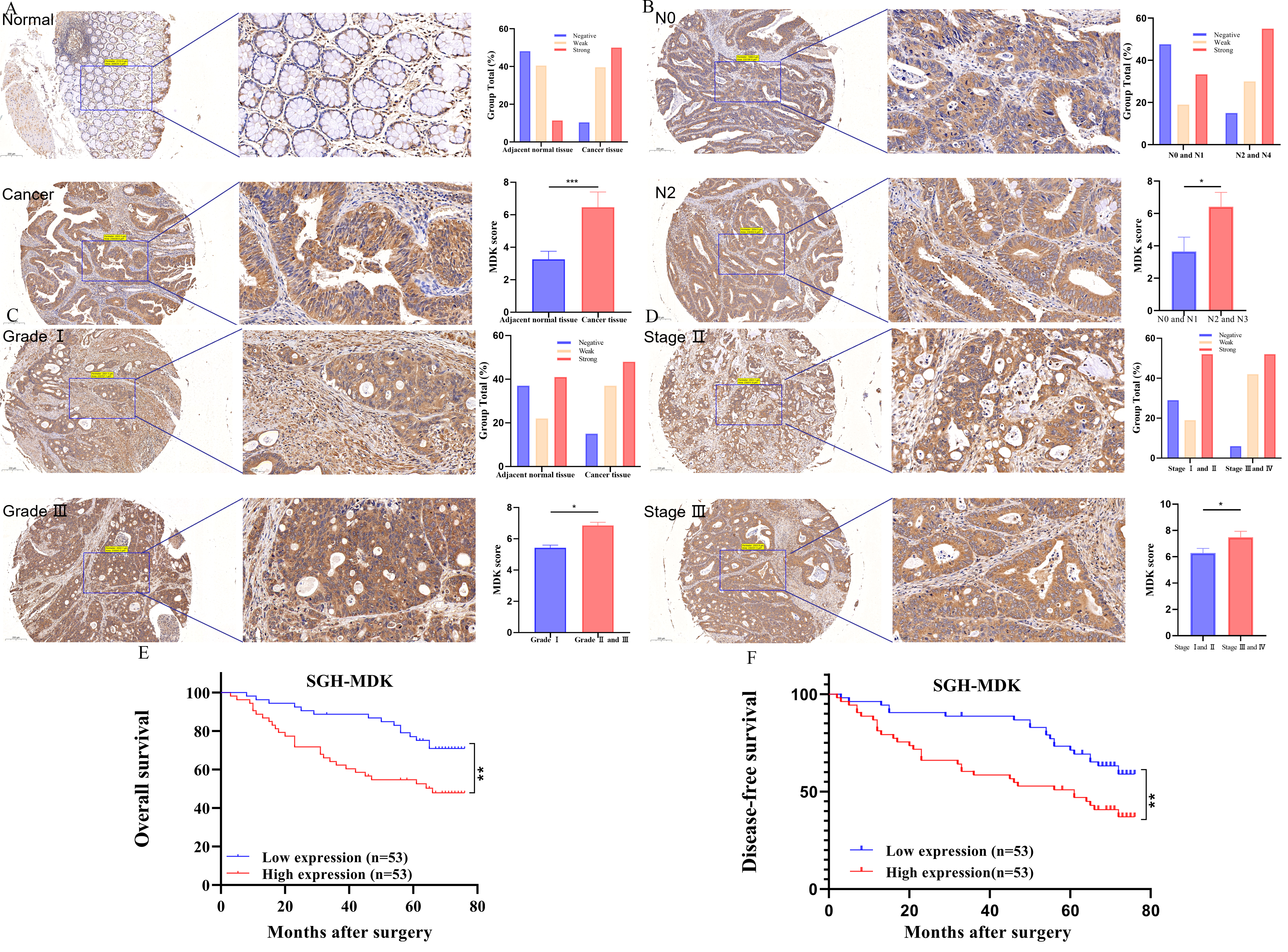

Supplement: Supplementary file 1 [file cancers-18-01311-s001.zip › Supplementary Figure S3.tif]

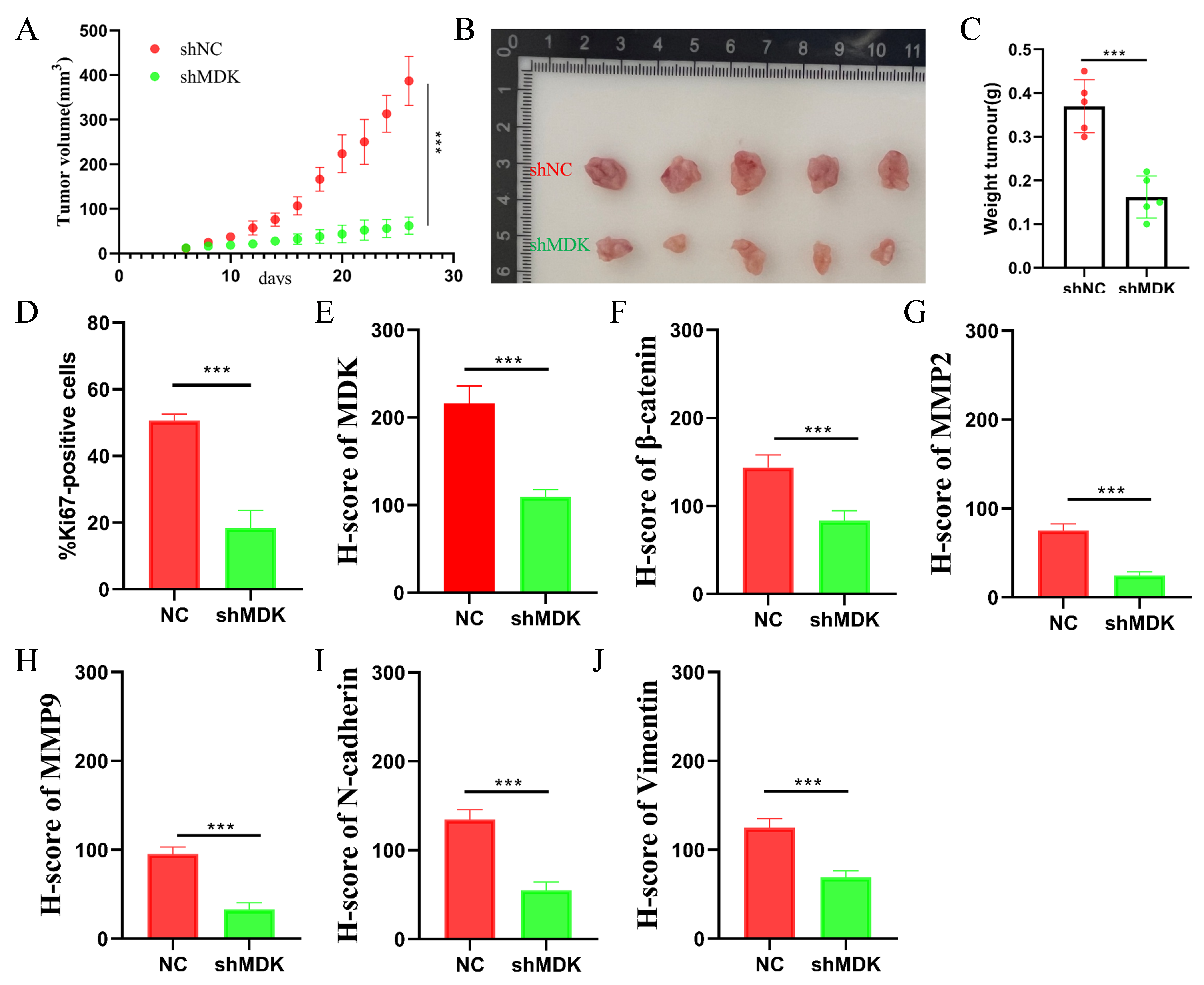

Supplement: Supplementary file 1 [file cancers-18-01311-s001.zip › Supplementary Figure S5.tif]
